# The CLOSED trial; CLOnidine compared with midazolam for SEDation of paediatric patients in the intensive care unit: study protocol for a multicentre randomised controlled trial

**DOI:** 10.1136/bmjopen-2017-016031

**Published:** 2017-06-21

**Authors:** Antje Neubert, Manuel Alberto Baarslag, Monique van Dijk, Joost van Rosmalen, Joseph F Standing, Yucheng Sheng, Wolfgang Rascher, Deborah Roberts, Jackie Winslade, Louise Rawcliffe, Sara M Hanning, Tuuli Metsvaht, Viviana Giannuzzi, Peter Larsson, Pavla Pokorná, Alessandra Simonetti, Dick Tibboel, CLOSED Consortium

**Affiliations:** 1 Department of Children and Adolescents Medicine, Friedrich-Alexander-University Erlangen/Nuremberg, Erlangen, Germany; 2 Intensive Care and Department of Pediatric Surgery, Erasmus MC-Sophia Children’s Hospital, Rotterdam, The Netherlands; 3 Department of Biostatistics, Erasmus MC, Rotterdam, The Netherlands; 4 Institute of Child Health, University College London, London, United Kingdom; 5 Therakind Ltd, London, United Kingdom; 6 UCL School of Pharmacy, University College London, London, United Kingdom; 7 Clinic of Anaesthesiology and Intensive Care, Tartu University Hospital, Tartu, Estonia; 8 Fondazione per la Ricerca Farmacologica Gianni Benzi onlus, Valenzano, Italy; 9 Department of Pediatric Anesthesia and Intensive Care, Astrid Lindgrens Childrens Hospital, Karolinska University Hospital, Stockholm, Sweden; 10 Department of Pediatrics, First Faculty of Medicine, Charles University Prague, Prague, Czech Republic; 11 Clinical Trial Center, Bambino Gesù Children’s Hospital, Rome, Italy

**Keywords:** sedation, children, criticalillness, paediatrics, pharmacology

## Abstract

**Introduction:**

Sedation is an essential part of paediatric critical care. Midazolam, often in combination with opioids, is the current gold standard drug. However, as it is a far-from-ideal agent, clonidine is increasingly being used in children. This drug is prescribed off-label for this indication, as many drugs in paediatrics are. Therefore, the CLOSED trial aims to provide data on the pharmacokinetics, safety and efficacy of clonidine for the sedation of mechanically ventilated patients in order to obtain a paediatric-use marketing authorisation.

**Methods and analysis:**

The CLOSED study is a multicentre, double-blind, randomised, active-controlled non-inferiority trial with a 1:1 randomisation between clonidine and midazolam. Both treatment groups are stratified according to age in three groups with the same size: <28 days (n=100), 28 days to <2 years (n=100) and 2–18 years (n=100). The primary end point is defined as the occurrence of sedation failure within the study period. Secondary end points include a pharmacokinetic/pharmacodynamic relationship, pharmacogenetics, occurrence of delirium and withdrawal syndrome, opioid consumption and neurodevelopment in the neonatal age group. Logistic regression will be used for the primary end point, appropriate statistics will be used for the secondary end points.

**Ethics:**

Written informed consent will be obtained from the parents/caregivers. Verbal or deferred consent will be used in the sites where national legislation allows. The study has institutional review board approval at recruiting sites. The results will be published in a peer-reviewed journal and shared with the worldwide medical community.

**Trial Registration:**

EudraCT: 2014-003582-24; Clinicaltrials.gov: NCT02509273; pre-results.

**Strengths and limitations of this study**

- The dosing scheme being used in this trial is based on detailed pharmacokinetic (PK)/pharmacodynamic modelling data.
- Pain and sedation are measured using a standardised assessment scheme—(COMFORT-B score)—across all participating units.
- Besides information on the efficacy and safety of clonidine in critically ill children, this trial will provide population-based PK information in this specific patient population.
- Age-specific formulations are being used in this trial, which can be marketed afterwards.
- A previous similar trial indicated challenging recruitment, this could be a potential threat to this trial.

## Introduction

### Unlicensed and off-label drug use

In Europe, <30% of marketed drugs include results from paediatric clinical trials and other information on paediatric use in their documentation (Summary of Product Characteristics (SPC) or Product Leaflet).^1^ This results in widespread off-label use in paediatrics, especially in the case of old drugs that have never received a paediatric authorisation. Off-label paediatric use (including all the uses not listed in the SPC^2^) in Europe accounts for 45%–60% of the total number of prescriptions, with rates of up to 90% in patients admitted to neonatal (NICU) or paediatric intensive care units (PICU).^3^

The entry into force of the Paediatric Regulation in 2007^4^ gave an important stimulus to support the development of medicines for children by introducing a specific measure to favour work on off-patent medicines, the so-called Paediatric Use Marketing Authorisation (PUMA). A PUMA application should include the submission of paediatric data in accordance with an agreed Paediatric Investigation Plan (PIP).

According to article 40 of the Regulation, the European Commission (EC) reserved funds ‘to develop off-patent medicinal products with recognised therapeutic interest for children and included in a ‘Priority List’ adopted by the European Medicines Agency (EMA) through its Paediatric Committee (Seventh Framework Programme for Research HEALTH—(2007–2013) Programme area—topic 4.2–1). Among 20 projects approved,^5^ the CLOSED project was granted with the aim to develop an age-appropriate formulation of clonidine for sedation in PICU, in line with the EMA ‘Revised Priority List for Studies into Off-Patent Paediatric Medicinal Products’ 2012.^6^ In order to specifically meet this therapeutic need, a comprehensive development plan in the form of a PIP was submitted to the EMA Paediatric Committee (PDCO) in July 2012 and approved in January 2013. In March 2015, a modification of the PIP was proposed after the finalisation of the clinical study protocol. This modification was approved by the PDCO in August 2015.

### Analgosedation in PICU

Approximately 2% of all paediatric patients admitted to hospital require treatment in PICU.^7^ Most PICU admissions are unplanned emergencies, mainly in the context of congenital heart diseases (40%), respiratory diseases (20%), major trauma (15%) and neurological problems other than trauma (<10%).^8^

Often, mechanical ventilation is required for facilitating recovery after major surgery or for treating respiratory failure. In most of these cases, analgosedation using potent opioids and sedatives is mandatory to reduce metabolic rate and oxygen demand, assist mechanical ventilation, avoid inadvertent self-extubation and lower anxiety and distress.^9 10^ To this effect, a variety of agents are used in NICU and PICU, such as opioids, GABA-receptor agonists, N-methyl-d-aspartate receptor (NMDA)-receptor antagonists and α2-receptor agonists.^11^

### Opioids

The main opioids used in PICU and NICU are morphine, fentanyl and remifentanil. Opioids bind to the μ-receptor in the central nervous system (CNS), and provide analgesia as well as sedation.

### GABA-receptor agonists

GABA receptors have an inhibitory effect on the CNS. Benzodiazepines agonise these receptors and therefore have sedative, anxiolytic and anticonvulsant properties. Disadvantages are paradoxal reactions (agitation and confusion) and tolerance and withdrawal after prolonged use.

Propofol is a unique agent with GABA-ergic properties as well as anti-NMDA and sodium channel blocking effects. Propofol is not suitable for long-term sedation, due to the perceived risk of propofol infusion syndrome, a metabolic derangement accompanied by severe metabolic acidosis, hyperkalaemia, hyperlipidaemia, rhabdomyolysis and organ failure, associated with an increased risk of mortality.^12^

### Midazolam

Midazolam is the most widely used sedative agent in PICU. It is a short-acting benzodiazepine (T_max_ of 5–10 min after intravenous injection) with a half-life of 1–3 hours (up to 12 hours in neonates). Besides sedation and anxiolysis, midazolam also provides anterograde amnesia, thus minimising children's recall of unpleasant experiences after a PICU admission.^13^ Midazolam is mainly metabolised to the equipotent metabolite 1-OH-midazolam (1-OH-MDZ), and then glucuronidated to the renally excreted 1-OH-MDZ-glucuronide. Its main adverse effects include tolerance, dependence and withdrawal syndrome following discontinuation.

### Clonidine

Clonidine agonises the α2-adrenergic receptor. The reduced sympathetic outflow^14^ in the CNS results in sedation, anxiolysis and analgesia.^15 16^ Because of its analgesic properties, it has been used as an adjunct in surgical procedures as premedication or as a supplementary agent in regional anaesthesia.^17^ The reduction of sympathetic outflow associated with clonidine may have specific benefits in critically ill children. A2 agonists can improve neurological outcome associated with ischaemic cerebral injury,^18–20^ the mechanism of action of which is unclear but may be due to suppression of extracellular glutamate and aspartate release during energy failure.^21^ Recent data have also demonstrated that preconditioning before the insult can both reduce infarct size and improve neurological outcome after the insult.^22^ Surgery and critical illness are associated with a variety of stress responses, which can result in organ dysfunction:^23^ renal function deteriorates after both adult and paediatric cardiac surgery, and this effect is due in part to the increase in sympathetic outflow and the rise in circulating vasoconstrictors such as norepinephrine, vasopressin and angiotensin.^24 25^ Clonidine has been demonstrated to suppress these responses and prevent the associated decline in renal function after adult cardiac surgery.^26^ In addition, clonidine has independent local effects on tubular function which promote both diuresis and natriuresis.^27^ In terms of cardiovascular responses, reduction in stress responses by α2-agonists have been shown to reduce perioperative myocardial ischaemia in adults undergoing cardiac and non-cardiac surgery.^28^ As the mechanism of action of clonidine is very different from the GABA-agonist midazolam, the hypothesis is postulated that it may be less associated with paediatric delirium and withdrawal syndrome. Moreover, clonidine is one of the agents used for the treatment of withdrawal syndrome.^29^

### Previous clinical trials

Clonidine is commonly used off-label in paediatric anaesthesia and intensive care medicine. Its use is recommended for the sedation of critically ill children in PICUs by the UK and German consensus guidelines^12 30^ and local hospital guidelines across Europe. Unfortunately, despite this widespread use of clonidine, there are limited data on efficacy, dose requirement and safety when used for sedation on PICUs. A number of studies have been performed,^31–35^ however, to overcome this knowledge gap (see table 1 for a short overview).

**Table 1:** An overview of paediatric studies involving clonidine for sedation in the intensive care unit

Study	Sample size and age	Design	Outcome
Ambrose *et al*.^31^	n=30, 0–10 years	Three-step: intravenous Low-dose vs high-dose (variable dose together with midazolam), third group fixed dose	No adverse effects on haemodynamics, sufficient sedation in combination with midazolam
Arenas-Lopez *et al*.^32^	n=24, 0–5 years	Prospective cohort study, oral clonidine as additive to morphine/lorazepam	Opioid-sparing and benzodiazepine-sparing, safe and effective
Wolf *et al*.^33^	n=129, 0–15 years	Double-blind, randomised controlled trial of intravenous clonidine vs midazolam	No difference in effectivity, underpowered due to recruitment problems
Hünseler *et al*.^34^	n=219, 0–2 years	Double-blind, randomised controlled trial of intravenous clonidine vs midazolam	Opioid-sparing and benzodiazepine-sparing in neonatal age group
Duffett *et al*.^35^	n=50, 0–18 years	Double-blind, randomised controlled trial of oral clonidine vs placebo in addition to physician-driven sedation	No significant difference in effectivity, study with clonidine clinically feasible

*In summary, when used in addition to standard sedation management, clonidine has a benzodiazepine-sparing and opioid-sparing effect. Comparative studies showed equivalence, but were either underpowered^33^ or set up as a pilot trial.^35^

## Study aims

For the purpose of sedation of intubated and mechanically ventilated paediatric patients, this clinical study sets out to provide the data needed for a PUMA application for the use of clonidine in PICU patients:

- Data on efficacy and safety of clonidine compared with midazolam.
- Pharmacokinetic (PK) data on both clonidine and midazolam in critically ill children and adolescents, using a population-based approach.

Together with the development of age-adapted and weight-adapted formulations of clonidine, as part of this study, data on quality and stability of these new formulations will also be generated.

## Methods/design

### Study design

The CLOSED study is a multicentre, double-blind, randomised, active-controlled non-inferiority trial with a 1:1 randomisation between clonidine and midazolam. Both treatment groups are stratified according to age in three groups with the same size: <28 days (n=100), 28 days to <2 years (n=100) and 2–18 years (n=100). The primary end point is defined as sedation failure within the study period.

### Patient recruitment

Patients in five different European Union (EU) member states (Czech Republic, Germany, Italy, The Netherlands and Sweden) will be recruited. Patients (neonates ≥34 gestational weeks and children <18 years) eligible for inclusion in the trial, according to inclusion and exclusion criteria, will be screened for possible enrolment (see table 2 for inclusion and exclusion criteria).

**Table 2:** Inclusion and exclusion criteria for the CLOSED trial

Inclusion criteria	Exclusion criteria
Aged from birth (GA ≥34 weeks) to <17 years, 11 months and 1 week	Body weight <1200 g or GA <34 weeks
(Expected) admission to PICU	Body weight ≤3 kg AND age ≥28 days Body weight <10 kg AND age ≥2 years Body weight >85 kg
(Expected) indication for mechanical ventilation (both invasive and non-invasive)	Clonidine within last 7 days prior to admission* Sedation >72 hours prior to screening
Anticipated need for continuous sedation ≥24 hours	Known hypersensitivity to clonidine, midazolam, morphine or propofol or any of their formulation ingredients and their rescue medication
Informed consent (or deferred consent if applicable)	Administration of continuous muscle relaxants or other contraindicated drugs
Informed assent if applicable	Postresuscitation within 24 hours or therapeutic whole-body hypothermia
	CPAP or ECMO treatment
	Severe organ insufficiencies: Renal failure according to pRIFLE^63^ or nRIFLE^64^ criteria Cardiac failure as defined by modified Ross class 3 or 4 Arterial hypotension according to guidelines^62^ Circulatory failure as defined by Goldstein criteria^59^†
	Traumatic brain injury or other intracranial pathology including mental retardation and status epilepticus
	Phaeochromocytoma, acute asthma or paralytic ileus‡
	Severe bradycardia
	Pregnancy
	Known arterial hypertension in medical history
	Previous participation in this trial at any time or previous participation in drug trial within last 3 weeks
	Declined informed consent from parent(s)/legal guardian(s)

*This exclusion criteria has been removed in the second protocol amendment.

†These criteria have been modified in the second protocol amendment and allow for use of inotropes/vasopressor drugs.

‡The exclusion criteria acute asthma and paralytic ileus have been removed in the second protocol amendment.

CPAP, continuous positive airway pressure; ECMO, extracorporeal membrane oxygenation; GA, gestational age; nRIFLE, Neonatal Risk, Injury, Failure, Loss of Function and End-stage renal disease; pRIFLE, Paediatric Risk, Injury, Failure, Loss of Function and End-stage renal disease.

Surgery schedules will be screened on a daily basis for possible patients expected to be admitted to the intensive care unit (ICU). These patients and parents will be identified, approached, informed and enrolled prior to surgery by study responsible doctors and/or nurses. Patients eligible for inclusion transferred to the centres will be identified by the transport team. Patients admitted to the PICUs will continuously be screened by PICU medical staff to identify patients possible for enrolment. Parents of these eligible patients will be approached, informed and enrolled by study responsible doctors and/or nurses.

Children are often admitted to PICU/ NICU in an emergency setting. Urgent action will most likely be required and the subject's parent(s)/legal guardian(s) may not be immediately available to give consent. An example of this is when a critically ill baby is delivered by caesarean section, the mother may still be under anaesthesia while another parent/legal guardian of the child may not be present. Therefore, given the nature of the trial, especially considering that ICU patients are often admitted as a result of emergency, deferred consent/assent, as described in Article 35 of the new European Regulation on clinical trials (EU) No 536/2014 of the European Parliament and of the Council,^36^ is foreseen if allowed by local legislation, as described in section ‘Ethical considerations’. This allows for the child to be enrolled in the trial before the parent(s)/legal guardian(s) are able to give consent, which is then sought once they are available. If, subsequently, deferred consent is withheld, the subject is removed from the study but a record will remain in the subject's medical notes.

### Study treatments

First, it was necessary to define the dosing scheme so that appropriate drug concentrations could be used to maintain double blinding. A plasma clonidine concentration of around 2 μg/L gave adequate sedation combined with morphine infusion in ventilated PICU patients,^32^ and 2 μg/L clonidine concentration also drops the Bispectral Index to 71 in adults.^37^ Clonidine has a long elimination half-life (16.9 hours in neonates, 11.4 hours in infants and 7.4 hours in children^38^), so loading doses are proposed. It was therefore decided that loading and maintenance doses aimed at achieving steady-state concentrations of around 2 μg/L would be aimed for, and this scheme was designed by a PK/pharmacodynamic (PD) expert working group within the CLOSED consortium. Based on these doses, a similar dose scheme of midazolam was designed that would maintain double blinding.

The agreed dosing scheme consists of 15 min loading doses followed by a maintenance infusion rate. Increases or decreases, based on the evaluation of COMFORT-B and Nurses Interpretation of Sedation Score (NISS) score, in infusion rate are permitted, and preceding any increase, a loading dose over 15 min is specified. There are also compulsory lock-out times between each dose escalation or de-escalation to allow for new steady-state conditions to be approached. Participants will follow the dosing regimen, with 1 unit meaning 1 μg for clonidine, or 100 μg for midazolam:

- First bolus (T=0). Initial loading dose is 2 units/kg over 15 min and the following continuous infusion 1 unit/kg/hour will be administrated to all patients followed by 15 min lock-out period.
- Second bolus. If insufficient sedation after the fir st lock-out period, the second loading dose is 2 units/kg over 15 min and the following continuous infusion 1 unit/kg/hour will be administrated with a 15 min lock-out period.
- Third bolus. If insufficient sedation after the second lock-out period, the third loading dose is 2 units/kg over 15 min and the following continuous infusion 1.5 units/kg/hour will be administered with a minimum of 105 min lock-out period.
- Fourth bolus. If insufficient sedation after the third lock-out period, the fourth loading dose is 2 units/kg over 15 min and the following continuous infusion 2 units/kg/hour will be administered followed by a 105 min lock-out period.
- Fifth bolus. If insufficient sedation after the fourth lock-out period, the fourth loading dose is 2 units/kg over 15 min and the following continuous infusion 2 units/kg/hour will be administered with 24 hours lock-out period.
- Sixth to 11th bolus. If insufficient sedation after 24 hours lock-out period, the 6th–11th loading dose of 2 units/kg over 15 min and the following continuous infusion 2 units/kg/hour would be administered with at least 24 hours lock-out period between.

**Figure 1:**
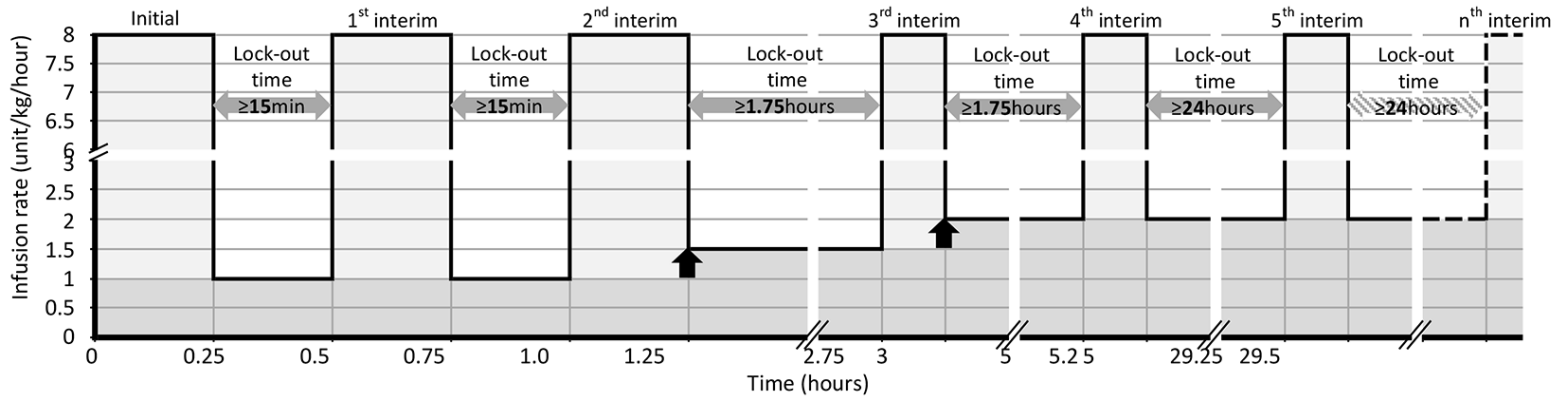
Minimum time line of infusion rate increases for all loading doses of IMP administered for subjects (half doses in neonates).

All doses are halved in patients younger than 1 month. Figure 1 shows the scenario of all loading doses of study drug administered for subjects.

Decreasing the maintenance dose will be possible in the case of oversedation (ie, COMFORT-B >22 OR COMFORT-B=11–22 and NISS=3).

Following steps will be taken to decrease the dose (doses are halved in neonates):

- 2 units/kg/hour will be decreased to 1.5 units/kg/hour.
- 1.5 units/kg/hour will be decreased to 1 unit/kg/hour.
- 1 unit/kg/hour will be decreased to 0.75 units/kg/hour.
- 0.75 unit/kg/hour will be decreased to 0.5 units/kg/hour.

If patients are still oversedated with the minimum dose, the Investigational Medicinal Product (IMP) administration will temporarily be stopped until patients need sedation again, and they will start back at T=0.

During the preparation of the trial, the consortium faced one unknown challenge. In V.1.0 of the protocol, dose reduction should be performed when a child is oversedated. After 30 min, a new sedation assessment should be performed and acted on the same. In an oversedated child, this could lead to a child being without IMP after 90 min (three dose reductions). This is certainly not a desirable situation, but not unthinkable as the half-life of both drugs are longer than 90 min. Therefore, a 6-hour observation period has been implemented. After a dose reduction, it is recommended to wait for 6 hours before the next dose reduction.

In this same amendment, another problem has been mitigated. Version 1.0 did not provide clear information about increasing the dose again after a prior decrease. In the amendment, it has been made clear that subjects having a decrease of maintenance infusion to <1 unit/kg/hour will return to 1 unit/kg/hour, irrespective of the current infusion rate. If the decrease has been made while increases before have led to a maintenance infusion rate of ≥1 unit/kg/hour, reincrease will be following the precedent step (eg, if a decrease has taken place from 1.5 to 1 unit/kg/hour, reincreasing will return the rate back to 1.5 unit/kg/hour).

### Pharmaceutical development

To overcome the potential for administration errors associated with commercially available clonidine ampoules, three different strengths of clonidine HCl—low (250 μg/50 mL), medium (500 μg/50 mL) and high (2500 μg/50 mL)—have been developed. Based on the dosing regimen described above, the concentrations of the low, medium and high strengths of midazolam (25 mg/50 mL, 50 mg/50 mL and 250 mg/50 mL) were 100-fold higher than for clonidine HCl. For study blinding purposes and to avoid dosing errors, a simple three-colour scheme based on strength will be used.

To avoid the administration of preservatives, all formulations will be stored in single-use glass phials, with any contents remaining after 24 hours to be discarded. A 50 mL volume has been selected to limit the number of children requiring more than one phial in 24 hours (only subjects weighing over 46 kg and receiving the maximum dose in 24 hours will require two phials in a 24-hour period).

### Efficacy end points

Level of sedation was evaluated using the COMFORT-B score (ranging from 6 to 30), which is a validated scoring system commonly used in PICU.^39^ Level of sedation was also evaluated by NISS when the COMFORT-B score fell between ≥11 and ≤22. NISS is the nurse's expert opinion of the level of sedation (1: insufficient sedation; 2: adequate sedation; 3: oversedation). A subject is considered to be undersedated in cases of COMFORT-B score of >22; or COMFORT- B score of 11–22 in combination with NISS of 1.

In addition to COMFORT-B score (and NISS where appropriate), pain was assessed using the Numerical Rating Scale (NRS) at the same time intervals. The 11-point NRS is a global pain rating scale with which the nurse rates pain intensity by number (0=no pain and 10=worst imaginable pain).^40^ In this study, to simplify matters, the NRS score was addressed first, so, if the pain score was ≥4, morphine treatment was escalated while sedation treatment remained the same, regardless of the COMFORT-B score. This means that any pain score took precedence over the sedation score.

### Primary objective

To assess the non-inferiority of the sedative properties of continuous intravenous clonidine compared with continuous intravenous midazolam in mechanically ventilated children and adolescents (0–18 years) admitted to a PICU.

### Primary end point

The primary end point is defined as sedation failure within the study treatment period (a maximum of 7 days).

Sedation failure is defined as:

When a subject's assessment results are:

NRS score <4 and COMFORT B score >22 OR

NRS score <4 and COMFORT B score ≥11 and ≤22 AND NISS score 1 at a point during the study where no further increase in IMP dose are permitted as described in the dose-escalation scheme.

In summary, there are two possible outcomes (success or failure) of the primary end point.

### Secondary objectives

To evaluate the safety and tolerability (including withdrawal effects) of clonidine compared with midazolam in ventilated children and adolescents admitted to PICU.

To determine clonidine dose-dependent effects on sedation.

To establish the PK/PD relationship of clonidine for sedation in PICU.

To compare the cumulative total morphine consumption/kg between the two arms in the first 48 hours of IMP administration.

### Secondary end points

Primary PK parameters estimated will be clearance (CL), volume of distribution (VD) and intercompartmental clearance (Q). PK measurements will be made using sparse opportunistic sampling.

PK/PD modelling will seek to elucidate the relationship between IMP PKs and sedation as measured by COMFORT-B score.

The PK/PD covariate model will include demographics (eg, age, weight), clinical characteristics (eg, reason for admission) and pharmacogenomics (see the 'Pharmacogenomic end points' section).

### Safety and tolerability assessments

Safety and tolerability assessments include:

- Extent of withdrawal effects using the Sophia Observation withdrawal Symptoms-Paediatric Delirium (SOS-PD) scale measured three times a day in subjects who receive sedatives and/or opioids for 5 days or more and after cessation of treatment in all subjects for at least 24 hours after treatment. The extent of delirium measured by the SOS-PD scale.^41^
- Rebound hypertension monitored for at least 72 hours postcessation of treatment.
- Percentage of respiratory depression per group.
- Adverse event reporting of symptoms indicative of post-ICU stress (eg, nightmares, confusion, hallucinations).
- Neurodevelopment of subjects recruited in lower age group (from birth to 27 days) at 12 months after cessation of IMP, as measured using the Bayley II score.^42^

### Effect size justification

The primary objective of this study is to evaluate the efficacy of clonidine compared with midazolam in the sedation of ventilated children and adolescents admitted to PICU, with a view to demonstrate non-inferiority of clonidine.

Every patient enrolled will be in need of sedation, thus a placebo cannot be considered. Intravenous midazolam is the standard treatment for long-term sedation in children. It is licensed in this population, regarded as standard of care and recommended by treatment guidelines.^12 30^

Based on clinical experience and the limited data available regarding the use of clonidine as a sedative agent in PICU, it is assumed that the sedation success rate for clonidine is higher than for midazolam. The estimated difference in success rate between the two drugs was assumed to be 5%.

The primary end point is the success or failure of the subject's sedation treatment. Failure is defined as inadequate sedation with no further dose increases allowed in the dose titration scheme. This will be assessed by a nurse or physician using the COMFORT-B sedation assessment and, if appropriate, NISS. These assessment scales are open to a degree of subjectivity and it is therefore considered essential to have a double-blind study design to minimise bias due to knowledge of treatment group by the assessor.

**Figure 2:**
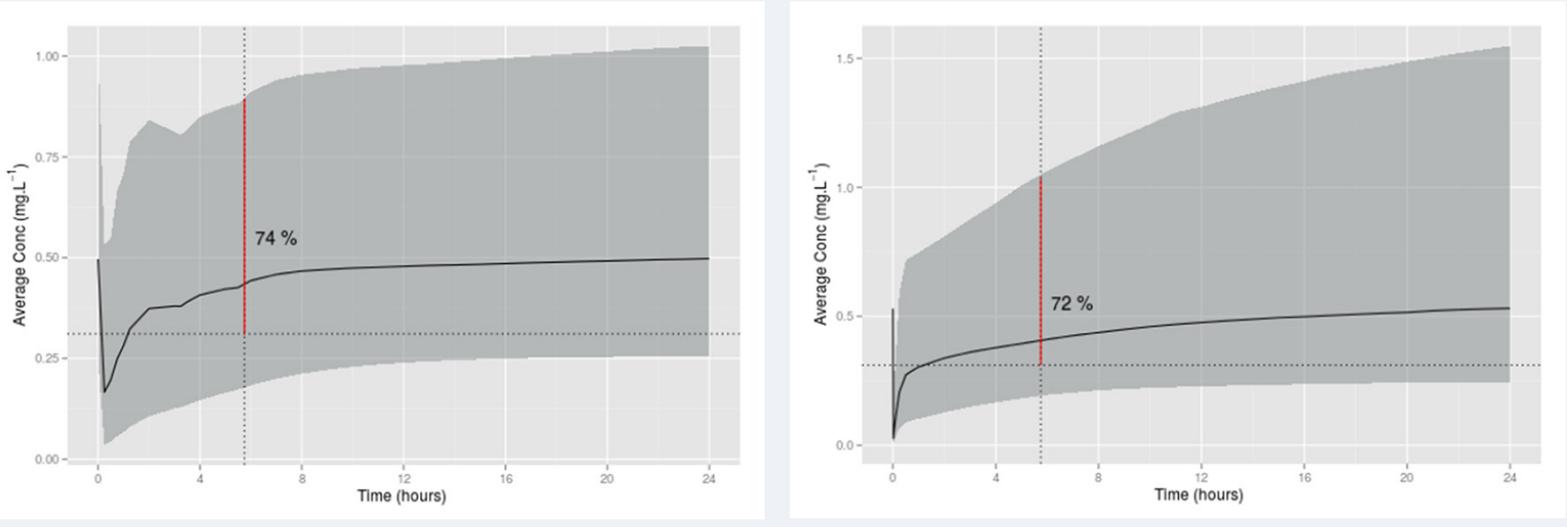
The proportion of average midazolam plasma concentration above the target value (0.311 mg/L) after the time of 5th bolus according to the proposed dose scheme(left) and the maximum Dutch recommended dosing regimen(right).

In order to define the effect size, the expected success rate for midazolam was needed. The target concentration for midazolam was taken from the paper by de Wildt *et al* 43 showing around 311 μg/L is required for sedation.^43^ The maximum midazolam dose of 200 (100–200) μg/kg bolus followed by 200 (50–200) μg/kg/hour for all age groups (including neonates) is recommended by Dutch clinical consensus guideline.^44^ According to the proposed dosing schedule, the exposure of midazolam is similar to the Dutch recommended dosing regimen from the simulation (figure 2).

Furthermore, after the fifth bolus according to the proposed dose scheme, the proportion of average midazolam plasma concentration above the target value (0.311 mg/L) is 74% and from the Dutch dose scheme is 72%. Thus, we assumed that the sedation success rate in the midazolam arm given our dosing scheme would be 75%, and in the clonidine group this would be 80%.

### Sample size calculation

Given this assumed effect size of a 75% success rate for midazolam, a sample size of 258 (129 per treatment arm) provides at least 80% power to show non-inferiority of clonidine compared with midazolam. The sample size calculation is based on a logistic regression for the primary efficacy end point of sedation success, with adjustment for study arm, age category and baseline COMFORT-B score. Non-inferiority of clonidine requires that the lower limit of the one-sided 97.5% CI for the OR of sedation success using clonidine (vs midazolam) is at least 0.583, which is equivalent to a 10% non-inferiority margin in case of a sedation success rate of 80%. The required sample size was calculated using simulation.

It is assumed that the total drop-out rate will be <13% and hence 300 subjects will be recruited, 150 in each arm and 100 in each age subgroup. The sample size of 300 subjects also provides at least 70% power to prove non-inferiority with respect to sedation success rate for each age subgroup (>90 subjects per age subgroup). Since the sample size for the age subgroup analyses is limited, a 15% non-inferiority margin at a one-sided alpha level of 0.025 is used, with an assumed sedation success rate of 80% in the midazolam group and 85% in the clonidine group. Subgroup analyses will be performed using a CI for the Mantel-Haenszel risk difference, with stratification by centre.

### Statistical analysis

### Analysis populations

The safety evaluation set (SES) is the subset of all subjects who were randomised into the trial and exposed to study medication (also referred to as the intention-to-treat (or ITT) population). Safety analyses will be performed in the SES.

The full analysis set (FAS) is the subset of subjects in the SES for whom the primary efficacy variable is available. Data analysis for the efficacy end points will be performed in the FAS.

The per protocol set (PPS) is the subset of subjects in the FAS without major protocol deviations. Major protocol deviations will be defined during the Blinded Data Review Meeting.

The PK/PD analysis set (PKS) is the subset of subjects in the SES with evaluable PK samples defined as drug concentration measurements.

### Primary efficacy analysis

Logistic regression of the primary end point, sedation failure, with treatment group, baseline sedation assessment (ie, the baseline COMFORT-B score), centre and age group as covariates, at a one-sided significance level of alpha=2.5% will be used. The statistical hypotheses are the following:

H0: OR≤ δ_OR_ and H1: OR>δ_OR_,

OR=p_C_*(1−p_M_)/((1−p_C_)*p_M_), where p_C_ and p_M_ are probabilities of sedation success in the clonidine group and midazolam group, respectively; δ_OR_ is the non-inferiority margin which is predefined as 0.583.

H0 will be rejected if the lower bound of the one-sided 97.5% Wald CI for the OR of sedation success proportion will be >0.583. The primary analysis will be based on the ITT principle. The primary end point will be evaluated for FAS. An equivalent analysis for the PPS will be performed as a sensitive analysis. No interim analyses are planned for the primary end point.

### Secondary efficacy analyses

All secondary efficacy analyses will be conducted on FAS. Secondary efficacy end points will be analysed in detail as follows:

- The proportion of subjects with sedation success between treatments within each age group will be based on the Mantel-Haenszel risk difference, with stratification by centre.^45^ A 15% non-inferiority margin at a one-sided alpha level of 0.025 will be used.
- The aggregate intervals (ie, the total time) (based on the planned assessment points) with COMFORT-B score >22 or <11 during treatment period between treatment groups were analysed using the Mann-Whitney U test.
- The proportion of subjects with COMFORT-B score ≤11 between treatment groups and within each age group will be analysed using the χ^2^ test or Fisher's exact test.
- The morphine consumption in units of μg/kg/day during the first 24 hours of IMP administration and for the remaining study period between treatment groups will be analysed using linear regression analysis, with adjustment for centre and age group. An appropriate transformation of morphine consumption may be used to ensure normality of the residuals in the linear regression model.

An age subgroup analysis will be performed for each age group, using centre as a stratification variable.

### PK/PD analyses

The primary objective of PK/PD analyses in this trial is to evaluate the pattern and extent of covariates affecting the PK/PD profiles of clonidine and to provide the information of optimal dose for patients based on their particular age and other related covariates for future clinical practice. Three PK/PD interim analyses will be undertaken, whereby samples will be shipped and assayed for drug concentrations in order to ensure the systems for sample transport and analysis, along with data linkage to the electronic Case Record Form (eCRF) are fully operational, and to allow preliminary PK/PD model development. PK/PD interim analyses will be conducted after 15, 100 and 200 subjects will complete the study.

PK/PD data will be modelled using the Fortran-based non-linear mixed effects software NONMEM. Typical PK dispositional compartmental models will be tested, along with investigation of linear and non-linear elimination. Multivariate covariate analysis will be undertaken to investigate the impact of subject factors on PK model parameters. In particular, we will focus on the effect of body size with allometric models, and age (both postmenstrual and postnatal) possibly using literature prior models in order to delineate the effect of size and age from other factors. We will also look at the impact of drug metabolising enzyme genotype on interindividual variability. The link between PK and PD (COMFORT-B score) will be investigated by sequential and simultaneous modelling, possibly including an effect compartment. The Item Response Theory (IRT) will also be considered for PK/PD modelling, whereby effect is considered an unobserved normally distributed latent variable, and each item score of COMFORT-B is allowed to contribute to this through link functions.^46^ Since subjects will receive other sedative agents for procedures, the concentrations of which may not be measured, a PK/PD approach will be taken to model sedation requirements in this period.

Mixed effects models will be fitted with maximum likelihood and addition of a single fixed effect will be guided by improvement of fit using the likelihood ratio test. Model evaluation will consist of goodness-of-fit (residual plots) and simulation-based diagnostics (visual predictive check), and parameter precision and robustness will be investigated with non-parametric bootstrapping.

### Safety analyses

All safety analyses will be performed on the SES. Analyses will include the following end points:

- Adverse Event (AE)s, Serious Adverse Event (SAE)s and Suspected Unexpected Serious Adverse Reaction (SUSAR)s.
- Deaths.
- Clinical laboratory evaluations and vital signs. Descriptive summaries of laboratory values (including clinical chemistry, haematology, coagulation and urinalysis variables) and changes from baseline throughout the study will be generated. Shift tables will also be used for comparing changes from baseline and the proportion of subjects experiencing abnormalities between treatment groups.
- The SOS scores will be descriptively summarised and analysed with a linear mixed model (with between-arm differences at baseline constrained to be 0).
- Neurodevelopment will be evaluated using the Bayley Scales of Infant and Toddler Development. The scale will be descriptively summarised and compared between treatment groups using linear regression analysis, with adjustment for centre and age group.

Quantitative variables will be by the number of subjects analysed (N), mean, median, minimum and maximum. Categorical variables will be analysed by frequencies and percentages for each category.

## Randomisation and kit assignment

Randomisation is stratified by both age subset and clinical site. Each stratum consists of 99 prerandomised numbers (randomisation numbers), assigned to either clonidine or midazolam. These randomisation lists were prepared by a statistician not otherwise involved in the study, with the use of blocked randomisation consisting of small variable block sizes, in order to maintain blinding and conceal allocation.

At each clinical site, the kits marked with the random numbers for each age subset will be assigned to subjects in the order they are enrolled into the study. Each subject kit will contain two IMP boxes of seven phials, in order to supply the subject with enough medication for the maximum 7-day study period. The contents of each subject kit is outlined in table 3.

**Table 3:** Composition of the subject kit for each age group

Age subset	Age group	Formulation strength	Subject bodyweight	Number of boxes
1	<28 days	Low Medium	≤3 kg >3 to <10 kg	1 1
2	28 days to <2 years	Medium High	>3 to <10 kg ≥10 kg to <47 kg	1 1
3	≥2 years to <18 years	High	≥10 kg to 85 kg	2 (in case ≥47 kg)

Emergency unblinding will be possible as for each randomised subject kit, two complete sets of sealed emergency envelopes have been prepared (one set for safekeeping by the investigator, the other will be delivered to the group responsible for overseeing pharmacovigilance of the study). Unblinding of the treatment allocation for a subject will be performed in an emergency that, in the opinion of the investigator, is warranted for a given subject for safety reasons.

## PK/PD end points

PK/PD analyses will be carried out as a secondary end point to give further information on the dose-concentration-effect relationship primarily of clonidine, and also of midazolam. The PK/PD model will be used to evaluate the dose scheme used in the study, and if necessary recommend an updated dose scheme for clinical use. PK/PD data will be modelled using non-linear mixed effects modelling. The impact of drug metabolising enzyme genotype on interindividual PK/PD variability will be considered alongside other demographics in the multivariable covariate analysis. The link between PK and PD (COMFORT-B score) will be investigated by sequential and simultaneous modelling, possibly including an effect compartment. IRT will be explored for PK/PD modelling, whereby effect is considered an unobserved normally distributed latent variable, and each item score of COMFORT-B is allowed to contribute to this through link functions.^46^

Mixed effects models will be fitted with maximum likelihood and addition of a single fixed effect will be guided by improvement of fit using the likelihood ratio test (change in objective function of 3.84 is significant at p=0.05 level for 1 df by the χ^2^ distribution). Model evaluation will consist of goodness-of-fit (residual plots) and simulation-based diagnostics (visual predictive check), and parameter precision and robustness will be investigated with non-parametric bootstrapping.

## Pharmacogenomic End points

Patients treated with sedatives and analgesics may respond very differently to the same dosage of medication. Several gene polymorphisms have been discovered to influence the PK and PD of clonidine, midazolam and morphine. PK data collected in this study can be interpreted more completely if data on pharmacogenomics are available.

Moreover, to study candidate polymorphisms gives further insight into the response of critically ill children to clonidine, midazolam and morphine. Most candidate genes have been established in adult subjects, this study is an opportunity to study the effects in children. The candidate genes which will be genotyped are shown in table 4.

**Table 4:** Candidate genes for linking pharmacogenomics to pharmacokinetic and pharmacodynamics end points

Midazolam	Clonidine	Morphine
CYP3A4, CYP3A5, POR, ABCB1, GABA, MDR1, MRP1, MRP2, MRP4, BRCP	ADRA2A, CYP2D6	COMT, OPRM1, OCT1, UGT2B7, ABCC3, MC1R, IL-1Ra, IL-1β, ARRB2, STAT6

## Ethical considerations

Ad hoc considerations and measures have been set up to ensure safe and ethical conduct of this multinational trial, including a population requiring special protection such as children. In order to guarantee the respect of ethical rules regardless of the country in which the trial is carried out, an ethical standard based on the EU ethical and legal framework has been agreed among centres. All submissions are based on the current European ethical and legal framework and on the international ethical principles and guidelines.

Based on the Council of Europe Convention on Human Rights and Biomedicine^47^ and EU Ethical Recommendations 2008,^48^ research involving vulnerable populations is allowed if the results of the research are expected to provide real and direct benefit to the health of the patient, or some benefit for the population represented by the patient, and the trial will pose only minimal risk and burden to the individual concerned. The risk-benefit assessment has been carefully addressed considering IMP-related risks (including pharmacological properties and proposed dosing regimen of the IMP, information on use in target population, IMP development, IMP management) and expected benefits, the trial design (including study population inclusion and exclusion criteria), study-related procedures, qualification of the study team and host sites, rights of patients (including informed consent and assent procedures and data protection and confidentiality). Minor increase over minimal risk is anticipated for the CLOSED trial, as the study deals with a novel treatment modality in vulnerable paediatric patients and carries minor risk based on the available information about the study drug as well as randomised controlled trial (RCT)-related procedures. Important benefits for PICU population as a group are anticipated, since the study will provide clinically relevant and directly applicable results, expected to influence future standard of care.

The informed consent and assent process in this trial is in line with the applicable relevant regulatory documents, including Good Clinical Practice (GCP) guideline,^49^ Directive 2001/20/EC,^50^ the EC Detailed Guidance 2006,^51^ Directive 95/46/EC,^52^ the International Ethical Guidelines for Biomedical Research Involving Human Subjects CIOMS-WHO (2002),^53^ the Additional Protocol to the Oviedo Convention (2005),^54^ ICH Topic E11 and the EC 2008 Paediatric Recommendations. Accordingly, clear and appropriate information sheets and informed consent forms for parents/legal representatives, and patient information sheets and assent forms are prepared for patients and customised according to the local requirements.

Given the nature of the trial, especially considering that ICU patients are often admitted as a result of emergency, deferred consent/assent, as described in Article 35 of the new European Regulation on clinical trials (EU) No 536/2014 of the European Parliament and of the Council,^36^ is foreseen if allowed by local legislation. Informed consent of the legal representative and, if feasible, the patient's assent will be sought for as soon as possible. If informed consent is not obtained, the possibility of withdrawing all collected data from the trial will be explained and appropriate measures, based on patient's decision will be taken. A record will remain in the subject's medical notes. In the Netherlands, deferred consent is permitted under national legislation (‘Wet medisch-wetenschappelijk onderzoek met mensen’, Paragraph 2 Article 6.4).

As the protocol includes pharmacogenetic tests, informed consent and assent to perform genetic tests will be obtained separately, in line with the most relevant provisions in the field (International Declaration on Human Genetic Data, UNESCO, 2003;^55^ Ethical Guidelines CIOMS-WHO, 2002). Accordingly, the participants will have the possibility to join the trial without participating in the pharmacogenetic part.

Finally, involvement of appropriate external expertise in the form of Data Safety Monitoring Board (DSMB), Independent Scientific Advisory Board (ISAB) and Patient Advisory Board (PAB) is foreseen. In details, the DSMB will review the study's safety data and assess subject safety data throughout the study; the ISAB will input in the final protocols, monitor the progress of the project and ensure the ongoing scientific and ethical integrity of the clinical trial; the PAB will advise the Sponsor to ensure that the subjects’ rights and subjects’ protection will outweigh any commercial considerations and conflicts of interest that may appear in relation to the project.

In accordance with GCP, as implemented at national level, the study protocol and related documents have been approved by the competent ethics committees in Rotterdam, the Netherlands, and in Erlangen, Germany. Submission is currently being prepared for the three other centres (Stockholm, Prague and Rome).

## Dissemination

The results will be published in a peer-reviewed journal and shared with the worldwide medical community.

## Discussion

### Study design

Sedation management in PICU mainly consists of the use of intravenous midazolam by continuous infusion. Midazolam has several advantages, but is by far not the ideal agent for longer-term sedation. Other agents have made their off-label entry into PICU, mainly based on adult practice. One of these agents is clonidine, which could have some specific advantages over midazolam. It is being used increasingly, but is still off-label. This study design allows for data collection on safety, efficacy, PK, pharmacogenomics and to a lesser extent neurological outcome. This multimodal approach generates data that is very useful for licensing clonidine for sedation in children. Moreover, application of different formulation strengths, allows for easier paediatric clinical implementation as the current available formulation (Catapresan) has only been developed for adults.

### Study limitations

Despite the careful design of this trial, some limitations exist. First, we have chosen a non-inferiority design for this trial because, based on clinical experience and limited available data, we estimate the difference in sedation success rate will be very low. This would mean that showing superiority of clonidine requires a large sample size and thus compromising feasibility. This design, however, means that we cannot demonstrate possible superiority of clonidine. However, if equal efficacy is shown under safe circumstances, clonidine can be licensed for sedation in PICU.

Second, the recruitment window has been set on 72 hours. This could lead to confounding as having a large time window could increase the risk of tolerance and withdrawal symptoms. A smaller recruitment window may reduce these risks but parents need some time to consider participation and not every study site has 24/7 research nurse availability.

Third, this trial has an extensive list of exclusion criteria. This could compromise the external validity of the results and could complicate implementation of the results in new guidelines. This is a known limitation of almost any controlled trial.^56^ However, these exclusion criteria are based on expected elements affecting both primary outcome and patient safety. Our primary end point is based on a validated behavioural scale. Therefore, any disease status that can cause many fluctuations in behaviour (such as neurological injury) needed to be excluded from this trial for the results to be valid. The same holds for safety end points such as cardiovascular stability. The decision to allow for inotropes/vasopressors in the second amendment is a big step towards generalisability of the results.

### Role of researcher-driven studies, Seventh Framework Programme-funded projects

CLOSED is one of the 20 projects approved in the framework of the Seventh Framework Programme for Research that should specifically meet both criteria for scientific excellence and regulatory standards for high-quality paediatric research, as prescribed by the Paediatric Regulation, in order to put paediatric drugs on the market. A very recent publication demonstrated that these projects face the need for overcoming the existing methodological and ethical difficulties affecting research in the paediatric population.^5^

In fact, paediatric clinical trials are often multinational and researchers need to know and apply rules from the regulatory, legal and ethical frameworks, acting both as investigators and as sponsor and/or other concerned parties. This is not simple considering the lack of harmonisation of clinical trial procedures among countries (http://www.ncbi.nlm.nih.gov/pubmed/26037896) and makes it necessary to prepare, agree and apply guidelines based on the EU rules.

### Challenge to perform a trial compliant with GCP and other regulations

Clinical trials must follow the same rules, notwithstanding if sponsored by industry or non-profit organisations, from GCP to the new clinical trials regulation^49^ to recommendations and guidelines.

Accordingly, the new regulation^49^ recognises that a large proportion of clinical trials are conducted by non-commercial sponsors, frequently supported by public funds or charities and that these trials should be encouraged.

However, this means a heavy work from researchers and a deep knowledge of both EU and national rules. This issue is aimed to be overcome by the international public-private cooperation between different stakeholders foreseen by EU-funded projects (researchers, clinicians, regulatory and ethical experts, clinical research associates). On the other hand, it seems that the paediatric Consortia generated by the FP7 paediatric projects are conducting these studies and trials using a limited amount of money in comparison with the recognised cost of paediatric trials in an approved PIP which is estimated to be three to four times higher.^5^

### Challenges of maintaining double-blinding

Clonidine and midazolam have different half-lives: 1–3 hours for midazolam (up to 12 hours in neonates) and 9–17 hours for clonidine, thus maintaining double-blinding is challenging. PK/PD simulations have played a major role in the strategy to overcome this challenge. It has led to the implementation of a loading dose, which is in line with the current clinical recommendations for midazolam.^44^ Target plasma levels have been achieved based on the literature.^32 43^ It should be noted, however, that the PK/PD relationship is assumed, but not formally studied. This study will contribute to the determination of a PK/PD relationship.

### Risk to patients

Both clonidine and midazolam are frequently used sedatives in PICU. However, a careful assessment of possible study-related risks has been performed. In general, side effects of IMP are the major cause of study-related risk. As midazolam is the gold standard treatment, the side effects do not contribute to an increased risk in this study. For clonidine, bradycardia and hypotension are the most clinically relevant side effects and are likely to occur most often during the loading doses and the early starting phase.^33^ Therefore, a loading dose will be given as a 15 min infusion instead of a direct bolus. Moreover, the setting where patients are being monitored consists of five well-equipped and experienced PICUs. Treatment of hypotension and/or bradycardia is common practice in the study population and should therefore not increase the risk to subjects dramatically.

Rebound hypertension could also be a side effect after sudden discontinuation of clonidine. In a comparable trial,^31^ this was observed once in 64 patients and resolved quickly without any intervention. Monitoring of blood pressure up to 72 hours after IMP cessation has been included in the study protocol with the recommendation to treat hypertension according to local common practice.

Blood sampling is a possible risk to small children participating in drug research.^57^ In this study, the amount of sampled blood will not exceed the 3% of patient's plasma volume, which is a general limit of study-related blood sampling.

In general, as most elements of this study are similar to current clinical practice, the study-related risk to patients is deemed low.

### Pharmacogenetics

This study incorporates a pharmacogenetics assessment of recruited patients. Both PK and PD may be influenced by gene polymorphisms. The current proposed pharmacogenetics assessment has been based on the literature available, but a degree of flexibility has been built in when more information on these genes or medications will come available.

In accordance with the appropriate regulations,^51 53 55^ separate consent and assent will be obtained from participants. This strategy enables the possibility of participating in the CLOSED trial without the genetic assessment.

### Anticipated difficulties

A comparable RCT, the Safety profiLe, Efficacy and Equivalence in Paediatric intensive care Sedation (SLEEPS) study, has been previously undertaken in the UK.^33^ One of the major challenges faced by the investigators was the recruitment. The sample size had been calculated on 1000 subjects. Unfortunately, only 129 subjects were randomised. Contributing factors to this impaired recruitment have been identified by the investigators, including:

- Conflict with other studies
- Earlier extubation in elective cardiac surgery patients
- Parental issues and timing of consent
- Clinicians’ issues
- Research nurse time
- Delay in study start
- Compliance with the protocol.

It is likely that in the participating centres with excellent research facilities, multiple trials are being performed at the same time. Children are not allowed to participate in multiple intervention studies, so we recommend to have a thorough look at the inclusion and exclusion criteria of studies and set up clear agreements about patient allocation.

Early extubation in cardiac surgery patients is now common practice, and estimations of recruitment rates have excluded these patients. However, it is likely that ventilation strategies will change over the coming years. We have recognised the increasing use of non-invasive ventilation such as Non-Invasive Positive Pressure Ventilation (NIPPV). As these patients may still need continuous sedation, we will include these in the study. In general, patients on continuous positive airway pressure (CPAP) do not need additional sedation, thus these will be excluded.

Parental issues are to be expected in any clinical trial in the PICU. Parents having their child admitted to an ICU are under high stress and may therefore be reluctant to participate. In the calculations of estimated recruitment, a high refusal rate (approximately 40%–50%) should be taken into account, based earlier experience with other trials. Also, a parent organisation is involved in the trial and they will provide information to parents from their perspective. We anticipated to include 300 patients within 18–24 months in total. In The Netherlands, deferred consent is used in emergency care trials. The Ethical Committee of the Erasmus Medical Centre, Rotterdam, has approved the use of deferred consent in this trial so this could be applied in the largest recruitment centre.

When faced with a critically ill child, doctors and nurses may tend towards conservatism. At the beginning of the SLEEPS trial, the involved professionals could have been afraid of the cardiovascular side effects of clonidine especially in unstable patients. Their study has shown that its use is relatively safe in critically ill children, as well as other literature.^34 58^ Moreover, in the CLOSED consortium recruitment centres, a lot of clinical experience with clonidine has been built so caregivers may be less hesitant to support this clinical trial.

Research nurse time is always an issue, especially in the 24/7 business of an ICU. In the larger centres, dedicated researchers/nurses have been identified and trained adequately to perform this trial. In the smaller centres, Principle Investigators (PI)s may be able to coordinate the lower amounts of participants.

During the first months of patient recruitment in two centres, no patients could be included. In the majority of cases, exclusion criteria made the screened patients ineligible. No consent was given in the very few eligible patients. Therefore, a revision of the exclusion criteria has been made.

The major exclusion criteria on which patients failed screening are:

- *Anticipated need for sedation <24 hours*. It has been considered to change this duration. It has been decided however to keep this minimum as the therapeutic aim of the study is long-term sedation.
- *No sedation despite ventilation.* To our surprise, many newborn patients on the ventilator would not need any sedation, or were comfortable on low-dose opioids. This could not be mitigated.
- *Circulatory insufficiency.* The Goldstein criteria^59^ have been used for the definition of this exclusion criterion. However, many patients received inotropic or vasopressor agents as supportive therapy in both recruiting centres. We have therefore modified these criteria and removed the use of inotropes/vasopressors in the second protocol amendment.
- *Neurological pathology.* It has been suggested to include patients with minor head trauma, but as the clinical picture may change very rapidly, we have decided not to include these patients into the trial.
- *Use of clonidine in the last 7 days.* As clonidine has a long half-life, patients would be excluded if they have received clonidine for any indication in the previous 7 days. However, for midazolam, there was no similar exclusion criterion; instead, patients would undergo an extra PK blood sample before start of the study medication. This has now been introduced for clonidine as well in the second protocol amendment.

A delay in study start has been encountered in the CLOSED study. Several obstacles, such as obtaining the appropriate licenses for handling, importing and exporting controlled drugs, have caused a significant delay in recruitment start. A second major issue has also been an amendment of the dose reduction scheme (see the ‘Study treatments’ section). A third issue has been the generation of robust quality data that is sensitive enough to support a future PUMA application as well as the Investigational Medicinal Product Dossier (IMPD) and clinical trial application.^60^

Compliance with the protocol was a significant issue in the SLEEPS trial, as they provided a strict sedation regimen. Therefore, the CLOSED sedation regimen merely reflects clinical practice, although some important aspects may cause difficulties in clinical practice. The lock-out periods, for example, are not according to clinical practice and special attention needs to be paid by the research nurses and other investigators to keep the protocol compliance as tight as possible. When problems are recognised, the possibility of amendments should be discussed early. Early recognition is facilitated by implemented monthly investigators’ teleconferences.

Also, there will be an ongoing evaluation of the COMFORT-B scale training of the nurses in the participating centres. Good interobserver reliability is defined by a Cohen's kappa >0.65 and nurses in all centres have received training by trained nurses.

In summary, many known challenges should be managed by appropriate measures taken. However, there could always be unknown challenges and it is therefore crucial to have regular contact between principal investigators of each recruitment site.

### Reflection

After 9 months of recruiting, we included far fewer children than anticipated. Therefore, two amendments have now been added to potentially increase the number of eligible patients. Unfortunately, recruitment did not improve. Even though we had been warned by the early discontinuation of the SLEEPS trial,^33^ we faced other challenges. For example, many postoperative neonates do not need any additional sedation to intravenous paracetamol and continuous intravenous morphine. Also, parents are reluctant to participate. Either they think their child is not stable enough, or their child is finally stable and therefore changes in treatment for study purposes are not welcomed.

Also, the anticipated recruitment rate was based on the number of admitted ventilated patients in previous years. However, this turned out to be a significant overestimation, a phenomenon also known as Lasagna's Law.^61 62^ These challenges have a big impact on the feasibility of the trial and force us to consider alternative options. We will open (at least) two new recruitment sites, Bari (Italy) and Tallinn (Estonia). Furthermore, we need to rethink the primary objective of this study and may change it to a PK/PD study for which 50 patients in each arm are sufficient instead of the original 150 patients per arm.

The lessons from both the SLEEPS trial and this trial are important for further investigations in paediatric critical care and careful preparation is warranted for any trial performed in this population. This preparation should at least include adequate piloting over a longer period. In our experience, we monitored eligible patients over one month in one centre and expected no significant recruitment problems. This monitoring was performed during winter time, when many patients with respiratory viral infections in need of mechanical ventilation were admitted. This caused our expectations of eligible patients to be high; if we had performed this during summer time, we may have been warned earlier.

Other aspects to enhance the number of included patients are adequate staff training and motivation, collaboration with investigators having experience in paediatric critical care trials and keeping the exclusion criteria of a trial to a minimum.

### Trial status

The trial started enrolling subjects in the summer of 2016 in Rotterdam and Erlangen, where ethical approval is available. To date, five patients are included. Recruitment in other sites will follow when ethical approval becomes available. The second amendment including changes of the exclusion criteria have been submitted to the ethical and regulatory institutions and approval has been obtained in Rotterdam and Erlangen. The current study protocol, including the second amendment, is V.3.0, dated 21 October 2016. In Rome, Prague and Stockholm, approval has only been given for protocol V.2.0, dated 22 February 2016. Approval for V.3.0 is expected in the first half of 2017.

## Supplementary Material

Reviewer comments

Author's manuscript
