# Correction: The CLOSED trial; CLOnidine compared with midazolam for SEDation of paediatric patients in the intensive care unit: study protocol for a multicentre randomised controlled trial

**DOI:** 10.1136/bmjopen-2017-016031corr1

**Published:** 2017-11-08

Neubert A, Baarslag MA, Dijk MV CLOSED Consortium*, et al*. The CLOSED trial; CLOnidine compared with midazolam for SEDation of paediatric patients in the intensive care unit: study protocol for a multicentre randomised controlled trial. *BMJ Open* 2017;**7**:e016031. doi: 10.1136/bmjopen-2017-016031.

The names of the members on the Consortium were missed from the article. The full list of members is attached, supplementary file 1.

10.1136/bmjopen-2017-016031corr1.supp1 Supplementary file 1

