# Supplementary material for: The CLOSED trial; CLOnidine compared with midazolam for SEDation of paediatric patients in the intensive care unit: study protocol for a multicentre randomised controlled trial
Source: BMJ Open. 2017 Jun 21;7(6):e016031. doi: 10.1136/bmjopen-2017-016031 (PMC5726127; doi:10.1136/bmjopen-2017-016031)
Supplement: Author's manuscript [file bmjopen-2017-016031.draft_revisions.pdf]

The CLOSED- trial; CLOnidine compared to midazolam for SEDation of paediatric patients in the intensive care unit: study protocol for a multicentre randomised controlled trial

Journal:	*BMJ Open*
Manuscript ID	bmjopen-2017-016031
Article Type:	Protocol
Date Submitted by the Author:	19-Jan-2017
Complete List of Authors:	Neubert, Antje; Friedrich-Alexander-University Erlangen/Nuremberg, Loschgestr. 15, 91054, Erlangen, Germany<br>Baarslag, Manuel; Erasmus MC-Sophia Children's Hospital, Intensive Care and Pediatric Surgery<br>van Dijk , Monique; Erasmus MC Sophia Childrens Hosp<br>van Rosmalen, Joost; Erasmus MC, Biostatistics<br>Standing, Joseph; Institute of Child Health, Infectious Diseases and Microbiology Unit<br>Sheng, Yucheng; University College London, Institute of Child Health<br>Roberts, Deborah ; Therakind Ltd<br>Winslade, Jackie; Therakind Ltd<br>Rawcliffe, Louise; Therakind Ltd<br>Hanning, Sara; University College London, UCL School of Pharmacy<br>Metsvaht, Tuuli; Tartu Ulikooli Kliinikum, Clinic of Anaesthesiology and Intensive Care<br>Giannuzzi, Viviana; Fondazione per la Ricerca Farmacologica Gianni Benzi ONLUS<br>Larsson, Peter; Astrid Lindgrens Barnsjukhus, Pediatric Anesthesia and Intensive Care<br>Pokorná, Pavla; Univerzita Karlova v Praze 1 lekarska fakulta, Department of Pediatrics<br>Simonetti, Alessandra; Ospedale Pediatrico Bambino Gesu, Clinical Trial Center<br>Tibboel, Dick; Erasmus MC-Sophia Children's Hospital, Intensive Care and Pediatric Surgery
**Primary Subject Heading**:	Intensive care
Secondary Subject Heading:	Paediatrics
Keywords:	Paediatric intensive & critical care < ANAESTHETICS, CLINICAL PHARMACOLOGY, Paediatric anaesthesia < ANAESTHETICS, PAEDIATRICS

For peer review only

The CLOSED- trial; CLOnidine compared to midazolam for SEDation of paediatric patients in the intensive care unit: study protocol for a multicentre randomised controlled trial

List of authors:

Antje Neubert[1], Manuel A Baarslag*[2], Monique van Dijk[2], Joost van Rosmalen[3], Joseph F Standing[4], Yucheng Sheng[4], Deborah Roberts[5], Jackie Winslade[5], Louise Rawcliffe[5], Sara M Hanning[6], Tuuli Metsvaht[7], Viviana Giannuzzi[8], Peter Larsson[9], Pavla Pokorná[10], Alessandra Simonetti[11] and Dick Tibboel[2]; On behalf of the CLOSED Consortium

***Corresponding Author:**

Manuel Alberto Baarslag

Room Sp-2430

Wytemaweg 80

3015 CN Rotterdam

The Netherlands

For peer review only

Telephone: +31107030380

E-mail: m.baarslag@erasmusmc.nl

Author affiliations

1. Department of Children and Adolescents Medicine, Friedrich-Alexander-University Erlangen/Nuremberg, Erlangen, Germany
2. Intensive Care and Department of Pediatric Surgery, Erasmus MC-Sophia Children's Hospital, Rotterdam, The Netherlands
3. Department of Biostatistics, Erasmus MC, Rotterdam, The Netherlands
4. Institute of Child Health, University College London, London, United Kingdom
5. Therakind Ltd, London, United Kingdom
6. UCL School of Pharmacy, University College London, London, United Kingdom
7. Clinic of Anaesthesiology and Intensive Care, Tartu University Hospital, Tartu, Estonia
8. Fondazione per la Ricerca Farmacologica Gianni Benzi onlus, Valenzano, Italy
9. Department of Pediatric Anesthesia and Intensive Care, Astrid Lindgrens Childrens Hospital, Karolinska University Hospital, Stockholm, Sweden
10. Department of Pediatrics, First Faculty of Medicine, Charles University Prague, Czech Republic
11. Clinical Trial Center, Bambino Gesù Children's Hospital, Rome, Italy

Trial registration: EudraCT: 2014-003582-24; Clinicaltrials.gov: NCT02509273

Key words: Sedation, children, critical illness, paediatrics, pharmacology

Word count: 6630

For peer review only

List of abbreviations

BIS: Bispectral Index Monitoring; CNS: Central nervous system; CPAP: Continuous Positive Airway Pressure; DSMB: Data Safety Monitoring Board; ECMO: Extracorporeal Membrane Oxygenation; GA: Gestational Age; GABA: Gamma-Amino Butyric Acid; IMP: Investigational Medical Product; ISAB: Independent Scientific Advisory Board; IV: Intravenously; NIPPV: Non-invasive Positive Pressure Ventilation; NMDA: N-methyl-D-aspartate; NIMP: Non-Investigational Medical Product; NISS: Nurses' Interpretation of Sedation Score; NRS: Numeric Rating Scale; PIP: Paediatric Investigation Plan ; PICU: Paediatric Intensive Care Unit; PD: Pharmacodynamics; PK: Pharmacokinetics; SmPC: Summary of Product Characteristics; PUMA; Paediatric-Use Marketing Authorisation

Abstract

Introduction:

Sedation is an essential part of paediatric critical care. Midazolam, often in combination with opioids, is the current golden standard drug. However, as it is a far-from-ideal agent, clonidine is increasingly being used in children. This drug is prescribed off-label for this indication, as many drugs in paediatrics are. Therefore, the CloSed trial aims to provide data on the pharmacokinetics, safety and efficacy of clonidine for the sedation of mechanically ventilated patients in order to obtain a paediatric-use marketing authorisation (PUMA).

Methods and analysis:

The CloSed study is a multi-centre, double-blind, randomised, active-controlled non-inferiority trial with a 1:1 randomisation between clonidine and midazolam. Both treatment groups are stratified according to age in three groups with the same size: <28 days (n=100), 28 days to <2 years (n=100)

For peer review only

and 2-18 years (n=100). The primary endpoint is defined as the occurrence of sedation failure within the study period. Secondary endpoints include a PK/PD relationship, pharmacogenetics, occurrence of delirium and withdrawal syndrome, opioid consumption and neurodevelopment in the neonatal age group. Logistic regression will be used for the primary endpoint, appropriate statistics will be used for the secondary endpoints.

Ethics:

Written informed consent will be obtained from the parents/caregivers. Verbal or deferred consent will be used in the sites where national legislation allows. The study has IRB approval at recruiting sites. The results will be published in a peer-reviewed journal and shared with the worldwide medical community.

Registration:

EudraCT: 2014-003582-24; Clinicaltrials.gov: NCT02509273

Strengths and limitations of this study

- The dosing scheme being used in this trial is based on detailed PK-PD modelling data
- Pain and sedation are measured using a standardised assessment scheme (COMFORT-B Score) across all participating units
- Besides information on the efficacy and safety of clonidine in critically ill children, this trial will provide population-based PK information in this specific patient population
- Age-specific formulations are being used in this trial, which can be marketed afterwards
- A previous similar trial indicated challenging recruitment, this could be a potential thread to this trial

For peer review only

Introduction

Unlicensed and off-label drug use

In Europe, less than 30% of marketed drugs include results from paediatric clinical trials and other information on paediatric use in their documentation (Summary of Product Characteristics, SPC, or Product Leaflet)[1]. This results in widespread off-label use in paediatrics, especially in the case of old drugs that have never received a paediatric authorisation. Off-label paediatric use (including all the uses not listed in the SmPC[2]) in Europe accounts for 45-60% of the total number of prescriptions, with rates of up to 90% in patients admitted to neonatal or paediatric intensive care units[3].

The entry into force of the Paediatric Regulation in 2007[4] gave an important stimulus to support the development of medicines for children by introducing a specific measure to favour work on off-patent medicines, the so-called Paediatric Use Marketing Authorisation (PUMA). A PUMA application should include the submission of paediatric data in accordance with an agreed Paediatric Investigation Plan (PIP).

According to article 40 of the Regulation, the European Commission (EC) reserved funds 'to develop off-patent medicinal products with recognised therapeutic interest for children and included in a 'Priority List' adopted by the EMA (European Medicines Agency) through its Paediatric Committee (Seventh Framework Programme for Research HEALTH-(2007-2013) Programme area - topic 4.2-1). Among 20 projects approved[5], the CLOSED project was granted with the aim to develop an age-appropriate formulation of clonidine for sedation in PICU, in line with the EMA "Revised Priority List for Studies into Off-Patent Paediatric Medicinal Products" 2012[6]. In order to specifically meet this therapeutic need, a comprehensive development plan in the form of a Paediatric Investigation Plan (PIP) was submitted to the EMA Paediatric Committee (PDCO) in July 2012 and approved in January 2013. In March 2015, a modification of the PIP was proposed after

For peer review only

the finalisation of the clinical study protocol. This modification was approved by the PDCO in August 2015.

Analgosedation in the PICU

Approximately 2% of all paediatric patients admitted to hospital require treatment in the Paediatric Intensive Care Unit (PICU)[7]. Most PICU admissions are unplanned emergencies, mainly in the context of congenital heart diseases (40%), respiratory diseases (20%), major trauma (15%) and neurological problems other than trauma (<10%)[8].

Often, mechanical ventilation is required for facilitating recovery after major surgery or for treating respiratory failure. In most of these cases, analgosedation using potent opioids and sedatives is mandatory to reduce metabolic rate and oxygen demand, assist mechanical ventilation, avoid inadvertent self-extubation and lower anxiety and distress[9, 10]. To this effect, a variety of agents are used in the NICU and PICU, such as opioids, GABA-receptor agonists, NMDA-receptor antagonists and α2-receptor agonists[11].

Opioids

The main opioids used in PICU and NICU are morphine, fentanyl and remifentanil. Opioids bind to the μ receptor in the central nervous system (CNS), and provide analgesia as well as sedation.

GABA-receptor agonists

GABA receptors have an inhibitory effect on the CNS. Benzodiazepines agonise these receptors and therefore have sedative, anxiolytic and anticonvulsant properties. Disadvantages are paradoxal reactions (agitation and confusion) and tolerance and withdrawal after prolonged use.

Propofol is a unique agent with GABA-ergic properties as well as anti-NMDA and sodium channel blocking effects. Propofol is not suitable for long-term sedation, due to the perceived risk of propofol infusion syndrome, a metabolic derangement accompanied by severe metabolic acidosis,

For peer review only

hyperkalemia, hyperlipidemia, rhabdomyolysis and organ failure, associated with an increased risk of mortality[12].

Midazolam

Midazolam is the most widely used sedative agent in the PICU. It is a short-acting benzodiazepine (Tmax of 5-10 minutes after IV injection) with a half-life of 1-3 hours (up to 12 hours in neonates). Besides sedation and anxiolysis, midazolam also provides anterograde amnesia, thus minimizing children's recall of unpleasant experiences after a PICU admission.[13] Midazolam is mainly metabolised to the equipotent metabolite 1-OH-midazolam, and then glucuronidated to the renally excreted 1-OH-MDZ-glucuronide. Its main adverse effects include tolerance, dependence and withdrawal syndrome following discontinuation.

Clonidine

Clonidine agonises the α2-adrenergic receptor. The reduced sympathetic outflow[14] in the CNS results in sedation, anxiolysis and analgesia[15, 16]. Because of its analgesic properties, it has been used as an adjunct in surgical procedures as premedication or as a supplementary agent in regional anaesthesia[17]. The reduction of sympathetic outflow associated with clonidine may have specific benefits in critically ill children. Alpha-2 agonists can improve neurological outcome associated with ischemic cerebral injury[18-20], the mechanism of action of which is unclear but may be due to suppression of extracellular glutamate and aspartate release during energy failure[21]. Recent data have also demonstrated that pre-conditioning before the insult can both reduce infarct size and improve neurological outcome after insult[22]. Surgery and critical illness are associated with a variety of stress responses, which can result in organ dysfunction[23]: Renal function deteriorates after both adult and paediatric cardiac surgery, and this effect is due in part to the increase in sympathetic outflow and the rise in circulating vasoconstrictors such as noradrenaline, vasopressin and angiotensin[24, 25]. Clonidine has been demonstrated to suppress these responses and prevent

For peer review only

the associated decline in renal function after adult cardiac surgery[26]. In addition, clonidine has independent local effects on tubular function which promote both diuresis and natriuresis[27]. In terms of cardiovascular responses, reduction in stress responses by alpha-2 agonists have been shown to reduce perioperative myocardial ischemia in adults undergoing cardiac and non-cardiac surgery[28]. As the mechanism of action of clonidine is very different from the GABA-agonist midazolam, the hypothesis is postulated that it may be less associated with paediatric delirium and withdrawal syndrome. Moreover, clonidine is one of the agents used for the treatment of withdrawal syndrome[29].

Previous clinical trials

Clonidine is commonly used off-label in paediatric anaesthesia and intensive care medicine. Its use is recommended for the sedation of critically ill children in PICUs by the UK and German consensus guidelines[12, 30] and local hospital guidelines across Europe. Unfortunately, despite this widespread use of clonidine there are limited data on efficacy, dose requirement and safety when used for sedation on PICUs. A number of studies have been performed[31-35], however, to overcome this knowledge gap. See table 1 for a short overview.

Table 1. An overview of paediatric studies involving clonidine for sedation in the intensive care unit.

**Study**	**Sample size and age**	**Design**	**Outcome**
**Ambrose et al. [31]**	N=30, 0-10 years	Three-step: IV Low-dose vs. high-dose (variable dose together with midazolam), 3rd group fixed dose	No adverse effects on haemodynamics, sufficient sedation in combination with midazolam
**Arenas-Lopez et al. [32]**	N=24, 0-5 years	Prospective cohort study, oral clonidine as additive to morphine/lorazepam	Opioid- and benzodiazepine sparing, safe and effective
**Wolf et al. [33]**	N=129, 0-15 years	Double-blind, randomised controlled	No difference in effectivity, underpowered

For peer review only

		trial of IV clonidine vs. midazolam	due to recruitment problems
**Hünseler et al. [34]**	N=219, 0-2 years	Double-blind, randomised controlled trial of IV clonidine vs. midazolam	Opioid- and benzodiazepine-sparing in neonatal age group
**Duffett et al. [35]**	N=50, 0-18 years	Double-blind, randomised controlled trial of oral clonidine vs. placebo in addition to physician-driven sedation	No significant difference in effectivity, study with clonidine clinically feasible

In summary, when used in addition to standard sedation management, clonidine has a benzodiazepine- and opioid-sparing effect. Comparative studies showed equivalence, but were either underpowered[33] or set up as a pilot trial[35].

Study aims

For the purpose of sedation of intubated and mechanically ventilated paediatric patients, this clinical study sets out to provide the data needed for a Paediatric Use Marketing Authorisation (PUMA) application for the use of clonidine in PICU patients:

- data on efficacy and safety of clonidine compared to midazolam.
- PK data on both clonidine and midazolam in critically ill children and adolescents, using a population-based approach.

Together with the development of age- and weight-adapted formulations of clonidine, as part of this study, data on quality and stability of these new formulations will also be generated.

Methods/design

Study design

The CloSed study is a multi-centre, double-blind, randomised, active-controlled non-inferiority trial with a 1:1 randomisation between clonidine and midazolam. Both treatment groups are stratified according to age in three groups with the same size: <28 days (n=100), 28 days to <2 years (n=100)

For peer review only

and 2-18 years (n=100). The primary endpoint is defined as sedation failure within the study period.

Patient recruitment

Patients in five different EU member states (Czech Republic, Germany, Italy, The Netherlands and Sweden) will be recruited. Patients (neonates >34 gestational weeks and children <18 years) eligible for inclusion in the trial, according to in- and exclusion criteria, will be screened for possible enrolment. Operating lists will be screened on a daily basis for possible patients expected to be admitted to the intensive care unit. These patients and parents will be identified, approached, informed and enrolled prior to surgery by study responsible doctors and/or nurses. Patients eligible for inclusion transferred to the centres will be identified by the transport team. Patients admitted to the PICUs will continuously be screened by PICU medical staff to identify patients possible for enrolment. Parents of these eligible patients will be approached, informed and enrolled by study responsible doctors and/or nurses.

Children are often admitted to the PICU/ NICU in an emergency setting. Urgent action will most likely be required and the subject's parent(s)/ legal guardian(s) may not be immediately available to give consent. An example of this is when a critically ill baby is delivered by Caesarean section; the mother may still be under anaesthesia while another parent/ legal guardian of the child may not be present. Therefore, given the nature of the trial, especially considering that ICU patients are often admitted as a result of emergency, deferred consent/assent, as described in Article 35 of the new European Regulation on clinical trials (EU) No 536/2014 of the European Parliament and of the Council[36], is foreseen if allowed by local legislation, as described in section "Ethical considerations". This allows for the child to be enrolled in the trial before the parent(s)/ legal guardian(s) are able to give consent, which is then sought once they are available. If, subsequently, deferred consent is withheld, the subject is removed from the study but a record will remain in the subject's medical notes.

For peer review only

Study treatments

Firstly, it was necessary to define the dosing scheme so that appropriate drug concentrations could be used to maintain double blinding. A plasma clonidine concentration of around 2 mcg/L gave adequate sedation combined with morphine infusion in ventilated PICU patients[32], and 2 mcg/L clonidine concentration also drops the Bispectral Index to 71 in adults[37]. Clonidine has a long elimination half-life (16.9 h in neonates, 11.4 h in infants and 7.4 h in children[38] so loading doses are proposed. It was therefore decided that loading and maintenance doses aimed at achieving steady-state concentrations of around 2 mcg/L would be aimed for, and this scheme was designed by a PKPD expert working group within the CLOSED consortium. Based on these doses, a similar dose scheme of midazolam was designed that would maintain double blinding.

The agreed dosing scheme consists of 15-minute loading doses followed by a maintenance infusion rate. Increases or decreases, based on the evaluation of COMFORT-B and NISS score, in infusion rate are permitted, and preceding any increase a loading dose over 15 minutes is specified. There are also compulsory lockout times between each dose escalation or de-escalation to allow new steady-state conditions to be approached. Participants will follow the dosing regimen, with 1 unit meaning 1 microgram for clonidine, or 100 micrograms for midazolam:

- 1st bolus (T=0). Initial loading dose is 2 units/kg over 15 minutes and the following continuous infusion 1 unit/kg/h will be administrated to all patients followed by 15 minutes lockout period.
- 2nd bolus. If insufficient sedation after the 1st lockout period, the second loading dose is 2 units/kg over 15 minutes and the following continuous infusion 1 unit/kg/h will be administrated with a 15 minutes lockout period.
- 3rd bolus. If insufficient sedation after the 2st lockout period, the third loading dose is 2 units/kg over 15 minutes and the following continuous infusion 1.5 unit/kg/h will be administered with a minimum of 105 minutes lockout period.

For peer review only

• 4th bolus. If insufficient sedation after the 3rd lockout period, the fourth loading dose is 2 units/kg over 15 minutes and the following continuous infusion 2 unit/kg/h will be administered followed by a105 minutes lockout period.

• 5th bolus. If insufficient sedation after the 4th lockout period, the fourth loading dose is 2 units/kg over 15 minutes and the following continuous infusion 2 unit/kg/h will be administered with a 24 hours lockout period.

• 6th to 11th bolus. If insufficient sedation after 24h lockout period, the sixth to eleventh loading dose of 2 units/kg over 15 minutes and the following continuous infusion 2 unit/kg/h would be administered with at least 24 hours lockout period between.

All doses are halved in patients younger than 1 month. Figure 1 shows the scenario of all loading doses of study drug administered for subjects.

[Insert Figure 1 here].

Decreasing the maintenance dose will be possible in the case of over-sedation (i.e. COMFORT-B>22 OR COMFORT-B=11-22 and NISS=3).

Dose decrease will take the following steps (doses are halved in neonates):

- 2 units/kg/h will be decreased to 1.5 units/kg/h
- 1.5 units/kg/h will be decreased to 1 units/kg/h
- 1 units/kg/h will be decreased to 0.75 units/kg/h
- 0.75 units/kg/h will be decreased to 0.5 units/kg/h

If patients are still over-sedated with the minimal dose, the IMP administration will temporarily be stopped until patients need sedation again, and they will start back at T=0.

During the preparation of the trial, one unknown challenge has faced the consortium already. In version 1.0 of the protocol, dose reduction should be performed when a child was over-sedated. After 30 minutes, a new sedation assessment should be performed and acted upon the same. In an

For peer review only

over-sedated child, this could lead to a child being without IMP after 90 minutes (3 dose reductions). This is certainly not a desirable situation, but not unthinkable as the half-life of both drugs are longer than 90 minutes. Therefore, a six-hour observation period has been implemented. After a dose reduction, it is recommended to wait for 6 hours before the next dose reduction.

In this same amendment, another problem has been mitigated. Version 1.0 did not provide clear information about increasing the dose again after a prior decrease. In the amendment, it has been made clear that subjects having a decrease of maintenance infusion to <1 unit/kg/hr, will return to 1 unit/kg/hr, irrespective of the current infusion rate. If the decrease has been made while increases before have led to a maintenance infusion rate of >1 unit/kg/hr, re-increase will be following the precedent step (for example: if a decrease has taken place from 1.5 to 1 unit/kg/hr, re-increasing will return the rate back to 1.5 unit/kg/hr).

Pharmaceutical development

To overcome the potential for administration errors associated with commercially available clonidine ampoules, three different strengths of clonidine HCl – low (250 mcg/50ml), medium (500 mcg/50ml) and high (2500 mcg/50ml) have been developed. Based on the dosing regimen described above, the concentration of the low, medium and high strengths of midazolam (25 mg/50ml, 50 mg/50ml and 250 mg/50ml) were 100-fold higher than for clonidine HCl. For study blinding purposes and to avoid dosing errors, a simple 3 colour scheme based on strength will be used.

To avoid the administration of preservatives, all formulations will be stored in single use glass vials, with any contents remaining after 24 hours to be discarded. A 50ml volume has been selected to limit the number of children requiring more than one vial in 24 hours (only subjects weighing over 47kg and receiving the maximum dose in 24 hours will require two vials in a 24-hour period).

Efficacy endpoints

Level of sedation will be evaluated using the COMFORT-B score (ranging from 6 to 30) which is a validated scoring system commonly used in the PICU[39]. Level of sedation will also be evaluated by the Nurses Interpretation of Sedation Score (NISS) when the COMFORT-B score falls between ≥11and ≤22. The NISS is the nurse's expert opinion of the level of sedation (1: insufficient sedation; 2: adequate sedation; 3: over-sedation). A subject is considered to be under-sedated in cases of COMFORT-B score of >22; or COMFORT- B score of 11–22 in combination with NISS of 1.

In addition to COMFORT-B Score (and NISS where appropriate), pain will be assessed using the Numerical Rating Scale (NRS) at the same time intervals. The 11-point NRS is a global pain rating scale with which the nurse rates pain intensity by number (0 = no pain and 10 = worst imaginable pain)[40]. In this study, to simplify matters, the NRS score will be addressed first, so, if the pain score is ≥ 4, morphine treatment will be escalated whilst sedation treatment will remain the same, regardless of the COMFORT-B score. This means that any pain score will take precedence over the sedation score.

Primary objective

To assess the non-inferiority of the sedative properties of continuous IV clonidine compared to continuous IV midazolam in mechanically ventilated children and adolescents (0-18 years) admitted to a PICU.

Primary Endpoint

The primary endpoint is defined as sedation failure within the study treatment period (a maximum of seven days).

Sedation failure is defined as:

When a subject's assessment results are:

Numerical Rating Scale (NRS) score <4 and COMFORT B score >22

For peer review only

OR

Numerical Rating Scale (NRS) score <4 and COMFORT B score ≥11and ≤22 AND Nurses' Interpretation of Sedation (NISS) score 1 at a point during the study where no further increase in IMP dose are permitted as described in the dose escalation scheme.

In summary there are two possible outcomes (success or failure) by which the primary endpoint is measured.

Secondary objectives

To evaluate the safety and tolerability (including withdrawal effects) of clonidine compared with midazolam in ventilated children and adolescents admitted to PICU.

To determine clonidine dose-dependent effects on sedation.

To establish the PK-PD relationship of clonidine for sedation in PICU.

To compare the cumulative total morphine consumption/kg between the two arms in the first 48 hours of IMP administration.

Secondary endpoints

Primary PK parameters estimated will be clearance (CL), volume of distribution (VD) and inter-compartmental clearance (Q). PK measurements will be made using sparse opportunistic sampling.

PK-PD modelling will seek to elucidate the relationship between IMP pharmacokinetics and sedation as measured by COMFORT-B score.

The PK-PD covariate model will include demographics (e.g. age, weight), clinical characteristics (e.g. reason for admission) and pharmacogenomics (see Genetic parameters section).

Safety and tolerability assessments

Safety and tolerability assessments include:

For peer review only

- Extent of withdrawal effects using the Sophia Observation withdrawal Symptoms-Paediatric Delirium (SOS-PD) scale measured three times a day in subjects who receive sedatives and/or opioids for 5 days or more and after cessation of treatment in all subjects for at least 24 hours after treatment. The extent of delirium measured by the Sophia Observation withdrawal Symptoms- Paediatric Delirium (SOS-PD) scale[41].
- Rebound hypertension monitored for at least 72 hours post cessation of treatment.
- Percentage of respiratory depression per group.
- Adverse event reporting of symptoms indicative of post-ICU stress (e.g. nightmares, confusion, hallucinations).
- Neurodevelopment of subjects recruited in lower age group (from birth to 27 days) at 12 months after cessation of IMP, as measured using the Bayley II Score[42].

Effect size justification

The primary objective of this study is to evaluate the efficacy of clonidine compared to midazolam in the sedation of ventilated children and adolescents admitted to PICU, with a view to demonstrating non-inferiority of clonidine.

Every patient enrolled will be in need of sedation, thus a placebo cannot be considered. Intravenous midazolam is the standard treatment for longer-term sedation in children. It is licensed in this population, regarded as standard of care, and recommended by treatment guidelines[12, 30].

Based on clinical experience and the limited data available regarding the use of clonidine as a sedative agent in the PICU, it is assumed that the sedation success rate for clonidine is higher than for midazolam. The estimated difference in success rate between the two drugs was assumed to be 5%.

For peer review only

The primary endpoint is the success or failure of the subject's sedation treatment. Failure is defined as inadequate sedation with no further dose increases allowed in the dose titration scheme. This will be assessed by a nurse or physician using the COMFORT-B sedation assessment and, if appropriate, Nurse's Interpretation of Sedation Score (NISS). These assessment scales are open to a degree of subjectivity and it is therefore considered essential to have a double-blind study design to minimise bias due to knowledge of treatment group by the assessor.

In order to define the effect size the expected success rate for midazolam was needed. The target concentration for midazolam was taken from de Wildt et al 2005 paper showing around 311 mcg/L is required for sedation[43]. The maximum midazolam dose of 200 (100-200) mcg/kg bolus followed by 200 (50-200) mcg/kg/h for all age groups (including neonates) is recommended by Dutch clinical consensus guideline [44]. According to the proposed dosing schedule, the exposure of midazolam is similar to the Dutch recommended dosing regimen from the simulation (Figure 2). Furthermore, after the 5th bolus according to the proposed dose scheme, the proportion of average midazolam plasma concentration above the target value (0.311 mg/l) is 74% and from Dutch dose scheme is 72%. Thus, we assumed that the sedation success rate in the midazolam arm given our dosing scheme would be 75%.

[Insert figure 2 here]

Sample size calculation

Given this assumed effect size of a 75% success rate for midazolam, a sample size of 258 (129 per treatment arm) provides at least 80% power to show non-inferiority of clonidine compared to midazolam. The sample size calculation is based on a logistic regression for the primary efficacy endpoint of sedation success, with adjustment for study arm, age category, and baseline COMFORT-B score. Non-inferiority of clonidine requires that the lower limit of the one-sided 97.5% confidence

For peer review only

interval for the odds ratio of sedation success using clonidine (versus midazolam) is at least 0.583, which is equivalent to a 10% non-inferiority margin in case of a sedation success rate of 80%. The required sample size was calculated using simulation.

It is assumed that the total drop-out rate will be less than 13% and hence 300 subjects will be recruited, 150 in each arm and 100 in each age subgroup. The sample size of 300 subjects also provides at least 70% power to prove non-inferiority with respect to sedation success rate for each age subgroup (>90 subjects per age subgroup). Since the sample size for the age subgroup analyses is limited, a 15% non-inferiority margin at a one-sided alpha level of 0.025 is used, with an assumed sedation success rate of 80% in the midazolam group and 85% in the clonidine group. Subgroup analyses will be performed using a confidence interval for the Mantel-Haenszel risk difference, with stratification by centre.

Statistical analysis

Analysis Populations

The Safety Evaluation Set (SES) is the subset of all subjects who were randomised into the trial and exposed to study medication (also referred to as the Intention To Treat (or ITT) population). Safety analyses will be performed in the SES.

The Full Analysis Set (FAS) is the subset of subjects in the SES for whom the primary efficacy variable is available. Data analysis for the efficacy endpoints will be performed in the FAS.

The Per Protocol Set (PPS) is the subset of subjects in the FAS without major protocol deviations. Major protocol deviations will be defined during the Blinded Data Review Meeting (BDRM).

The Pharmacokinetic/Pharmacodynamic Analysis Set (PKS) is the subset of subjects in the SES with evaluable PK samples defined as drug concentration measurements.

For peer review only

Primary Efficacy Analysis

Logistic regression of the primary endpoint, sedation failure, with treatment group, baseline sedation assessment (i.e. the baseline COMFORT-B score), centre and age group as covariates, at a one-sided significance level of alpha = 2.5% will be used. The statistical hypotheses are the following:

H0: OR<= δ_{OR} and H1: OR > δ_{OR} ,

$OR=p_C*(1-p_M)/((1-p_C)*p_M)$

where p_C and p_M are probabilities of sedation success in the clonidine group and midazolam group, respectively. δ_{OR} is the non-inferiority margin which is predefined as 0.583.

H0 will be rejected if the lower bound of the one-sided 97.5% Wald confidence interval (CI) for the odds ratio of sedation success proportion will be greater than 0.583. The primary analysis will be based on the ITT principle. The primary endpoint will be evaluated for the Full Analysis Set. An equivalent analysis for the per protocol set will be performed as a sensitive analysis. No interim analyses are planned for the primary endpoint

Secondary Efficacy Analyses

All secondary efficacy analyses will be conducted on the Full Analysis Set. Secondary efficacy endpoints will be analysed in detail as follows:

- For the proportion of subjects with sedation success between treatments within each age group will be based on the confidence interval for the Mantel–Haenszel risk difference) with stratification by centre.[45] A 15% non-inferiority margin at a one-sided alpha level of 0.025 will be used.

For peer review only

- The aggregate intervals (based on the planned assessment points) with COMFORT-B Score >22 or <11 during treatment period between treatment groups will be analysed using the Mann-Whitney test.
- The proportion of subjects with COMFORT-B Score ≤11 between treatment groups and within each age group will be analysed using the chi-square test or Fisher's exact test.
- For the morphine consumption in units of mcg/kg/day during the first 24 hours of IMP administration and for the remaining study periodbetween treatment groups will be analysed using linear regression analysis, with adjustment for centre and age group. An appropriate transformation of morphine consumption may be used to ensure normality of the residuals in the linear regression model.

An age subgroup analysis will be performed for each age group, using centre as a stratification variable.

Pharmacokinetic/pharmacodynamic analyses

The primary objective of pharmacokinetic/pharmacodynamic analyses in this trial is to evaluate the pattern and extent of covariates affecting the PK/PD profiles of clonidine and to provide the information of optimal dose for patients based on their particular age and other related covariates for future clinical practice. Three PK-PD interim analyses will be undertaken whereby samples will be shipped and assayed for drug concentrations in order to ensure the systems for sample transport and analysis, along with data linkage to the eCRF are fully operational, and to allow preliminary PK-PD model development. PK-PD interim analyses will be conducted after 15, 100 and 200 subjects completed the study.

PK/PD data will be modelled using the Fortran-based non-linear mixed effects software NONMEM. Typical PK dispositional compartmental models will be tested, along with investigation linear and

For peer review only

non-linear elimination. Multivariate covariate analysis will be undertaken to investigate the impact of subject factors on PK model parameters. In particular we will focus on the effect of body size with allometric models, and age (both postmenstrual and postnatal) possibly using literature prior models in order to delineate the effect of size and age from other factors. We will also look at the impact of drug metabolising enzyme genotype on inter-individual variability. The link between PK and PD (COMFORT-B score) will be investigated by sequential and simultaneous modelling, possibly including an effect compartment. The Item Response Theory (IRT) will also be considered for PK-PD modelling, whereby effect is considered an unobserved normally distributed latent variable, and each item score of COMFORT-B is allowed to contribute to this through link functions[46]. Since subjects will receive other sedative agents for procedures, the concentrations of which may not be measured, a PK-PD approach will be taken to model sedation requirements in this period.

Mixed effects models will be fitted with maximum likelihood and addition of a single fixed effect will be guided by improvement of fit using the likelihood ratio test. Model evaluation will consist of goodness-of-fit (residual plots) and simulation-based diagnostics (visual predictive check), and parameter precision and robustness will be investigated with non-parametric bootstrapping.

Safety Analyses

All safety analyses will be performed on the SES. Analyses will include the following endpoints:

- AEs, SAEs and SUSARs.
- Deaths
- Clinical laboratory evaluations and vital signs. Descriptive summaries of laboratory values (including clinical chemistry, haematology, coagulation and urinalysis variables) and changes from baseline throughout the study will be generated. Shift tables will also be used

For peer review only

for comparing changes from baseline and the proportion of subjects experiencing abnormalities between treatment groups

- Sophia Observation withdrawal Symptoms-scale (SOS). The SOS scores will be descriptively summarised and analysed with a linear mixed model (with between-arm differences at baseline constrained to be 0).
- Neurodevelopment will be evaluated using the Bayley Scales of Infant and Toddler Development. The scale will be descriptively summarised and compared between treatment groups using linear regression analysis, with adjustment for centre and age group.

Quantitative variables will be summarized by the number of subjects analysed (N), mean, standard deviation (SD), median, minimum, and maximum. Categorical variables will be summarized by frequencies and percentages for each category.

Randomisation and kit assignment

Randomisation is stratified by both age subset and clinical site. Each stratum consists of 99 pre-randomised numbers (randomisation numbers), assigned to either clonidine or midazolam. These randomisation lists were prepared by a statistician not otherwise involved in the study, with the use of blocked randomisation consisting of small variable block sizes, in order to maintain blinding and conceal allocation.

At each clinical site, the kits marked with the random numbers for each age subset will be assigned to subjects in the order they are enrolled into the study. Each subject kit will contain two IMP boxes of seven vials, in order to supply the subject with enough medication for the maximum seven-day study period. The contents of each subject kit is outlined in Table 2.

For peer review only

Table 2. Composition of the subject kit for each age group

Age Subset	Age group	Formulation strength	Subject bodyweight	Number of boxes
1	<28 days	Low	≤3 kg	1
		Medium	>3 to <10 kg	1
2	28 days to <2 years	Medium	>3 to <10 kg	1
		High	≥10 kg to <47 kg	1
3	≥2 years to <18 years	High	≥10 kg to 85 kg	2 (in case >47 kg)

Emergency unblinding will be possible as for each randomised subject kit, two complete sets of sealed emergency envelopes have been prepared (one set for safekeeping by the investigator, the other will be delivered to the group responsible for overseeing pharmacovigilance of the study). Unblinding of the treatment allocation for a subject will be performed in an emergency that, in the opinion of the investigator, is warranted for a given subject for safety reasons.

Pharmacokinetic/pharmacodynamic endpoints

Pharmacokinetic/pharmacodynamic (PKPD) analyses will be carried out as a secondary endpoint to give further information on the dose-concentration -effect relationship primarily of clonidine, but also of midazolam. The PKPD model will be used to evaluate the dose scheme used in the study, and if necessary recommend an updated dose scheme for clinical use. PK/PD data will be modelled using nonlinear mixed effects modelling. The impact of drug metabolising enzyme genotype on inter-individual PKPD variability will be considered alongside other demographics in the multivariable covariate analysis. The link between PK and PD (COMFORT-B score) will be investigated by sequential and simultaneous modelling, possibly including an effect compartment. Item Response Theory (IRT) will be explored for PK-PD modelling, whereby effect is considered an

For peer review only

unobserved normally distributed latent variable, and each item score of COMFORT-B is allowed to contribute to this through link functions[46].

Mixed effects models will be fitted with maximum likelihood and addition of a single fixed effect will be guided by improvement of fit using the likelihood ratio test (change in objective function of 3.84 is significant at p=0.05 level for 1 degree of freedom by the chi-square distribution). Model evaluation will consist of goodness-of-fit (residual plots) and simulation-based diagnostics (visual predictive check), and parameter precision and robustness will be investigated with non-parametric bootstrapping.

Pharmacogenomic endpoints

Patients treated with sedatives and analgesics may respond very differently to the same dosage of medication. Several gene polymorphisms have been discovered to influence the pharmacokinetics and pharmacodynamics of clonidine, midazolam and morphine. PK data collected in this study can be interpreted more completely if data on pharmacogenomics are available.

Moreover, to study candidate polymorphisms gives further insight into the response of critically ill children to clonidine, midazolam, and morphine. Most candidate genes have been established in adult subjects, this study is an opportunity to study the effects in children. The candidate genes which will be genotyped are shown in Table 3.

Table 3. Candidate genes for linking pharmacogenomics to pharmacokinetic and pharmacodynamics endpoints.

**Midazolam**	**Clonidine**	**Morphine**
CYP3A4, CYP3A5, POR, ABCB1, GABA, MDR1, MRP1,	ADRA2A, CYP2D6.	COMT, OPRM1, OCT1, UGT2B7, ABCC3, MC1R, IL-

For peer review only

MRP2, MRP4, BRCP.		1Ra, IL-

Ethical considerations

Ad hoc considerations and measures have been set up to ensure safe and ethical conduct of this multi-national trial including a population requiring special protection, such as children. In order to guarantee the respect of ethical rules regardless of the country in which the trial is carried out, an ethical standard based on the EU ethical and legal framework has been agreed among centres. All submissions are based on the current European ethical and legal framework and on the international ethical principles and guidelines.

Based on the Council of Europe Convention on Human Rights and Biomedicine[47] and EU Ethical Recommendations 2008[48] research involving vulnerable populations is allowed if the results of the research are expected to provide real and direct benefit to the health of the patient, or some benefit for the population represented by the patient, and the trial will pose only minimal risk and burden to the individual concerned. The risk-benefit assessment has been carefully addressed considering IMP related risks (including pharmacologic properties and proposed dosing regimen of the IMP, information on use in target population, IMP development, IMP management) and expected benefits, the trial design (including study population inclusion and exclusion criteria), study related procedures, qualification of the study team and host sites, rights of patients (including informed consent and assent procedures and data protection and confidentiality). Minor increase over minimal risk is anticipated for the CLOSED trial, as the study deals with a novel treatment modality in vulnerable paediatric patients and carries minor risk based on the available information about the study drug as well as RCT related procedures. Important benefits for PICU population as a group are anticipated, since the study will provide clinically relevant and directly applicable results, expected to influence future standard of care.

For peer review only

The informed consent and assent process in this trial is in line with the applicable relevant regulatory documents, including Good Clinical Practice (GCP) guideline[49], Directive 2001/20/EC[50], the EC Detailed Guidance 2006[51], Directive 95/46/EC[52] , the International Ethical Guidelines for Biomedical Research Involving Human Subjects CIOMS-WHO (2002)[53], the Additional Protocol to the Oviedo Convention (2005)[54], ICH Topic E11, and the EC 2008 Paediatric Recommendations. Accordingly, clear and appropriate information sheets and informed consent forms for parents/legal representatives, and patient information sheets and assent forms are prepared for patients and customised according to the local requirements.

Given the nature of the trial, especially considering that ICU patients are often admitted as a result of emergency, deferred consent/assent, as described in Article 35 of the new European Regulation on clinical trials (EU) No 536/2014 of the European Parliament and of the Council[36], is foreseen if allowed by local legislation. Informed consent of the legal representative and, if feasible, the patient's assent will be sought for as soon as possible. If informed consent is not obtained, the possibility of withdrawing all collected data from the trial will be explained and appropriate measures, based on patient's decision will be taken. A record will remain in the subject's medical notes. In The Netherlands, deferred consent is permitted under national legislation ('Wet medisch-wetenschappelijk onderzoek met mensen', Paragraph 2 Article 6.4.).

As the protocol includes pharmacogenetic tests, informed consent and assent to perform genetic tests will be obtained separately, in line with the most relevant provisions in the field (International Declaration on Human Genetic Data, UNESCO, 2003[55]; Ethical Guidelines CIOMS-WHO, 2002). Accordingly, the participants will have the possibility to join the trial without participating in the pharmacogenetic part.

Finally, involvement of appropriate external expertise in the form of Data Safety Monitoring Board (DSMB), Independent Scientific Advisory Board (ISAB) and Patient Advisory Board (PAB) is foreseen. In details, the DSMB will review the study's safety data and assess subject safety data

For peer review only

throughout the study; the ISAB will input in the final protocols, monitor the progress of the project and ensure the on-going scientific and ethical integrity of the clinical trial; the PAB will advise the Sponsor to ensure that the subjects' rights and subjects' protection will outweigh any commercial considerations and conflicts of interest that may appear in relation to the project.

In accordance with GCP, as implemented at national level, the study protocol and related documents have been approved by the competent ethics committees in Rotterdam, the Netherlands, and submission is currently being prepared for the four other centres (Stockholm, Prague, Erlangen and Rome).

Dissemination

The results will be published in a peer-reviewed journal and shared with the worldwide medical community.

Discussion

Study design

Sedation management in the PICU mainly consists of the use of IV midazolam by continuous infusion. Midazolam has several advantages, but is by far not the ideal agent for longer-term sedation. Other agents have made their off-label entry into the PICU, mainly based on adult practice. One of these agents is clonidine which could have some specific advantages over midazolam. It is being used increasingly, but is still off-label. This study design allows for data collection on safety, efficacy, PK, pharmacogenomics and to a lesser extent neurological outcome. This multimodal approach generates data that is very useful for licensing clonidine for sedation in children. Moreover, application of different formulation strengths, allows for easier paediatric clinical

For peer review only

implementation as the current available formulation (Catapresan®) has only been developed for adults.

Role of researcher-driven studies, Seventh Framework Programme-funded projects

CLOSED is one of the 20 projects approved in the framework of the Seventh Framework Programme for Research that should specifically meet both criteria for scientific excellence and regulatory standards for high quality paediatric research, as prescribed by the Paediatric Regulation, in order to put paediatric drugs on the market. A very recent publication demonstrated that these projects face the need for overcoming the existing methodological and ethical difficulties affecting research in the paediatric population [5].

In fact, paediatric clinical trials are often multi-national and researchers need to know and apply rules from the regulatory, legal and ethical frameworks, acting both as investigators and as sponsor and/or other concerned parties. This is not simple considering the lack of harmonisation of clinical trial procedures among countries [http://www.ncbi.nlm.nih.gov/pubmed/26037896] and lead necessarily to prepare, agree and apply guidelines based on the EU rules.

Challenge to perform a trial compliant with GCP and other regulations

Clinical trials must follow the same rules notwithstanding if sponsored by industry or non-profit organisations, from GCP to the new clinical trials regulation [53] to recommendations and guidelines.

Accordingly, the new regulation [53] recognises that a large proportion of clinical trials are conducted by non-commercial sponsors, frequently supported by public funds or charities and that these trials should be encouraged.

However, this means a heavy work from researchers and a deep knowledge of both EU and national rules. This issue is aimed to be overcome by the international public-private cooperation between different stakeholders foreseen by EU-funded projects (researchers, clinicians, regulatory and

For peer review only

ethical experts, clinical research associates). On the other hand, it seems that the paediatric Consortia generated by the FP7 paediatric projects are conducting these studies and trials using a limited amount of money in comparison with the recognised cost of paediatric trials in an approved PIP which is estimated to be three to four times higher [5].

Challenges of maintaining double-blinding

Clonidine and midazolam have different half-lives: 1-3 hours for midazolam (up to 12h in neonates) and 9-17 hours for clonidine, thus maintaining double-blinding is challenging. PK-PD simulations have played a major role in the strategy to overcome this challenge. It has led to the implementation of a loading dose, which is in line with the current clinical recommendations for midazolam[44]. Target plasma levels have been achieved based on the literature[32, 43]. It should be noted, however, that the PK-PD relationship is assumed, but not formally studied. This study will contribute to closing this knowledge gap.

Risk to patients

Both clonidine and midazolam are frequently used sedatives in the PICU. However, a careful assessment of possible study-related risks has been performed. In general, side effects of IMP are the major cause of study-related risk. As midazolam is the golden standard treatment, the side effects do not contribute to an increased risk in this study. For clonidine, bradycardia and hypotension are the most clinically relevant side effects and are likely to occur most often during the loading doses and the early starting phase[33]. Therefore, a loading dose will be given as a 15-minute infusion instead of a direct bolus. Moreover, the setting where patients are being monitored consists of five well-equipped and experienced PICUs. Treatment of hypotension and/or bradycardia is common practice in the study population and should therefore not increase the risk to subjects dramatically.

For peer review only

Rebound hypertension could also be a side effect after sudden discontinuation of clonidine. In a comparable trial,[31] this was observed once in 64 patients and resolved quickly without any intervention. Monitoring of blood pressure up to 72 hours after IMP cessation has been included in the study protocol with the recommendation to treat hypertension according to local common practice.

Blood sampling is a possible risk to small children participating in drug research.[56] In this study, patients will not exceed the 3% of plasma volume, which is a general limit of study-related blood sampling.

In general, as most elements of this study are similar to current clinical practice, the study-related risk to patients is deemed low.

Pharmacogenetics

This study incorporates a pharmacogenetics assessment of recruited patients. Both PK and PD may be influenced by gene polymorphisms. The current proposed pharmacogenetics assessment has been based on the literature available, but a degree of flexibility has been built in when more information on these genes or medications will come available.

In accordance to the appropriate regulations,[51, 53, 55] separate consent and assent will be obtained from participants. This strategy enables the possibility of participating in the CLOSED-trial without the genetic assessment.

Anticipated difficulties

A comparable RCT, the SLEEPS-study, has been previously undertaken in the United Kingdom[33]. One of the major challenges faced by the investigators has been the recruitment. The sample size had been calculated on 1000 subjects. Unfortunately, only 129 subjects have been randomised. Contributing factors to this impaired recruitment have been identified by the investigators, including:

For peer review only

- Conflict with other studies
- Earlier extubation in elective cardiac surgery patients
- Parental issues and timing of consent
- Clinician's issues
- Research nurse time
- Delay in study start
- Compliance with the protocol.

It is likely that in the participating centres with excellent research facilities, multiple trials are being performed the same time. Children are not allowed to participate in multiple intervention studies, so we recommend to have a thorough look at the in- and exclusion criteria of studies and set up clear agreements about patient allocation.

Early extubation in cardiac surgery patients is now common practice, and estimations of recruitment rates have excluded these patients. However, it is likely that ventilation strategies change over the coming years. We have recognised the increasing use of non-invasive ventilation such as Non-Invasive Positive Pressure Ventilation (NIPPV). As these patients may still need continuous sedation, we will include these in the study. In general, patients on Continuous Positive Airway Pressure (CPAP) do not need additional sedation, thus these will be excluded.

Parental issues are to be expected in any clinical trial in the PICU. Parents having their child admitted to an ICU are under high stress and may therefore be reluctant to participate. In the calculations of estimated recruitment, a high refusal rate (approximately 40-50%) should be taken into account, based upon earlier experience with other trials. Also, a parent organisation is involved in the trial and they will provide information to parents from their perspective. In The Netherlands, deferred consent is used in emergency care trials. The Ethical Committee of the Erasmus Medical Centre, Rotterdam, has approved the use of deferred consent in this trial so this could be applied in the largest recruitment centre.

For peer review only

When faced with a critically ill child, doctors and nurses may tend towards conservatism. At the beginning of the SLEEPS trial, the involved health care professionals could have been afraid of the cardiovascular side effects of clonidine especially in unstable patients. Their study has shown that its use is relatively safe in critically ill children, as well as other literature.[34, 57] Moreover, in the CLOSED consortium recruitment centres, a lot of clinical experience with clonidine is has been built so caregivers may be less hesitant to support this clinical trial.

Research nurse time is always an issue, especially in the 24/7-business of an ICU. In the larger centres, dedicated researchers/nurses have been identified and trained adequately to perform this trial. In the smaller centres, PIs may be able to coordinate the lower amounts of participants better.

During the first months of patient recruitment in two centres, no patients could be included. In the majority of cases, exclusion criteria made the screened patients ineligible. No consent was given in the very few eligible patients. Therefore, a revision of the exclusion criteria have been made.

The major exclusion criteria on which patients failed screening are:

- **Anticipated need for sedation <24 hours**. It has been considered to change this duration. It has been decided however to keep this minimum as the therapeutic aim of the study is long-term sedation.
- **No sedation despite ventilation.** To our surprise, many newborn patients on the ventilator would not need any sedation, or were comfortable on low-dose opioids. This could not be mitigated.
- **Circulatory insufficiency.** The Goldstein criteria[58] have been used for the definition of this exclusion criterion. However, many patients received inotropic or vasopressor agents as supportive therapy in both recruiting centres. We have therefore modified these criteria and removed the use of inotropes/vasopressors in the second protocol amendment.

- **Neurological pathology.** It has been suggested to include patients with minor head trauma, but as the clinical picture may change very rapidly, we have decided not to include these patients into the trial.
- **Use of clonidine in the last 7 days.** As clonidine has a long half-life, patients would be excluded if they have received clonidine for any indication in the previous 7 days. However, for midazolam, there was no similar exclusion criterion. instead, patients would undergo an extra PK blood sample before start of the study medication. This has now been introduced for clonidine as well in the second protocol amendment.

A delay in study start has been encountered in the CLOSED study. Several obstacles, such as obtaining the appropriate licenses for handling, importing and exporting controlled drugs, have caused a significant delay in recruitment start. A second major issue has also been an amendment of the dose reduction scheme (see paragraph 'Study treatments'). A third issue has been the generation of robust quality data that is sensitive enough to support a future PUMA application as well as the IMPD and clinical trial application [59].

Compliance with the protocol was a significant issue in the SLEEPS trial, as they provided a strict sedation regimen. Therefore, the CLOSED sedation regimen merely reflects clinical practice, although some important aspects may cause difficulties in clinical practice. The lockout periods for example, are not according to clinical practice and special attention needs to be paid by the research nurses and other investigators to keep the protocol compliance as tight as possible. When problems are recognised, the possibility of amendments should be discussed early. Early recognition is facilitated by implemented monthly investigators' teleconferences.

For peer review only

Also, there will be an ongoing evaluation of the COMFORT-B scale training of the nurses in the participating centres. Good inter-observer reliability is defined by a Cohen's kappa >0.65 and nurses in all centres have received training by trained nurses.

In summary, many known challenges should be managed by appropriate measures taken. However, there could always be unknown challenges and it is therefore crucial to have regular contact between principal investigators of each recruitment site.

Trial status

The trial has started enrolling subjects in the summer of 2016 in Rotterdam and Erlangen, where ethical approval is available. Recruitment in other sites will follow when ethical approval becomes available. The second amendment including changes of the exclusion criteria will be submitted to the ethical and regulatory institutions in the end of 2016.

Author contributions

AN: Coordinator of the trial and initiator of the study. Collaborated on writing this manuscript.

MB: Coordinating researcher in main trial site, contributed specifically to the pharmacogenomics and the NIMP section of the protocol. Collaborated on writing this manuscript. Responsible for data collection.

MvD: Collaborated on establishment of the primary and secondary endpoints of this trial.

JvR: Main biostatistician responsible for the sample size calculation and statistical analyses.

JS: Collaborated on statistical analyses and performed PK modelling.

YS: Collaborated on the statistical analyses and performed PK modelling,

For peer review only

DR: Responsible for compiling the sections of the protocol into one document, collaborated on pharmaceutical development.

JW: Responsible for the ethics section.

LR: Collaborated on the ethics and regulatory section.

SH: Responsible for pharmaceutical development.

TM: Responsible for the laboratory analyses and sampling plan.

VG: Responsible for ethics and regulatory sections of the protocol.

PL: Collaborated on trial design and study population. Principal Investigator at Swedish site. Responsible for data collection.

PP: Collaborated on trial design and study population. Principal Investigator at Czech site. Responsible for data collection.

AS: Collaborated on trial design and study population. Responsible for data collection.

DT: Principal Investigator and initiator of this study. Collaborated on trial design.

Competing interests

None declared.

For peer review only

Funding

This project has received funding from the European Union's Seventh Framework Programme for research, technological development and demonstration under grant agreement n.602453

For peer review only

References

1. Ceci, A., et al., *Medicines for children licensed by the European Medicines Agency (EMEA): the balance after 10 years.* Eur J Clin Pharmacol, 2006. **62**(11): p. 947-52.
2. Neubert, A., et al., *Defining off-label and unlicensed use of medicines for children: results of a Delphi survey.* Pharmacol Res, 2008. **58**(5-6): p. 316-22.
3. European Medicines Agency, *Report on the survey of all paediatric uses of medicinal products in Europe*. 2010, EMA.
4. European Commision, *Regulation (EC) No. 1901/2006 of the European Parliament and of the Council of 12 December 2006 on medicinal products for paediatric use and amending Regulation (EEC) No 1768/92, Directive 2001/20/EC, Directive 2001/83/EC and Regulation (EC) No 726/2004*. 2006, European Commision: Strasbourg. p. 1-19.
5. Ruggieri, L., et al., *Successful private-public funding of paediatric medicines research: lessons from the EU programme to fund research into off-patent medicines.* Eur J Pediatr, 2015. **174**(4): p. 481-91.
6. EMA/PDCO *Revised priority list for studies into off-patent paediatric medicinal products.* 2013.
7. British Paediatric Association, *The Care of Critically Ill Children. Report of a Multidisciplinary Working Party on Intensive Care* 1993, BPA: London.
8. UK Paediatric Intensive Care Society, *PICS Standards.* 2001.
9. Playfor, S.D. and H. Vyas, *Sedation in critically ill children.* Curr Paediatr, 2000. **10**(1): p. 1-4.
10. Tobias, J.D. and G.E. Rasmussen, *Pain management and sedation in the pediatric intensive care unit.* Pediatr Clin North Am, 1994. **41**(6): p. 1269-92.
11. Baarslag, M.A., et al., *Pharmacological sedation management in the pediatric intensive care unit* Journal of Pharmacy and Pharmacology, 2016. **Accepted for publication.**

For peer review only

12. Playfor, S., et al., *Consensus guidelines on sedation and analgesia in critically ill children.* Intensive Care Med, 2006. **32**(8): p. 1125-36.

13. Playfor, S., D. Thomas, and I. Choonara, *Recollection of children following intensive care.* Archives of Disease in Childhood, 2000. **83**(5): p. 445-448.

14. Klupp, H., et al., *Effects of Clonidine on central sympathetic tone.* Eur J Pharmacol, 1970. **10**(2): p. 225-9.

15. Drew, G.M. and S.B. Whiting, *Evidence for two distinct types of postsynaptic alpha-adrenoceptor in vascular smooth muscle in vivo.* Br J Pharmacol, 1979. **67**(2): p. 207-15.

16. Ramesh, V.J., N. Bhardwaj, and Y.K. Batra, *Comparative study of oral clonidine and diazepam as premedicants in children.* Int J Clin Pharmacol Ther, 1997. **35**(5): p. 218-21.

17. Nishina, K., et al., *Clonidine in paediatric anaesthesia.* Paediatr Anaesth, 1999. **9**(3): p. 187-202.

18. Hoffman, W.E., et al., *Dexmedetomidine improves neurologic outcome from incomplete ischemia in the rat. Reversal by the alpha 2-adrenergic antagonist atipamezole.* Anesthesiology, 1991. **75**(2): p. 328-32.

19. Laudenbach, V., et al., *Effects of alpha(2)-adrenoceptor agonists on perinatal excitotoxic brain injury: comparison of clonidine and dexmedetomidine.* Anesthesiology, 2002. **96**(1): p. 134-41.

20. Yuan, S.Z., et al., *Hypoxic-ischaemic brain damage in immature rats: effects of adrenoceptor modulation.* Eur J Paediatr Neurol, 2001. **5**(1): p. 29-35.

21. Donello, J.E., et al., *alpha(2)-Adrenoceptor agonists inhibit vitreal glutamate and aspartate accumulation and preserve retinal function after transient ischemia.* J Pharmacol Exp Ther, 2001. **296**(1): p. 216-23.

22. Zhang, Y., *Clonidine preconditioning decreases infarct size and improves neurological outcome from transient forebrain ischemia in the rat.* Neuroscience, 2004. **125**(3): p. 625-31.

For peer review only

23. Wolf, A., *Development and evaluation of pain and the stress response.*, in *Paediatric Anaesthesia*, B. Bissonnette and B. Dalens, Editors. 2002, McGraw-Hill: New York. p. 186-200.

24. Burch, M., et al., *Influence of cardiopulmonary bypass on water balance hormones in children.* Br Heart J, 1992. **68**(3): p. 309-12.

25. Dorman, T., et al., *Effects of clonidine on prolonged postoperative sympathetic response.* Crit Care Med, 1997. **25**(7): p. 1147-52.

26. Kulka, P.J., M. Tryba, and M. Zenz, *Preoperative alpha2-adrenergic receptor agonists prevent the deterioration of renal function after cardiac surgery: results of a randomized, controlled trial.* Crit Care Med, 1996. **24**(6): p. 947-52.

27. Hohage, H., E. Schlatter, and J. Greven, *Effects of moxonidine and clonidine on renal function and blood pressure in anesthetized rats.* Clin Nephrol, 1997. **47**(5): p. 316-24.

28. Wijeysundera, D.N., J.S. Naik, and W.S. Beattie, *Alpha-2 adrenergic agonists to prevent perioperative cardiovascular complications: a meta-analysis.* Am J Med, 2003. **114**(9): p. 742-52.

29. Pohl-Schickinger, A., et al., *Intravenous clonidine infusion in infants after cardiovascular surgery.* Paediatr Anaesth, 2008. **18**(3): p. 217-22.

30. Martin, J., et al., *Evidence and consensus-based German guidelines for the management of analgesia, sedation and delirium in intensive care--short version.* Ger Med Sci, 2010. **8**: p. Doc02.

31. Ambrose, C., et al., *Intravenous clonidine infusion in critically ill children: dose-dependent sedative effects and cardiovascular stability.* Br J Anaesth, 2000. **84**(6): p. 794-6.

32. Arenas-Lopez, S., et al., *Use of oral clonidine for sedation in ventilated paediatric intensive care patients.* Intensive Care Med, 2004. **30**(8): p. 1625-9.

For peer review only - http://bmjopen.bmj.com/site/about/guidelines.xhtml

33. Wolf, A., et al., *Prospective multicentre randomised, double-blind, equivalence study comparing clonidine and midazolam as intravenous sedative agents in critically ill children: the SLEEPS (Safety profiLe, Efficacy and Equivalence in Paediatric intensive care Sedation) study.* Health Technol Assess, 2014. **18**(71): p. 1-212.

34. Hunseler, C., et al., *Continuous infusion of clonidine in ventilated newborns and infants: a randomized controlled trial.* Pediatr Crit Care Med, 2014. **15**(6): p. 511-22.

35. Duffett, M., et al., *Clonidine in the sedation of mechanically ventilated children: A pilot randomized trial.* J Crit Care, 2014. **29**(5): p. 758-63.

36. European Union, *European Regulation on clinical trials (EU) No 536/2014 of the European Parliament and of the Council of 16 April 2014 on clinical trials on medicinal products for human use, and repealing Directive 2001/20/EC.* 2014.

37. Hall, J.E., T.D. Uhrich, and T.J. Ebert, *Sedative, analgesic and cognitive effects of clonidine infusions in humans.* Br J Anaesth, 2001. **86**(1): p. 5-11.

38. Sheng, Y. and J.F. Standing, *Pharmacokinetic reason for negative results of clonidine sedation in long-term-ventilated neonates and infants.* Pediatr Crit Care Med, 2015. **16**(1): p. 92-3.

39. Ista, E., et al., *Assessment of sedation levels in pediatric intensive care patients can be improved by using the COMFORT "behavior" scale.* Pediatr Crit Care Med, 2005. **6**(1): p. 58-63.

40. von Baeyer, C.L. and L.J. Spagrud, *Systematic review of observational (behavioral) measures of pain for children and adolescents aged 3 to 18 years.* Pain, 2007. **127**(1-2): p. 140-50.

41. Ista, E., et al., *Psychometric evaluation of the Sophia Observation withdrawal symptoms scale in critically ill children.* Pediatr Crit Care Med, 2013. **14**(8): p. 761-9.

42. Bayley, N., *Bayley Scales of Infant Development, 2nd edition.* 1993, San Antonio, TX: The Psychological Corporation.

For peer review only

43. de Wildt, S.N., et al., *Pharmacodynamics of midazolam in pediatric intensive care patients.* Ther Drug Monit, 2005. **27**(1): p. 98-102.

44. Nederlands Kenniscentrum voor Farmacotherapie bij Kinderen, *Kinderformularium*. 2014.

45. Klingenberg, B., *A new and improved confidence interval for the Mantel-Haenszel risk difference.* Stat Med, 2014. **33**(17): p. 2968-83.

46. Ueckert, S., et al., *Improved utilization of ADAS-cog assessment data through item response theory based pharmacometric modeling.* Pharm Res, 2014. **31**(8): p. 2152-65.

47. European Commission, *Ethical Considerations for Clinical Trials on Medicinal Products Conducted with the Paediatric Population*. 2008.

48. European Union, *Ethical considerations for clinical trials on medicinal products conducted with the paediatric population.* Eur J Health Law, 2008. **15**(2): p. 223-50.

49. ICH Expert Working Group, *Guideline for Good Clinical Practice*. 1996.

50. European Commission, *Directive 2001/20/EC of the European Parliament and of the Council of 4 April 2001 on the approximation of the laws, regulations and administrative provisions of the Member States relating to the implementation of good clinical practice in the conduct of clinical trials on medicinal products for human use.* 2001.

51. European Commision, *Detailed guidance on the application format and documentation to be submitted in an application for an Ethics Committee opinion on the clinical trial on medicinal products for human use. Revision 1.* 2006.

52. European Commision, *Directive 95/46/EC of the European Parliament and of the Council of 24 October 1995 on the protection of individuals with regard to the processing of personal data and on the free movement of such data*. 1995.

53. Council for International Organizations of Medical Sciences (CIOMS)-WHO, *International Ethical Guidelines for Biomedical Research Involving Human Subjects.* 2002.

For peer review only

909 54. Council of Europe, *Additional Protocol to the Convention on Human Rights and Biomedicine, concerning Biomedical Research.* 2005.

911 55. UNESCO, *International Declaration on Human Genetic Data*. 2003.

912 56. Heidmets, L.T., et al., *Blood loss related to participation in pharmacokinetic study in preterm neonates.* Neonatology, 2011. **100**(2): p. 111-5.

914 57. Bergendahl, H.T., S. Eksborg, and P.A. Lonnqvist, *Low-dose intravenous clonidine in children: plasma concentrations and haemodynamic response.* Acta Anaesthesiol Scand, 1997. **41**(3): p. 381-4.

917 58. Goldstein, B., et al., *International pediatric sepsis consensus conference: definitions for sepsis and organ dysfunction in pediatrics.* Pediatr Crit Care Med, 2005. **6**(1): p. 2-8.

919 59. Hanning, S.M., et al., *Quality and clinical supply considerations of Paediatric Investigation Plans for IV preparations-A case study with the FP7 CloSed project.* Int J Pharm, 2016.

921

922

For peer review only

Figure 1. Minimum time line of infusion rate increases for all loading dosis of IMP administered for subjects (half doses in neonates).

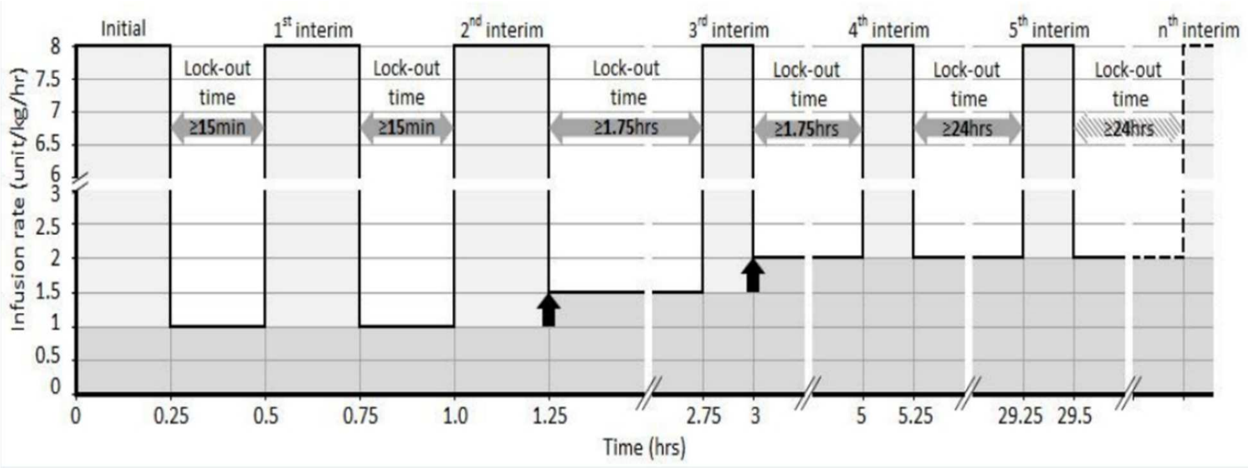

Figure 2. The proportion of average midazolam plasma concentration above the target value (0.311 mg/l) after the time of 5th bolus according to the proposed dose scheme(left) and the maximum Dutch recommended dosing regimen(right).

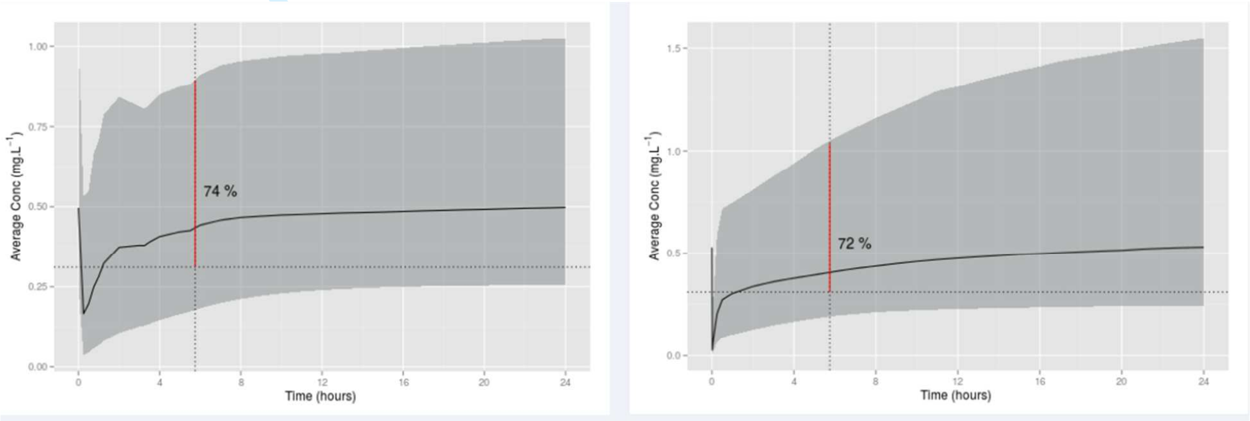

For peer review only

CONSORT 2010 checklist of information to include when reporting a randomised trial*

Section/Topic	Item No	Checklist item	Reported on page No
**Title and abstract**			
	1a	Identification as a randomised trial in the title	1
	1b	Structured summary of trial design, methods, results, and conclusions (for specific guidance see CONSORT for abstracts)	3-4
**Introduction**			
Background and objectives	2a	Scientific background and explanation of rationale	5-9
	2b	Specific objectives or hypotheses	9
**Methods**			
Trial design	3a	Description of trial design (such as parallel, factorial) including allocation ratio	10-11
	3b	Important changes to methods after trial commencement (such as eligibility criteria), with reasons	32-33
Participants	4a	Eligibility criteria for participants	10
	4b	Settings and locations where the data were collected	10
Interventions	5	The interventions for each group with sufficient details to allow replication, including how and when they were actually administered	11-13
Outcomes	6a	Completely defined pre-specified primary and secondary outcome measures, including how and when they were assessed	13-15
	6b	Any changes to trial outcomes after the trial commenced, with reasons	N/a
Sample size	7a	How sample size was determined	16-18
	7b	When applicable, explanation of any interim analyses and stopping guidelines	N/a
Randomisation:			
Sequence generation	8a	Method used to generate the random allocation sequence	21
	8b	Type of randomisation; details of any restriction (such as blocking and block size)	21-22
Allocation concealment mechanism	9	Mechanism used to implement the random allocation sequence (such as sequentially numbered containers), describing any steps taken to conceal the sequence until interventions were assigned	21-22
Implementation	10	Who generated the random allocation sequence, who enrolled participants, and who assigned participants to interventions	21
Blinding	11a	If done, who was blinded after assignment to interventions (for example, participants, care providers, those	

For peer review only - http://bmjopen.bmj.com/site/about/guidelines.xhtml

		assessing outcomes) and how	21-22
	11b	If relevant, description of the similarity of interventions	16-17
Statistical methods	12a	Statistical methods used to compare groups for primary and secondary outcomes	18-20
	12b	Methods for additional analyses, such as subgroup analyses and adjusted analyses	19-22
**Results**			
Participant flow (a diagram is strongly recommended)	13a	For each group, the numbers of participants who were randomly assigned, received intended treatment, and were analysed for the primary outcome	N/a
	13b	For each group, losses and exclusions after randomisation, together with reasons	N/a
Recruitment	14a	Dates defining the periods of recruitment and follow-up	N/a
	14b	Why the trial ended or was stopped	N/a
Baseline data	15	A table showing baseline demographic and clinical characteristics for each group	N/a
Numbers analysed	16	For each group, number of participants (denominator) included in each analysis and whether the analysis was by original assigned groups	N/a
Outcomes and estimation	17a	For each primary and secondary outcome, results for each group, and the estimated effect size and its precision (such as 95% confidence interval)	N/a
	17b	For binary outcomes, presentation of both absolute and relative effect sizes is recommended	N/a
Ancillary analyses	18	Results of any other analyses performed, including subgroup analyses and adjusted analyses, distinguishing pre-specified from exploratory	N/a
Harms	19	All important harms or unintended effects in each group (for specific guidance see CONSORT for harms)	N/a
**Discussion**			
Limitations	20	Trial limitations, addressing sources of potential bias, imprecision, and, if relevant, multiplicity of analyses	29-34
Generalisability	21	Generalisability (external validity, applicability) of the trial findings	N/a
Interpretation	22	Interpretation consistent with results, balancing benefits and harms, and considering other relevant evidence	N/a
**Other information**			
Registration	23	Registration number and name of trial registry	2
Protocol	24	Where the full trial protocol can be accessed, if available	N/a
Funding	25	Sources of funding and other support (such as supply of drugs), role of funders	35

*We strongly recommend reading this statement in conjunction with the CONSORT 2010 Explanation and Elaboration for important clarifications on all the items. If relevant, we also recommend reading CONSORT extensions for cluster randomised trials, non-inferiority and equivalence trials, non-pharmacological treatments, herbal interventions, and pragmatic trials. Additional extensions are forthcoming: for those and for up to date references relevant to this checklist, see www.consort-statement.org.

For peer review only - http://bmjopen.bmj.com/site/about/guidelines.xhtml

BMJ Open

The CLOSED- trial; CLOnidine compared to midazolam for SEDation of paediatric patients in the intensive care unit: study protocol for a multicentre randomised controlled trial

Journal:	*BMJ Open*
Manuscript ID	bmjopen-2017-016031.R1
Article Type:	Protocol
Date Submitted by the Author:	26-Apr-2017
Complete List of Authors:	Neubert, Antje; Friedrich-Alexander-University Erlangen/Nuremberg, Loschgestr. 15, 91054, Erlangen, Germany<br>Baarslag, Manuel; Erasmus MC-Sophia Children's Hospital, Intensive Care and Pediatric Surgery<br>van Dijk , Monique; Erasmus MC Sophia Childrens Hosp<br>van Rosmalen, Joost; Erasmus MC, Biostatistics<br>Standing, Joseph; Institute of Child Health, Infectious Diseases and Microbiology Unit<br>Sheng, Yucheng; University College London, Institute of Child Health<br>Rascher, Wolfgang; Friedrich-Alexander-University Erlangen/Nuremberg, Loschgestr. 15, 91054, Erlangen, Germany<br>Roberts, Deborah ; Therakind Ltd<br>Winslade, Jackie; Therakind Ltd<br>Rawcliffe, Louise; Therakind Ltd<br>Hanning, Sara; University College London, UCL School of Pharmacy<br>Metsvaht, Tuuli; Tartu Ulikooli Kliinikum, Clinic of Anaesthesiology and Intensive Care<br>Giannuzzi, Viviana; Fondazione per la Ricerca Farmacologica Gianni Benzi ONLUS<br>Larsson, Peter; Astrid Lindgrens Barnsjukhus, Pediatric Anesthesia and Intensive Care<br>Pokorná, Pavla; Univerzita Karlova v Praze 1 lekarska fakulta, Department of Pediatrics<br>Simonetti, Alessandra; Ospedale Pediatrico Bambino Gesu, Clinical Trial Center<br>Tibboel, Dick; Erasmus MC-Sophia Children's Hospital, Intensive Care and Pediatric Surgery
**Primary Subject Heading**:	Paediatrics
Secondary Subject Heading:	Intensive care
Keywords:	Paediatric intensive & critical care < INTENSIVE & CRITICAL CARE, Clinical trials < THERAPEUTICS, Paediatric anaesthesia < PAEDIATRICS

For peer review only

The CLOSED- trial; CLOnidine compared to midazolam for SEDation of paediatric patients in the intensive care unit: study protocol for a multicentre randomised controlled trial

List of authors:

Antje Neubert[1], Manuel A Baarslag*[2], Monique van Dijk[2], Joost van Rosmalen[3], Joseph F Standing[4], Yucheng Sheng[4], Wolfgang Rascher[1], Deborah Roberts[5], Jackie Winslade[5], Louise Rawcliffe[5], Sara M Hanning[6], Tuuli Metsvaht[7], Viviana Giannuzzi[8], Peter Larsson[9], Pavla Pokorná[10], Alessandra Simonetti[11] and Dick Tibboel[2]; On behalf of the CLOSED Consortium

***Corresponding Author:**

Manuel Alberto Baarslag

Room Sp-2430

Wytemaweg 80

3015 CN Rotterdam

The Netherlands

For peer review only

Telephone: +31107030380

E-mail: m.baarslag@erasmusmc.nl

Author affiliations

1. Department of Children and Adolescents Medicine, Friedrich-Alexander-University Erlangen/Nuremberg, Erlangen, Germany
2. Intensive Care and Department of Pediatric Surgery, Erasmus MC-Sophia Children's Hospital, Rotterdam, The Netherlands
3. Department of Biostatistics, Erasmus MC, Rotterdam, The Netherlands
4. Institute of Child Health, University College London, London, United Kingdom
5. Therakind Ltd, London, United Kingdom
6. UCL School of Pharmacy, University College London, London, United Kingdom
7. Clinic of Anaesthesiology and Intensive Care, Tartu University Hospital, Tartu, Estonia
8. Fondazione per la Ricerca Farmacologica Gianni Benzi onlus, Valenzano, Italy
9. Department of Pediatric Anesthesia and Intensive Care, Astrid Lindgrens Childrens Hospital, Karolinska University Hospital, Stockholm, Sweden
10. Department of Pediatrics, First Faculty of Medicine, Charles University Prague, Czech Republic
11. Clinical Trial Center, Bambino Gesù Children's Hospital, Rome, Italy

Trial registration: EudraCT: 2014-003582-24; Clinicaltrials.gov: NCT02509273

Key words: Sedation, children, critical illness, paediatrics, pharmacology

Word count: 6630

For peer review only

List of abbreviations

BIS: Bispectral Index Monitoring; CNS: Central nervous system; CPAP: Continuous Positive Airway Pressure; DSMB: Data Safety Monitoring Board; ECMO: Extracorporeal Membrane Oxygenation; GA: Gestational Age; GABA: Gamma-Amino Butyric Acid; IMP: Investigational Medical Product; ISAB: Independent Scientific Advisory Board; IV: Intravenously; NIPPV: Non-invasive Positive Pressure Ventilation; NMDA: N-methyl-D-aspartate; NIMP: Non-Investigational Medical Product; NISS: Nurses' Interpretation of Sedation Score; NRS: Numeric Rating Scale; PIP: Paediatric Investigation Plan ; PICU: Paediatric Intensive Care Unit; PD: Pharmacodynamics; PK: Pharmacokinetics; SmPC: Summary of Product Characteristics; PUMA; Paediatric-Use Marketing Authorisation

Abstract

Introduction:

Sedation is an essential part of paediatric critical care. Midazolam, often in combination with opioids, is the current gold standard drug. However, as it is a far-from-ideal agent, clonidine is increasingly being used in children. This drug is prescribed off-label for this indication, as many drugs in paediatrics are. Therefore, the CLOSED trial aims to provide data on the pharmacokinetics, safety and efficacy of clonidine for the sedation of mechanically ventilated patients in order to obtain a paediatric-use marketing authorisation (PUMA).

Methods and analysis:

The CLOSED study is a multi-centre, double-blind, randomised, active-controlled non-inferiority trial with a 1:1 randomisation between clonidine and midazolam. Both treatment groups are stratified according to age in three groups with the same size: <28 days (n=100), 28 days to <2 years (n=100) and 2-18 years (n=100). The primary endpoint is defined as the occurrence of sedation failure within the study period. Secondary endpoints include a PK/PD relationship,

For peer review only

pharmacogenetics, occurrence of delirium and withdrawal syndrome, opioid consumption and neurodevelopment in the neonatal age group. Logistic regression will be used for the primary endpoint, appropriate statistics will be used for the secondary endpoints.

Ethics:

Written informed consent will be obtained from the parents/caregivers. Verbal or deferred consent will be used in the sites where national legislation allows. The study has IRB approval at recruiting sites. The results will be published in a peer-reviewed journal and shared with the worldwide medical community.

Registration:

EudraCT: 2014-003582-24; Clinicaltrials.gov: NCT02509273

Strengths and limitations of this study

- The dosing scheme being used in this trial is based on detailed PK-PD modelling data
- Pain and sedation are measured using a standardised assessment scheme (COMFORT-B Score) across all participating units
- Besides information on the efficacy and safety of clonidine in critically ill children, this trial will provide population-based PK information in this specific patient population
- Age-specific formulations are being used in this trial, which can be marketed afterwards
- A previous similar trial indicated challenging recruitment, this could be a potential threat to this trial

For peer review only

Introduction

Unlicensed and off-label drug use

In Europe, less than 30% of marketed drugs include results from paediatric clinical trials and other information on paediatric use in their documentation (Summary of Product Characteristics, SPC, or Product Leaflet)[1]. This results in widespread off-label use in paediatrics, especially in the case of old drugs that have never received a paediatric authorisation. Off-label paediatric use (including all the uses not listed in the SmPC[2]) in Europe accounts for 45-60% of the total number of prescriptions, with rates of up to 90% in patients admitted to neonatal or paediatric intensive care units[3].

The entry into force of the Paediatric Regulation in 2007[4] gave an important stimulus to support the development of medicines for children by introducing a specific measure to favour work on off-patent medicines, the so-called Paediatric Use Marketing Authorisation (PUMA). A PUMA application should include the submission of paediatric data in accordance with an agreed Paediatric Investigation Plan (PIP).

According to article 40 of the Regulation, the European Commission (EC) reserved funds 'to develop off-patent medicinal products with recognised therapeutic interest for children and included in a 'Priority List' adopted by the EMA (European Medicines Agency) through its Paediatric Committee (Seventh Framework Programme for Research HEALTH-(2007-2013) Programme area - topic 4.2-1). Among 20 projects approved[5], the CLOSED project was granted with the aim to develop an age-appropriate formulation of clonidine for sedation in PICU, in line with the EMA "Revised Priority List for Studies into Off-Patent Paediatric Medicinal Products" 2012[6]. In order to specifically meet this therapeutic need, a comprehensive development plan in the form of a Paediatric Investigation Plan (PIP) was submitted to the EMA Paediatric Committee (PDCO) in July 2012 and approved in January 2013. In March 2015, a modification of the PIP was proposed after the finalisation of the clinical study protocol. This modification was approved by the PDCO in August 2015.

For peer review only - http://bmjopen.bmj.com/site/about/guidelines.xhtml

Analgosedation in the PICU

Approximately 2% of all paediatric patients admitted to hospital require treatment in the Paediatric Intensive Care Unit (PICU)[7]. Most PICU admissions are unplanned emergencies, mainly in the context of congenital heart diseases (40%), respiratory diseases (20%), major trauma (15%) and neurological problems other than trauma (<10%)[8].

Often, mechanical ventilation is required for facilitating recovery after major surgery or for treating respiratory failure. In most of these cases, analgosedation using potent opioids and sedatives is mandatory to reduce metabolic rate and oxygen demand, assist mechanical ventilation, avoid inadvertent self-extubation and lower anxiety and distress[9, 10]. To this effect, a variety of agents are used in the NICU and PICU, such as opioids, GABA-receptor agonists, NMDA-receptor antagonists and α2-receptor agonists[11].

Opioids

The main opioids used in PICU and NICU are morphine, fentanyl and remifentanil. Opioids bind to the μ receptor in the central nervous system (CNS), and provide analgesia as well as sedation.

GABA-receptor agonists

GABA receptors have an inhibitory effect on the CNS. Benzodiazepines agonise these receptors and therefore have sedative, anxiolytic and anticonvulsant properties. Disadvantages are paradoxal reactions (agitation and confusion) and tolerance and withdrawal after prolonged use.

Propofol is a unique agent with GABA-ergic properties as well as anti-NMDA and sodium channel blocking effects. Propofol is not suitable for long-term sedation, due to the perceived risk of propofol infusion syndrome, a metabolic derangement accompanied by severe metabolic acidosis, hyperkalaemia, hyperlipidaemia, rhabdomyolysis and organ failure, associated with an increased risk of mortality[12].

For peer review only

Midazolam

Midazolam is the most widely used sedative agent in the PICU. It is a short-acting benzodiazepine (Tmax of 5-10 minutes after IV injection) with a half-life of 1-3 hours (up to 12 hours in neonates). Besides sedation and anxiolysis, midazolam also provides anterograde amnesia, thus minimizing children's recall of unpleasant experiences after a PICU admission.[13] Midazolam is mainly metabolised to the equipotent metabolite 1-OH-midazolam, and then glucuronidated to the renally excreted 1-OH-MDZ-glucuronide. Its main adverse effects include tolerance, dependence and withdrawal syndrome following discontinuation.

Clonidine

Clonidine agonises the alpha-2-adrenergic receptor. The reduced sympathetic outflow[14] in the CNS results in sedation, anxiolysis and analgesia[15, 16]. Because of its analgesic properties, it has been used as an adjunct in surgical procedures as premedication or as a supplementary agent in regional anaesthesia[17]. The reduction of sympathetic outflow associated with clonidine may have specific benefits in critically ill children. Alpha-2 agonists can improve neurological outcome associated with ischemic cerebral injury[18-20], the mechanism of action of which is unclear but may be due to suppression of extracellular glutamate and aspartate release during energy failure[21]. Recent data have also demonstrated that pre-conditioning before the insult can both reduce infarct size and improve neurological outcome after insult[22]. Surgery and critical illness are associated with a variety of stress responses, which can result in organ dysfunction[23]: Renal function deteriorates after both adult and paediatric cardiac surgery, and this effect is due in part to the increase in sympathetic outflow and the rise in circulating vasoconstrictors such as noradrenaline, vasopressin and angiotensin[24, 25]. Clonidine has been demonstrated to suppress these responses and prevent the associated decline in renal function after adult cardiac surgery[26]. In addition, clonidine has independent local effects on tubular function which promote both diuresis and natriuresis[27]. In terms of cardiovascular responses, reduction in stress responses by alpha-2 agonists have been shown to

For peer review only

reduce perioperative myocardial ischemia in adults undergoing cardiac and non-cardiac surgery[28]. As the mechanism of action of clonidine is very different from the GABA-agonist midazolam, the hypothesis is postulated that it may be less associated with paediatric delirium and withdrawal syndrome. Moreover, clonidine is one of the agents used for the treatment of withdrawal syndrome[29].

Previous clinical trials

Clonidine is commonly used off-label in paediatric anaesthesia and intensive care medicine. Its use is recommended for the sedation of critically ill children in PICUs by the UK and German consensus guidelines[12, 30] and local hospital guidelines across Europe. Unfortunately, despite this widespread use of clonidine there are limited data on efficacy, dose requirement and safety when used for sedation on PICUs. A number of studies have been performed[31-35], however, to overcome this knowledge gap. See table 1 for a short overview.

Table 1. An overview of paediatric studies involving clonidine for sedation in the intensive care unit.

**Study**	**Sample size and age**	**Design**	**Outcome**
**Ambrose et al. [31]**	N=30, 0-10 years	Three-step: IV Low-dose vs. high-dose (variable dose together with midazolam), 3rd group fixed dose	No adverse effects on haemodynamics, sufficient sedation in combination with midazolam
**Arenas-Lopez et al. [32]**	N=24, 0-5 years	Prospective cohort study, oral clonidine as additive to morphine/lorazepam	Opioid- and benzodiazepine sparing, safe and effective
**Wolf et al. [33]**	N=129, 0-15 years	Double-blind, randomised controlled trial of IV clonidine vs. midazolam	No difference in effectivity, underpowered due to recruitment problems
**Hünseler et al. [34]**	N=219, 0-2 years	Double-blind, randomised controlled trial of IV clonidine vs. midazolam	Opioid- and benzodiazepine-sparing in neonatal age group
**Duffett et al. [35]**	N=50, 0-18 years	Double-blind, randomised controlled trial of oral clonidine vs. placebo in	No significant difference in effectivity, study with clonidine clinically feasible

For peer review only

		addition to physician-driven sedation	

In summary, when used in addition to standard sedation management, clonidine has a benzodiazepine- and opioid-sparing effect. Comparative studies showed equivalence, but were either underpowered[33] or set up as a pilot trial[35].

Study aims

For the purpose of sedation of intubated and mechanically ventilated paediatric patients, this clinical study sets out to provide the data needed for a Paediatric Use Marketing Authorisation (PUMA) application for the use of clonidine in PICU patients:

- data on efficacy and safety of clonidine compared to midazolam.
- PK data on both clonidine and midazolam in critically ill children and adolescents, using a population-based approach.

Together with the development of age- and weight-adapted formulations of clonidine, as part of this study, data on quality and stability of these new formulations will also be generated.

Methods/design

Study design

The CLOSED study is a multi-centre, double-blind, randomised, active-controlled non-inferiority trial with a 1:1 randomisation between clonidine and midazolam. Both treatment groups are stratified according to age in three groups with the same size: <28 days (n=100), 28 days to <2 years (n=100) and 2-18 years (n=100). The primary endpoint is defined as sedation failure within the study period.

Patient recruitment

Patients in five different EU member states (Czech Republic, Germany, Italy, The Netherlands and Sweden) will be recruited. Patients (neonates ≥34 gestational weeks and children <18

For peer review only

years) eligible for inclusion in the trial, according to in- and exclusion criteria, will be screened for possible enrolment (see Table 2 for inclusion- and exclusion criteria).

Table 2. Inclusion- and exclusion criteria for the CLOSED trial.

**Inclusion criteria**	**Exclusion criteria**
Aged from birth (GA ≥34 weeks) to <17 years, 11 months and one week	Body weight <1200 g or GA <34 weeks
(Expected) admission to the PICU	Body weight ≤3 kg AND age ≥28 days
	Body weight <10 kg AND age ≥2 years
	Body weight >85 kg
(Expected) indication for mechanical ventilation (both invasive and non-invasive)	Clonidine within last 7 days prior to admission*
	Sedation >72 hours prior to screening
Anticipated need for continuous sedation ≥24 hours	Known hypersensitivity to clonidine, midazolam, morphine or propofol or any of their formulation ingredients and their rescue medication
Informed consent (or deferred consent if applicable)	Administration of continuous muscle relaxants or other contra-indicated drugs
Informed assent if applicable	Post-resuscitation within 24 hours or therapeutic whole-body hypothermia
	CPAP or ECMO treatment
	Severe organ insufficiencies:<br>• Renal failure according to pRIFLE[36] or nRIFLE[37] criteria<br>• Cardiac failure as defined by modified Ross class 3 or 4<br>• Arterial hypotension according to guidelines[38]<br>• Circulatory failure as defined by Goldstein criteria[39]**
	Traumatic brain injury or other intracranial pathology including mental retardation and status epilepticus
	Phaeochromocytoma, acute asthma or paralytic ileus***
	Severe bradyarrhythmia

For peer review only

	Pregnancy<br>Known arterial hypertension in medical history<br>Previous participation in this trial at any time or previous participation in drug trial within last 3 weeks<br>Declined informed consent from parent(s)/legal guardian(s)

CPAP: continuous positive airway pressure; ECMO: extracorporeal membrane oxygenation; GA: Gestational age.

**)This exclusion criteria has been removed in the second protocol amendment*

***) These criteria have been modified in the second protocol amendment and allow for use of inotropes/vasopressor drugs*

****)The exclusion criteria acute asthma and paralytic ileus have been removed in the second protocol amendment*

Operating lists will be screened on a daily basis for possible patients expected to be admitted to the intensive care unit. These patients and parents will be identified, approached, informed and enrolled prior to surgery by study responsible doctors and/or nurses. Patients eligible for inclusion transferred to the centres will be identified by the transport team. Patients admitted to the PICUs will continuously be screened by PICU medical staff to identify patients possible for enrolment. Parents of these eligible patients will be approached, informed and enrolled by study responsible doctors and/or nurses.

Children are often admitted to the PICU/ NICU in an emergency setting. Urgent action will most likely be required and the subject's parent(s)/ legal guardian(s) may not be immediately available to give consent. An example of this is when a critically ill baby is delivered by Caesarean section; the mother may still be under anaesthesia while another parent/ legal guardian of the child may not be present. Therefore, given the nature of the trial, especially considering that ICU patients are often admitted as a result of emergency, deferred consent/assent, as described in Article 35 of the new European Regulation on clinical trials (EU) No 536/2014 of the European Parliament and of the Council[40], is foreseen if allowed by local

For peer review only

legislation, as described in section "Ethical considerations". This allows for the child to be enrolled in the trial before the parent(s)/ legal guardian(s) are able to give consent, which is then sought once they are available. If, subsequently, deferred consent is withheld, the subject is removed from the study but a record will remain in the subject's medical notes.

Study treatments

Firstly, it was necessary to define the dosing scheme so that appropriate drug concentrations could be used to maintain double blinding. A plasma clonidine concentration of around 2 mcg/L gave adequate sedation combined with morphine infusion in ventilated PICU patients[32], and 2 mcg/L clonidine concentration also drops the Bispectral Index to 71 in adults[41]. Clonidine has a long elimination half-life (16.9 h in neonates, 11.4 h in infants and 7.4 h in children[42]) so loading doses are proposed. It was therefore decided that loading and maintenance doses aimed at achieving steady-state concentrations of around 2 mcg/L would be aimed for, and this scheme was designed by a PKPD expert working group within the CLOSED consortium. Based on these doses, a similar dose scheme of midazolam was designed that would maintain double blinding. The agreed dosing scheme consists of 15-minute loading doses followed by a maintenance infusion rate. Increases or decreases, based on the evaluation of COMFORT-B and NISS score, in infusion rate are permitted, and preceding any increase, a loading dose over 15 minutes is specified. There are also compulsory lock-out times between each dose escalation or de-escalation to allow new steady-state conditions to be approached. Participants will follow the dosing regimen, with 1 unit meaning 1 microgram for clonidine, or 100 micrograms for midazolam:

- 1st bolus (T=0). Initial loading dose is 2 units/kg over 15 minutes and the following continuous infusion 1 unit/kg/h will be administrated to all patients followed by 15 minutes lock-out period.
- 2nd bolus. If insufficient sedation after the 1st lock-out period, the second loading dose is 2 units/kg over 15 minutes and the following continuous infusion 1 unit/kg/h will be administrated with a 15 minutes lock-out period.

For peer review only

• 3rd bolus. If insufficient sedation after the 2nd lock-out period, the third loading dose is 2 units/kg over 15 minutes and the following continuous infusion 1.5 unit/kg/h will be administered with a minimum of 105 minutes lock-out period.

• 4th bolus. If insufficient sedation after the 3rd lock-out period, the fourth loading dose is 2 units/kg over 15 minutes and the following continuous infusion 2 unit/kg/h will be administered followed by a 105 minutes lock-out period.

• 5th bolus. If insufficient sedation after the 4th lock-out period, the fourth loading dose is 2 units/kg over 15 minutes and the following continuous infusion 2 unit/kg/h will be administered with a 24 hours lock-out period.

• 6th to 11th bolus. If insufficient sedation after 24h lock-out period, the sixth to eleventh loading dose of 2 units/kg over 15 minutes and the following continuous infusion 2 unit/kg/h would be administered with at least 24 hours lock-out period between.

All doses are halved in patients younger than 1 month. Figure 1 shows the scenario of all loading doses of study drug administered for subjects.

[Insert Figure 1 here].

Decreasing the maintenance dose will be possible in the case of oversedation (i.e. COMFORT-B>22 OR COMFORT-B=11-22 and NISS=3).

Dose decrease will take the following steps (doses are halved in neonates):

- 2 units/kg/h will be decreased to 1.5 units/kg/h
- 1.5 units/kg/h will be decreased to 1 units/kg/h
- 1 units/kg/h will be decreased to 0.75 units/kg/h
- 0.75 units/kg/h will be decreased to 0.5 units/kg/h

If patients are still oversedated with the minimum dose, the IMP administration will temporarily be stopped until patients need sedation again, and they will start back at T=0.

During the preparation of the trial, one unknown challenge has faced the consortium already. In version 1.0 of the protocol, dose reduction should be performed when a child was oversedated.

For peer review only

After 30 minutes, a new sedation assessment should be performed and acted upon the same. In an oversedated child, this could lead to a child being without IMP after 90 minutes (3 dose reductions). This is certainly not a desirable situation, but not unthinkable as the half-life of both drugs are longer than 90 minutes. Therefore, a six-hour observation period has been implemented. After a dose reduction, it is recommended to wait for 6 hours before the next dose reduction.

In this same amendment, another problem has been mitigated. Version 1.0 did not provide clear information about increasing the dose again after a prior decrease. In the amendment, it has been made clear that subjects having a decrease of maintenance infusion to <1 unit/kg/hr, will return to 1 unit/kg/hr, irrespective of the current infusion rate. If the decrease has been made while increases before have led to a maintenance infusion rate of ≥1 unit/kg/hr, re-increase will be following the precedent step (for example: if a decrease has taken place from 1.5 to 1 unit/kg/hr, re-increasing will return the rate back to 1.5 unit/kg/hr).

Pharmaceutical development

To overcome the potential for administration errors associated with commercially available clonidine ampoules, three different strengths of clonidine HCl – low (250 mcg/50ml), medium (500 mcg/50ml) and high (2500 mcg/50ml) – have been developed. Based on the dosing regimen described above, the concentrations of the low, medium and high strengths of midazolam (25 mg/50ml, 50 mg/50ml and 250 mg/50ml) were 100-fold higher than for clonidine HCl. For study blinding purposes and to avoid dosing errors, a simple 3 colour scheme based on strength will be used.

To avoid the administration of preservatives, all formulations will be stored in single-use glass vials, with any contents remaining after 24 hours to be discarded. A 50ml volume has been selected to limit the number of children requiring more than one vial in 24 hours (only subjects weighing over 46 kg and receiving the maximum dose in 24 hours will require two vials in a 24-hour period).

For peer review only

Efficacy endpoints

Level of sedation will be evaluated using the COMFORT-B score (ranging from 6 to 30) which is a validated scoring system commonly used in the PICU[43]. Level of sedation will also be evaluated by the Nurses Interpretation of Sedation Score (NISS) when the COMFORT-B score falls between ≥11and ≤22. The NISS is the nurse's expert opinion of the level of sedation (1: insufficient sedation; 2: adequate sedation; 3: oversedation). A subject is considered to be under-sedated in cases of COMFORT-B score of >22; or COMFORT- B score of 11–22 in combination with NISS of 1.

In addition to COMFORT-B Score (and NISS where appropriate), pain will be assessed using the Numerical Rating Scale (NRS) at the same time intervals. The 11-point NRS is a global pain rating scale with which the nurse rates pain intensity by number (0 = no pain and 10 = worst imaginable pain)[44]. In this study, to simplify matters, the NRS score will be addressed first, so, if the pain score is ≥ 4, morphine treatment will be escalated whilst sedation treatment will remain the same, regardless of the COMFORT-B score. This means that any pain score will take precedence over the sedation score.

Primary objective

To assess the non-inferiority of the sedative properties of continuous IV clonidine compared to continuous IV midazolam in mechanically ventilated children and adolescents (0-18 years) admitted to a PICU.

Primary Endpoint

The primary endpoint is defined as sedation failure within the study treatment period (a maximum of seven days).

Sedation failure is defined as:

When a subject's assessment results are:

Numerical Rating Scale (NRS) score <4 and COMFORT B score >22

OR

For peer review only

Numerical Rating Scale (NRS) score <4 and COMFORT B score ≥11and ≤22 AND Nurses' Interpretation of Sedation (NISS) score 1 at a point during the study where no further increase in IMP dose are permitted as described in the dose escalation scheme.

In summary, there are two possible outcomes (success or failure) of the primary endpoint.

Secondary objectives

To evaluate the safety and tolerability (including withdrawal effects) of clonidine compared with midazolam in ventilated children and adolescents admitted to PICU.

To determine clonidine dose-dependent effects on sedation.

To establish the PK-PD relationship of clonidine for sedation in PICU.

To compare the cumulative total morphine consumption/kg between the two arms in the first 48 hours of IMP administration.

Secondary endpoints

Primary PK parameters estimated will be clearance (CL), volume of distribution (VD) and inter-compartmental clearance (Q). PK measurements will be made using sparse opportunistic sampling.

PK-PD modelling will seek to elucidate the relationship between IMP pharmacokinetics and sedation as measured by COMFORT-B score.

The PK-PD covariate model will include demographics (e.g. age, weight), clinical characteristics (e.g. reason for admission) and pharmacogenomics (see Genetic parameters section).

Safety and tolerability assessments

Safety and tolerability assessments include:

- Extent of withdrawal effects using the Sophia Observation withdrawal Symptoms-Paediatric Delirium (SOS-PD) scale measured three times a day in subjects who receive sedatives and/or opioids for 5 days or more and after cessation of treatment in all

For peer review only

subjects for at least 24 hours after treatment. The extent of delirium measured by the Sophia Observation withdrawal Symptoms- Paediatric Delirium (SOS-PD) scale[45].

- Rebound hypertension monitored for at least 72 hours post cessation of treatment.
- Percentage of respiratory depression per group.
- Adverse event reporting of symptoms indicative of post-ICU stress (e.g. nightmares, confusion, hallucinations).
- Neurodevelopment of subjects recruited in lower age group (from birth to 27 days) at 12 months after cessation of IMP, as measured using the Bayley II Score[46].

Effect size justification

The primary objective of this study is to evaluate the efficacy of clonidine compared to midazolam in the sedation of ventilated children and adolescents admitted to PICU, with a view to demonstrating non-inferiority of clonidine.

Every patient enrolled will be in need of sedation, thus a placebo cannot be considered. Intravenous midazolam is the standard treatment for longer-term sedation in children. It is licensed in this population, regarded as standard of care, and recommended by treatment guidelines[12, 30].

Based on clinical experience and the limited data available regarding the use of clonidine as a sedative agent in the PICU, it is assumed that the sedation success rate for clonidine is higher than for midazolam. The estimated difference in success rate between the two drugs was assumed to be 5%.

The primary endpoint is the success or failure of the subject's sedation treatment. Failure is defined as inadequate sedation with no further dose increases allowed in the dose titration scheme. This will be assessed by a nurse or physician using the COMFORT-B sedation assessment and, if appropriate, Nurse's Interpretation of Sedation Score (NISS). These assessment scales are open to a degree of subjectivity and it is therefore considered essential to

For peer review only

have a double-blind study design to minimise bias due to knowledge of treatment group by the assessor.

In order to define the effect size the expected success rate for midazolam was needed. The target concentration for midazolam was taken from the de Wildt et al 2005 paper showing around 311 mcg/L is required for sedation[47]. The maximum midazolam dose of 200 (100-200) mcg/kg bolus followed by 200 (50-200) mcg/kg/h for all age groups (including neonates) is recommended by Dutch clinical consensus guideline [48]. According to the proposed dosing schedule, the exposure of midazolam is similar to the Dutch recommended dosing regimen from the simulation (Figure 2). Furthermore, after the 5th bolus according to the proposed dose scheme, the proportion of average midazolam plasma concentration above the target value (0.311 mg/l) is 74% and from Dutch dose scheme is 72%. Thus, we assumed that the sedation success rate in the midazolam arm given our dosing scheme would be 75%, and in the clonidine group this would be 80%.

[Insert figure 2 here]

Sample size calculation

Given this assumed effect size of a 75% success rate for midazolam, a sample size of 258 (129 per treatment arm) provides at least 80% power to show non-inferiority of clonidine compared to midazolam. The sample size calculation is based on a logistic regression for the primary efficacy endpoint of sedation success, with adjustment for study arm, age category, and baseline COMFORT-B score. Non-inferiority of clonidine requires that the lower limit of the one-sided 97.5% confidence interval for the odds ratio of sedation success using clonidine (versus midazolam) is at least 0.583, which is equivalent to a 10% non-inferiority margin in case of a sedation success rate of 80%. The required sample size was calculated using simulation.

It is assumed that the total drop-out rate will be less than 13% and hence 300 subjects will be recruited, 150 in each arm and 100 in each age subgroup. The sample size of 300 subjects also

For peer review only

provides at least 70% power to prove non-inferiority with respect to sedation success rate for each age subgroup (>90 subjects per age subgroup). Since the sample size for the age subgroup analyses is limited, a 15% non-inferiority margin at a one-sided alpha level of 0.025 is used, with an assumed sedation success rate of 80% in the midazolam group and 85% in the clonidine group. Subgroup analyses will be performed using a confidence interval for the Mantel-Haenszel risk difference, with stratification by centre.

Statistical analysis

Analysis Populations

The Safety Evaluation Set (SES) is the subset of all subjects who were randomised into the trial and exposed to study medication (also referred to as the Intention To Treat (or ITT) population). Safety analyses will be performed in the SES.

The Full Analysis Set (FAS) is the subset of subjects in the SES for whom the primary efficacy variable is available. Data analysis for the efficacy endpoints will be performed in the FAS.

The Per Protocol Set (PPS) is the subset of subjects in the FAS without major protocol deviations. Major protocol deviations will be defined during the Blinded Data Review Meeting (BDRM).

The Pharmacokinetic/Pharmacodynamic Analysis Set (PKS) is the subset of subjects in the SES with evaluable PK samples defined as drug concentration measurements.

Primary Efficacy Analysis

Logistic regression of the primary endpoint, sedation failure, with treatment group, baseline sedation assessment (i.e. the baseline COMFORT-B score), centre and age group as covariates, at a one-sided significance level of alpha = 2.5% will be used. The statistical hypotheses are the following:

For peer review only

$H0: OR <= \delta_{OR}$ and $H1: OR > \delta_{OR}$,

$OR = p_C*(1-p_M)/((1-p_C)*p_M)$

where p_C and p_M are probabilities of sedation success in the clonidine group and midazolam group, respectively. δ_{OR} is the non-inferiority margin which is predefined as 0.583.

H0 will be rejected if the lower bound of the one-sided 97.5% Wald confidence interval (CI) for the odds ratio of sedation success proportion will be greater than 0.583. The primary analysis will be based on the ITT principle. The primary endpoint will be evaluated for the Full Analysis Set. An equivalent analysis for the per protocol set will be performed as a sensitive analysis. No interim analyses are planned for the primary endpoint

Secondary Efficacy Analyses

All secondary efficacy analyses will be conducted on the Full Analysis Set. Secondary efficacy endpoints will be analysed in detail as follows:

- For the proportion of subjects with sedation success between treatments within each age group will be based on the confidence interval for the Mantel–Haenszel risk difference) with stratification by centre.[49] A 15% non-inferiority margin at a one-sided alpha level of 0.025 will be used.
- The aggregate intervals (i.e. the total time) (based on the planned assessment points) with COMFORT-B Score >22 or <11 during treatment period between treatment groups will be analysed using the Mann-Whitney test.
- The proportion of subjects with COMFORT-B Score ≤11 between treatment groups and within each age group will be analysed using the chi-square test or Fisher's exact test.
- For the morphine consumption in units of mcg/kg/day during the first 24 hours of IMP administration and for the remaining study period between treatment groups will be analysed using linear regression analysis, with adjustment for centre and age group. An

For peer review only

appropriate transformation of morphine consumption may be used to ensure normality of the residuals in the linear regression model.

An age subgroup analysis will be performed for each age group, using centre as a stratification variable.

Pharmacokinetic/pharmacodynamic analyses

The primary objective of pharmacokinetic/pharmacodynamic analyses in this trial is to evaluate the pattern and extent of covariates affecting the PK/PD profiles of clonidine and to provide the information of optimal dose for patients based on their particular age and other related covariates for future clinical practice. Three PK-PD interim analyses will be undertaken whereby samples will be shipped and assayed for drug concentrations in order to ensure the systems for sample transport and analysis, along with data linkage to the eCRF are fully operational, and to allow preliminary PK-PD model development. PK-PD interim analyses will be conducted after 15, 100 and 200 subjects completed the study.

PK/PD data will be modelled using the Fortran-based non-linear mixed effects software NONMEM. Typical PK dispositional compartmental models will be tested, along with investigation linear and non-linear elimination. Multivariate covariate analysis will be undertaken to investigate the impact of subject factors on PK model parameters. In particular we will focus on the effect of body size with allometric models, and age (both postmenstrual and postnatal) possibly using literature prior models in order to delineate the effect of size and age from other factors. We will also look at the impact of drug metabolising enzyme genotype on inter-individual variability. The link between PK and PD (COMFORT-B score) will be investigated by sequential and simultaneous modelling, possibly including an effect compartment. The Item Response Theory (IRT) will also be considered for PK-PD modelling, whereby effect is considered an unobserved normally distributed latent variable, and each item score of COMFORT-B is allowed to contribute to this through link functions[50]. Since subjects

For peer review only

will receive other sedative agents for procedures, the concentrations of which may not be measured, a PK-PD approach will be taken to model sedation requirements in this period.

Mixed effects models will be fitted with maximum likelihood and addition of a single fixed effect will be guided by improvement of fit using the likelihood ratio test. Model evaluation will consist of goodness-of-fit (residual plots) and simulation-based diagnostics (visual predictive check), and parameter precision and robustness will be investigated with non-parametric bootstrapping.

Safety Analyses

All safety analyses will be performed on the SES. Analyses will include the following endpoints:

- AEs, SAEs and SUSARs.
- Deaths
- Clinical laboratory evaluations and vital signs. Descriptive summaries of laboratory values (including clinical chemistry, haematology, coagulation and urinalysis variables) and changes from baseline throughout the study will be generated. Shift tables will also be used for comparing changes from baseline and the proportion of subjects experiencing abnormalities between treatment groups
- Sophia Observation withdrawal Symptoms-scale (SOS). The SOS scores will be descriptively summarised and analysed with a linear mixed model (with between-arm differences at baseline constrained to be 0).
- Neurodevelopment will be evaluated using the Bayley Scales of Infant and Toddler Development. The scale will be descriptively summarised and compared between treatment groups using linear regression analysis, with adjustment for centre and age group.

For peer review only

Quantitative variables will be summarized by the number of subjects analysed (N), mean, standard deviation (SD), median, minimum, and maximum. Categorical variables will be summarized by frequencies and percentages for each category.

Randomisation and kit assignment

Randomisation is stratified by both age subset and clinical site. Each stratum consists of 99 pre-randomised numbers (randomisation numbers), assigned to either clonidine or midazolam. These randomisation lists were prepared by a statistician not otherwise involved in the study, with the use of blocked randomisation consisting of small variable block sizes, in order to maintain blinding and conceal allocation.

At each clinical site, the kits marked with the random numbers for each age subset will be assigned to subjects in the order they are enrolled into the study. Each subject kit will contain two IMP boxes of seven vials, in order to supply the subject with enough medication for the maximum seven-day study period. The contents of each subject kit is outlined in Table 3.

Table 3. Composition of the subject kit for each age group

Age Subset	Age group	Formulation strength	Subject bodyweight	Number of boxes
1	<28 days	Low	≤3 kg	1
		Medium	>3 to <10 kg	1
2	28 days to <2 years	Medium	>3 to <10 kg	1
		High	≥10 kg to <47 kg	1
3	≥2 years to <18 years	High	≥10 kg to 85 kg	2 (in case ≥47 kg)

Emergency unblinding will be possible as for each randomised subject kit, two complete sets of sealed emergency envelopes have been prepared (one set for safekeeping by the investigator,

For peer review only

the other will be delivered to the group responsible for overseeing pharmacovigilance of the study). Unblinding of the treatment allocation for a subject will be performed in an emergency that, in the opinion of the investigator, is warranted for a given subject for safety reasons.

Pharmacokinetic/pharmacodynamic endpoints

Pharmacokinetic/pharmacodynamic (PK-PD) analyses will be carried out as a secondary endpoint to give further information on the dose-concentration-effect relationship primarily of clonidine, but also of midazolam. The PK-PD model will be used to evaluate the dose scheme used in the study, and if necessary recommend an updated dose scheme for clinical use. PK/PD data will be modelled using nonlinear mixed effects modelling. The impact of drug metabolising enzyme genotype on inter-individual PK-PD variability will be considered alongside other demographics in the multivariable covariate analysis. The link between PK and PD (COMFORT-B score) will be investigated by sequential and simultaneous modelling, possibly including an effect compartment. Item Response Theory (IRT) will be explored for PK-PD modelling, whereby effect is considered an unobserved normally distributed latent variable, and each item score of COMFORT-B is allowed to contribute to this through link functions[50].

Mixed effects models will be fitted with maximum likelihood and addition of a single fixed effect will be guided by improvement of fit using the likelihood ratio test (change in objective function of 3.84 is significant at p=0.05 level for 1 degree of freedom by the chi-square distribution).

Model evaluation will consist of goodness-of-fit (residual plots) and simulation-based diagnostics (visual predictive check), and parameter precision and robustness will be investigated with non-parametric bootstrapping.

For peer review only

Pharmacogenomic endpoints

Patients treated with sedatives and analgesics may respond very differently to the same dosage of medication. Several gene polymorphisms have been discovered to influence the pharmacokinetics and pharmacodynamics of clonidine, midazolam and morphine. PK data

collected in this study can be interpreted more completely if data on pharmacogenomics are available.

Moreover, to study candidate polymorphisms gives further insight into the response of critically ill children to clonidine, midazolam, and morphine. Most candidate genes have been established in adult subjects, this study is an opportunity to study the effects in children. The candidate genes which will be genotyped are shown in Table 4.

Table 4. Candidate genes for linking pharmacogenomics to pharmacokinetic and pharmacodynamics endpoints.

Midazolam	Clonidine	Morphine
CYP3A4, CYP3A5, POR, ABCB1, GABA, MDR1, MRP1, MRP2, MRP4, BRCP.	ADRA2A, CYP2D6.	COMT, OPRM1, OCT1, UGT2B7, ABCC3, MC1R, IL-1Ra, IL-

Ethical considerations

Ad hoc considerations and measures have been set up to ensure safe and ethical conduct of this multi-national trial, including a population requiring special protection such as children. In order to guarantee the respect of ethical rules regardless of the country in which the trial is carried out, an ethical standard based on the EU ethical and legal framework has been agreed among centres. All submissions are based on the current European ethical and legal framework and on the international ethical principles and guidelines.

Based on the Council of Europe Convention on Human Rights and Biomedicine[51] and EU Ethical Recommendations 2008[52] research involving vulnerable populations is allowed if the results of the research are expected to provide real and direct benefit to the health of the patient, or some benefit for the population represented by the patient, and the trial will pose only minimal risk and burden to the individual concerned. The risk-benefit assessment has been carefully addressed considering IMP related risks (including pharmacologic properties and

For peer review only

proposed dosing regimen of the IMP, information on use in target population, IMP development, IMP management) and expected benefits, the trial design (including study population inclusion and exclusion criteria), study related procedures, qualification of the study team and host sites, rights of patients (including informed consent and assent procedures and data protection and confidentiality). Minor increase over minimal risk is anticipated for the CLOSED trial, as the study deals with a novel treatment modality in vulnerable paediatric patients and carries minor risk based on the available information about the study drug as well as RCT related procedures. Important benefits for PICU population as a group are anticipated, since the study will provide clinically relevant and directly applicable results, expected to influence future standard of care. The informed consent and assent process in this trial is in line with the applicable relevant regulatory documents, including Good Clinical Practice (GCP) guideline[53], Directive 2001/20/EC[54], the EC Detailed Guidance 2006[55], Directive 95/46/EC[56] , the International Ethical Guidelines for Biomedical Research Involving Human Subjects CIOMS-WHO (2002)[57], the Additional Protocol to the Oviedo Convention (2005)[58], ICH Topic E11, and the EC 2008 Paediatric Recommendations. Accordingly, clear and appropriate information sheets and informed consent forms for parents/legal representatives, and patient information sheets and assent forms are prepared for patients and customised according to the local requirements. Given the nature of the trial, especially considering that ICU patients are often admitted as a result of emergency, deferred consent/assent, as described in Article 35 of the new European Regulation on clinical trials (EU) No 536/2014 of the European Parliament and of the Council[40], is foreseen if allowed by local legislation. Informed consent of the legal representative and, if feasible, the patient's assent will be sought for as soon as possible. If informed consent is not obtained, the possibility of withdrawing all collected data from the trial will be explained and appropriate measures, based on patient's decision will be taken. A record will remain in the subject's medical notes. In The Netherlands, deferred consent is permitted under national legislation ('Wet medisch-wetenschappelijk onderzoek met mensen', Paragraph 2 Article 6.4.).

For peer review only

As the protocol includes pharmacogenetic tests, informed consent and assent to perform genetic tests will be obtained separately, in line with the most relevant provisions in the field (International Declaration on Human Genetic Data, UNESCO, 2003[59]; Ethical Guidelines CIOMS-WHO, 2002). Accordingly, the participants will have the possibility to join the trial without participating in the pharmacogenetic part.

Finally, involvement of appropriate external expertise in the form of Data Safety Monitoring Board (DSMB), Independent Scientific Advisory Board (ISAB) and Patient Advisory Board (PAB) is foreseen. In details, the DSMB will review the study's safety data and assess subject safety data throughout the study; the ISAB will input in the final protocols, monitor the progress of the project and ensure the on-going scientific and ethical integrity of the clinical trial; the PAB will advise the Sponsor to ensure that the subjects' rights and subjects' protection will outweigh any commercial considerations and conflicts of interest that may appear in relation to the project.

In accordance with GCP, as implemented at national level, the study protocol and related documents have been approved by the competent ethics committees in Rotterdam, the Netherlands, and submission is currently being prepared for the four other centres (Stockholm, Prague, Erlangen and Rome).

Dissemination

The results will be published in a peer-reviewed journal and shared with the worldwide medical community.

Discussion

Study design

Sedation management in the PICU mainly consists of the use of IV midazolam by continuous infusion. Midazolam has several advantages, but is by far not the ideal agent for longer-term

For peer review only

sedation. Other agents have made their off-label entry into the PICU, mainly based on adult practice. One of these agents is clonidine which could have some specific advantages over midazolam. It is being used increasingly, but is still off-label. This study design allows for data collection on safety, efficacy, PK, pharmacogenomics and to a lesser extent neurological outcome. This multimodal approach generates data that is very useful for licensing clonidine for sedation in children. Moreover, application of different formulation strengths, allows for easier paediatric clinical implementation as the current available formulation (Catapresan®) has only been developed for adults.

Study limitations

Despite the careful design of this trial, some limitations exist. First, we have chosen a non-inferiority design for this trial because, based on clinical experience and limited available data, we estimate the difference in sedation success rate will be very low. This would mean that showing superiority of clonidine requires a large sample size and thus compromising feasibility. This design, however, means that we cannot demonstrate possible superiority of clonidine. However, if equal efficacy is shown under safe circumstances, clonidine can be licensed for sedation in the PICU.

Second, the recruitment window has been set on 72 hours. This could lead to confounding as having a large time window could increase the risk of tolerance and withdrawal symptoms. A smaller recruitment window may reduce these risks but parents need some time to consider participation and not every study site has 24/7 research nurse availability.

Third, this trial has an extensive list of exclusion criteria. This could compromise the external validity of the results and could complicate implementation of the results in new guidelines. This is a known limitation of almost any randomized controlled trial[60]. However, these exclusion criteria are based on expected elements affecting both primary outcome and patient safety. Our primary endpoint is based on a validated behavioural scale. Therefore, any disease status that can cause many fluctuations in behaviour (such as neurological injury) needed to be excluded

For peer review only

from this trial for the results to be valid. The same holds for safety endpoints such as cardiovascular stability. The decision to allow for inotropes/vasopressors in the second amendment is a big step towards generalisability of the results.

Role of researcher-driven studies, Seventh Framework Programme-funded projects

CLOSED is one of the 20 projects approved in the framework of the Seventh Framework Programme for Research that should specifically meet both criteria for scientific excellence and regulatory standards for high quality paediatric research, as prescribed by the Paediatric Regulation, in order to put paediatric drugs on the market. A very recent publication demonstrated that these projects face the need for overcoming the existing methodological and ethical difficulties affecting research in the paediatric population [5].

In fact, paediatric clinical trials are often multi-national and researchers need to know and apply rules from the regulatory, legal and ethical frameworks, acting both as investigators and as sponsor and/or other concerned parties. This is not simple considering the lack of harmonisation of clinical trial procedures among countries [http://www.ncbi.nlm.nih.gov/pubmed/26037896] and makes it necessary to prepare, agree and apply guidelines based on the EU rules.

Challenge to perform a trial compliant with GCP and other regulations

Clinical trials must follow the same rules, notwithstanding if sponsored by industry or non-profit organisations, from GCP to the new clinical trials regulation [53] to recommendations and guidelines.

Accordingly, the new regulation [53] recognises that a large proportion of clinical trials are conducted by non-commercial sponsors, frequently supported by public funds or charities and that these trials should be encouraged.

However, this means a heavy work from researchers and a deep knowledge of both EU and national rules. This issue is aimed to be overcome by the international public-private

For peer review only

cooperation between different stakeholders foreseen by EU-funded projects (researchers, clinicians, regulatory and ethical experts, clinical research associates). On the other hand, it seems that the paediatric Consortia generated by the FP7 paediatric projects are conducting these studies and trials using a limited amount of money in comparison with the recognised cost of paediatric trials in an approved PIP which is estimated to be three to four times higher[5].

Challenges of maintaining double-blinding

Clonidine and midazolam have different half-lives: 1-3 hours for midazolam (up to 12h in neonates) and 9-17 hours for clonidine, thus maintaining double-blinding is challenging. PK-PD simulations have played a major role in the strategy to overcome this challenge. It has led to the implementation of a loading dose, which is in line with the current clinical recommendations for midazolam[48]. Target plasma levels have been achieved based on the literature[32, 47]. It should be noted, however, that the PK-PD relationship is assumed, but not formally studied. This study will contribute to the determination of a PK-PD relationship.

Risk to patients

Both clonidine and midazolam are frequently used sedatives in the PICU. However, a careful assessment of possible study-related risks has been performed. In general, side effects of IMP are the major cause of study-related risk. As midazolam is the gold standard treatment, the side effects do not contribute to an increased risk in this study. For clonidine, bradycardia and hypotension are the most clinically relevant side effects and are likely to occur most often during the loading doses and the early starting phase[33]. Therefore, a loading dose will be given as a 15- minute infusion instead of a direct bolus. Moreover, the setting where patients are being monitored consists of five well-equipped and experienced PICUs. Treatment of hypotension and/or bradycardia is common practice in the study population and should therefore not increase the risk to subjects dramatically.

Rebound hypertension could also be a side effect after sudden discontinuation of clonidine. In a comparable trial,[31] this was observed once in 64 patients and resolved quickly without any

For peer review only

intervention. Monitoring of blood pressure up to 72 hours after IMP cessation has been included in the study protocol with the recommendation to treat hypertension according to local common practice.

Blood sampling is a possible risk to small children participating in drug research.[61] In this study, the amount of sampled blood will not exceed the 3% of patient's plasma volume, which is a general limit of study-related blood sampling.

In general, as most elements of this study are similar to current clinical practice, the study-related risk to patients is deemed low.

Pharmacogenetics

This study incorporates a pharmacogenetics assessment of recruited patients. Both PK and PD may be influenced by gene polymorphisms. The current proposed pharmacogenetics assessment has been based on the literature available, but a degree of flexibility has been built in when more information on these genes or medications will come available.

In accordance to the appropriate regulations,[55, 57, 59] separate consent and assent will be obtained from participants. This strategy enables the possibility of participating in the CLOSED trial without the genetic assessment.

Anticipated difficulties

A comparable RCT, the SLEEPS study, has been previously undertaken in the United Kingdom[33]. One of the major challenges faced by the investigators was the recruitment. The sample size had been calculated on 1000 subjects. Unfortunately, only 129 subjects were randomised. Contributing factors to this impaired recruitment have been identified by the investigators, including:

- Conflict with other studies
- Earlier extubation in elective cardiac surgery patients
- Parental issues and timing of consent
- Clinicians' issues

For peer review only

- Research nurse time
- Delay in study start
- Compliance with the protocol.

It is likely that in the participating centres with excellent research facilities, multiple trials are being performed at the same time. Children are not allowed to participate in multiple intervention studies, so we recommend to have a thorough look at the in- and exclusion criteria of studies and set up clear agreements about patient allocation.

Early extubation in cardiac surgery patients is now common practice, and estimations of recruitment rates have excluded these patients. However, it is likely that ventilation strategies will change over the coming years. We have recognised the increasing use of non-invasive ventilation such as Non-Invasive Positive Pressure Ventilation (NIPPV). As these patients may still need continuous sedation, we will include these in the study. In general, patients on Continuous Positive Airway Pressure (CPAP) do not need additional sedation, thus these will be excluded.

Parental issues are to be expected in any clinical trial in the PICU. Parents having their child admitted to an ICU are under high stress and may therefore be reluctant to participate. In the calculations of estimated recruitment, a high refusal rate (approximately 40-50%) should be taken into account, based upon earlier experience with other trials. Also, a parent organisation is involved in the trial and they will provide information to parents from their perspective. We anticipated to include 300 patients within 18-24 months in total. In The Netherlands, deferred consent is used in emergency care trials. The Ethical Committee of the Erasmus Medical Centre, Rotterdam, has approved the use of deferred consent in this trial so this could be applied in the largest recruitment centre.

When faced with a critically ill child, doctors and nurses may tend towards conservatism. At the beginning of the SLEEPS trial, the involved health care professionals could have been afraid of the cardiovascular side effects of clonidine especially in unstable patients. Their study has shown that its use is relatively safe in critically ill children, as well as other literature.[34, 62]

For peer review only

Moreover, in the CLOSED consortium recruitment centres, a lot of clinical experience with clonidine has been built so caregivers may be less hesitant to support this clinical trial. Research nurse time is always an issue, especially in the 24/7-business of an ICU. In the larger centres, dedicated researchers/nurses have been identified and trained adequately to perform this trial. In the smaller centres, PIs may be able to coordinate the lower amounts of participants better.

During the first months of patient recruitment in two centres, no patients could be included. In the majority of cases, exclusion criteria made the screened patients ineligible. No consent was given in the very few eligible patients. Therefore, a revision of the exclusion criteria have been made.

The major exclusion criteria on which patients failed screening are:

- **Anticipated need for sedation <24 hours**. It has been considered to change this duration. It has been decided however to keep this minimum as the therapeutic aim of the study is long-term sedation.
- **No sedation despite ventilation.** To our surprise, many newborn patients on the ventilator would not need any sedation, or were comfortable on low-dose opioids. This could not be mitigated.
- **Circulatory insufficiency.** The Goldstein criteria[39] have been used for the definition of this exclusion criterion. However, many patients received inotropic or vasopressor agents as supportive therapy in both recruiting centres. We have therefore modified these criteria and removed the use of inotropes/vasopressors in the second protocol amendment.
- **Neurological pathology.** It has been suggested to include patients with minor head trauma, but as the clinical picture may change very rapidly, we have decided not to include these patients into the trial.
- **Use of clonidine in the last 7 days.** As clonidine has a long half-life, patients would be excluded if they have received clonidine for any indication in the previous 7 days.

For peer review only

However, for midazolam, there was no similar exclusion criterion; instead, patients would undergo an extra PK blood sample before start of the study medication. This has now been introduced for clonidine as well in the second protocol amendment.

A delay in study start has been encountered in the CLOSED study. Several obstacles, such as obtaining the appropriate licenses for handling, importing and exporting controlled drugs, have caused a significant delay in recruitment start. A second major issue has also been an amendment of the dose reduction scheme (see paragraph 'Study treatments'). A third issue has been the generation of robust quality data that is sensitive enough to support a future PUMA application as well as the IMPD and clinical trial application [63].

Compliance with the protocol was a significant issue in the SLEEPS trial, as they provided a strict sedation regimen. Therefore, the CLOSED sedation regimen merely reflects clinical practice, although some important aspects may cause difficulties in clinical practice. The lock-out periods for example, are not according to clinical practice and special attention needs to be paid by the research nurses and other investigators to keep the protocol compliance as tight as possible. When problems are recognised, the possibility of amendments should be discussed early. Early recognition is facilitated by implemented monthly investigators' teleconferences.

Also, there will be an ongoing evaluation of the COMFORT-B scale training of the nurses in the participating centres. Good inter-observer reliability is defined by a Cohen's kappa >0.65 and nurses in all centres have received training by trained nurses.

In summary, many known challenges should be managed by appropriate measures taken. However, there could always be unknown challenges and it is therefore crucial to have regular contact between principal investigators of each recruitment site.

For peer review only

Reflection

After 9 months of recruiting, we included far fewer children than anticipated. Therefore, two amendments have now been added to potentially increase the number of eligible patients. Unfortunately, recruitment did not improve. Even though we had been warned by the early discontinuation of the SLEEPS trial[33], we faced other challenges. For example, many postoperative neonates do not need any additional sedation to IV paracetamol and continuous IV morphine. Also, parents are reluctant to participate. Either they think their child is not stable enough, or their child is finally stable and therefore changes in treatment for study purposes are not welcomed.

Also, the anticipated recruitment rate was based on the number of admitted ventilated patients in previous years. However, this turned out to be a significant over-estimation, a phenomenon also known as Lasagna's Law[64]. These challenges have a big impact on the feasibility of the trial and force us to consider alternative options. We will open (at least) two new recruitment sites, Bari (Italy) and Tallinn (Estonia). Furthermore, we need to rethink the primary objective of this study and may change it to a PK-PD study for which 50 patients in each arm are sufficient instead of the original 150 patients per arm.

The lessons learned from both the SLEEPS trial and this trial are important for further investigations in paediatric critical care and careful preparation is warranted for any trial performed in this population. This preparation should at least include adequate piloting over a longer period. In our experience, we monitored eligible patients over one month in one centre and expected no significant recruitment problems. This monitoring was performed during winter time, when many patients with respiratory viral infections in need of mechanical ventilation were admitted. This caused our expectations of eligible patients to be high; if we had performed this during summer time, we may have been warned earlier.

For peer review only

Other aspects to enhance the number of included patients are adequate staff training and motivation, collaboration with investigators having experience in paediatric critical care trials, and keeping the exclusion criteria of a trial to a minimum.

Trial status

The trial started enrolling subjects in the summer of 2016 in Rotterdam and Erlangen, where ethical approval is available. To date, 5 patients are included. Recruitment in other sites will follow when ethical approval becomes available. The second amendment including changes of the exclusion criteria have been submitted to the ethical and regulatory institutions and approval has been obtained in Rotterdam and Erlangen. The current study protocol, including the second amendment, is version 3.0, dated 21st October 2016. In Rome, Prague and Stockholm, approval has only been given for protocol version 2.0, dated 22nd February 2016. Approval for version 3.0 is expected in the first half of 2017.

Author contributions

AN: Coordinator of the trial and initiator of the study. Collaborated on writing this manuscript.

MB: Coordinating researcher in main trial site, contributed specifically to the pharmacogenomics and the NIMP section of the protocol. Collaborated on writing this manuscript. Responsible for data collection.

MvD: Collaborated on establishment of the primary and secondary endpoints of this trial.

JvR: Main biostatistician responsible for the sample size calculation and statistical analyses.

JS: Collaborated on statistical analyses and performed PK modelling.

YS: Collaborated on the statistical analyses and performed PK modelling.

For peer review only

WR: Collaborated on the protocol and amendments.

DR: Responsible for compiling the sections of the protocol into one document, collaborated on pharmaceutical development.

JW: Collaborated on the regulatory section.

LR: Collaborated on the ethics and regulatory section.

SH: Responsible for pharmaceutical development.

TM: Responsible for the laboratory analyses and sampling plan.

VG: Responsible for ethics and regulatory sections of the protocol.

PL: Collaborated on trial design and study population. Principal Investigator at Swedish site. Responsible for data collection.

PP: Collaborated on trial design and study population. Principal Investigator at Czech site. Responsible for data collection.

AS: Collaborated on trial design and study population. Responsible for data collection.

DT: Principal Investigator and initiator of this study. Collaborated on trial design.

Competing interests

None declared.

Data sharing statement

Not applicable: as this is a study protocol.

For peer review only

Funding and sponsor

This project has received funding from the European Union's Seventh Framework Programme for research, technological development and demonstration under grant agreement n.602453. This funding source had no role in the design of this study and will not have any role during its execution, analyses, interpretation of the data, or decision to submit results.

The sponsor is:

Universitätsklinikum Erlangen represented by the Dean

Medizinische Fakultät

Friedrich-Alexander-Universität Erlangen-Nürnberg

Maximiliansplatz 2

91054 Erlangen

Telephone: +49 9131 85 47047

Telefax: +49 9131 85 35120

For peer review only

References

1. Ceci, A., et al., *Medicines for children licensed by the European Medicines Agency (EMEA): the balance after 10 years.* Eur J Clin Pharmacol, 2006. **62**(11): p. 947-52.
2. Neubert, A., et al., *Defining off-label and unlicensed use of medicines for children: results of a Delphi survey.* Pharmacol Res, 2008. **58**(5-6): p. 316-22.
3. European Medicines Agency, *Report on the survey of all paediatric uses of medicinal products in Europe*. 2010, EMA.
4. European Commision, *Regulation (EC) No. 1901/2006 of the European Parliament and of the Council of 12 December 2006 on medicinal products for paediatric use and amending Regulation (EEC) No 1768/92, Directive 2001/20/EC, Directive 2001/83/EC and Regulation (EC) No 726/2004*. 2006, European Commision: Strasbourg. p. 1-19.
5. Ruggieri, L., et al., *Successful private-public funding of paediatric medicines research: lessons from the EU programme to fund research into off-patent medicines.* Eur J Pediatr, 2015. **174**(4): p. 481-91.
6. EMA/PDCO *Revised priority list for studies into off-patent paediatric medicinal products*. 2013.
7. British Paediatric Association, *The Care of Critically Ill Children. Report of a Multidisciplinary Working Party on Intensive Care* 1993, BPA: London.
8. UK Paediatric Intensive Care Society, *PICS Standards.* 2001.
9. Playfor, S.D. and H. Vyas, *Sedation in critically ill children.* Curr Paediatr, 2000. **10**(1): p. 1-4.
10. Tobias, J.D. and G.E. Rasmussen, *Pain management and sedation in the pediatric intensive care unit.* Pediatr Clin North Am, 1994. **41**(6): p. 1269-92.
11. Baarslag, M.A., et al., *Pharmacological sedation management in the paediatric intensive care unit.* J Pharm Pharmacol, 2017. **69**(5): p. 498-513.
12. Playfor, S., et al., *Consensus guidelines on sedation and analgesia in critically ill children.* Intensive Care Med, 2006. **32**(8): p. 1125-36.

For peer review only

13. Playfor, S., D. Thomas, and I. Choonara, *Recollection of children following intensive care.* Archives of Disease in Childhood, 2000. **83**(5): p. 445-448.

14. Klupp, H., et al., *Effects of Clonidine on central sympathetic tone.* Eur J Pharmacol, 1970. **10**(2): p. 225-9.

15. Drew, G.M. and S.B. Whiting, *Evidence for two distinct types of postsynaptic alpha-adrenoceptor in vascular smooth muscle in vivo.* Br J Pharmacol, 1979. **67**(2): p. 207-15.

16. Ramesh, V.J., N. Bhardwaj, and Y.K. Batra, *Comparative study of oral clonidine and diazepam as premedicants in children.* Int J Clin Pharmacol Ther, 1997. **35**(5): p. 218-21.

17. Nishina, K., et al., *Clonidine in paediatric anaesthesia.* Paediatr Anaesth, 1999. **9**(3): p. 187-202.

18. Hoffman, W.E., et al., *Dexmedetomidine improves neurologic outcome from incomplete ischemia in the rat. Reversal by the alpha 2-adrenergic antagonist atipamezole.* Anesthesiology, 1991. **75**(2): p. 328-32.

19. Laudenbach, V., et al., *Effects of alpha(2)-adrenoceptor agonists on perinatal excitotoxic brain injury: comparison of clonidine and dexmedetomidine.* Anesthesiology, 2002. **96**(1): p. 134-41.

20. Yuan, S.Z., et al., *Hypoxic-ischaemic brain damage in immature rats: effects of adrenoceptor modulation.* Eur J Paediatr Neurol, 2001. **5**(1): p. 29-35.

21. Donello, J.E., et al., *alpha(2)-Adrenoceptor agonists inhibit vitreal glutamate and aspartate accumulation and preserve retinal function after transient ischemia.* J Pharmacol Exp Ther, 2001. **296**(1): p. 216-23.

22. Zhang, Y., *Clonidine preconditioning decreases infarct size and improves neurological outcome from transient forebrain ischemia in the rat.* Neuroscience, 2004. **125**(3): p. 625-31.

23. Wolf, A., *Development and evaluation of pain and the stress response.*, in *Paediatric Anaesthesia*, B. Bissonnette and B. Dalens, Editors. 2002, McGraw-Hill: New York. p. 186-200.

For peer review only

24. Burch, M., et al., *Influence of cardiopulmonary bypass on water balance hormones in children.* Br Heart J, 1992. **68**(3): p. 309-12.

25. Dorman, T., et al., *Effects of clonidine on prolonged postoperative sympathetic response.* Crit Care Med, 1997. **25**(7): p. 1147-52.

26. Kulka, P.J., M. Tryba, and M. Zenz, *Preoperative alpha2-adrenergic receptor agonists prevent the deterioration of renal function after cardiac surgery: results of a randomized, controlled trial.* Crit Care Med, 1996. **24**(6): p. 947-52.

27. Hohage, H., E. Schlatter, and J. Greven, *Effects of moxonidine and clonidine on renal function and blood pressure in anesthetized rats.* Clin Nephrol, 1997. **47**(5): p. 316-24.

28. Wijeysundera, D.N., J.S. Naik, and W.S. Beattie, *Alpha-2 adrenergic agonists to prevent perioperative cardiovascular complications: a meta-analysis.* Am J Med, 2003. **114**(9): p. 742-52.

29. Pohl-Schickinger, A., et al., *Intravenous clonidine infusion in infants after cardiovascular surgery.* Paediatr Anaesth, 2008. **18**(3): p. 217-22.

30. Martin, J., et al., *Evidence and consensus-based German guidelines for the management of analgesia, sedation and delirium in intensive care--short version.* Ger Med Sci, 2010. **8**: p. Doc02.

31. Ambrose, C., et al., *Intravenous clonidine infusion in critically ill children: dose-dependent sedative effects and cardiovascular stability.* Br J Anaesth, 2000. **84**(6): p. 794-6.

32. Arenas-Lopez, S., et al., *Use of oral clonidine for sedation in ventilated paediatric intensive care patients.* Intensive Care Med, 2004. **30**(8): p. 1625-9.

33. Wolf, A., et al., *Prospective multicentre randomised, double-blind, equivalence study comparing clonidine and midazolam as intravenous sedative agents in critically ill children: the SLEEPS (Safety profiLe, Efficacy and Equivalence in Paediatric intensive care Sedation) study.* Health Technol Assess, 2014. **18**(71): p. 1-212.

34. Hunseler, C., et al., *Continuous infusion of clonidine in ventilated newborns and infants: a randomized controlled trial.* Pediatr Crit Care Med, 2014. **15**(6): p. 511-22.

For peer review only

35. Duffett, M., et al., *Clonidine in the sedation of mechanically ventilated children: A pilot randomized trial.* J Crit Care, 2014. **29**(5): p. 758-63.

36. Akcan-Arikan, A., et al., *Modified RIFLE criteria in critically ill children with acute kidney injury.* Kidney Int, 2007. **71**(10): p. 1028-35.

37. Ricci, Z. and C. Ronco, *Neonatal RIFLE.* Nephrol Dial Transplant, 2013. **28**(9): p. 2211-4.

38. Seri, I. and J. Evans, *Controversies in the diagnosis and management of hypotension in the newborn infant.* Curr Opin Pediatr, 2001. **13**(2): p. 116-23.

39. Goldstein, B., et al., *International pediatric sepsis consensus conference: definitions for sepsis and organ dysfunction in pediatrics.* Pediatr Crit Care Med, 2005. **6**(1): p. 2-8.

40. European Union, *European Regulation on clinical trials (EU) No 536/2014 of the European Parliament and of the Council of 16 April 2014 on clinical trials on medicinal products for human use, and repealing Directive 2001/20/EC.* 2014.

41. Hall, J.E., T.D. Uhrich, and T.J. Ebert, *Sedative, analgesic and cognitive effects of clonidine infusions in humans.* Br J Anaesth, 2001. **86**(1): p. 5-11.

42. Sheng, Y. and J.F. Standing, *Pharmacokinetic reason for negative results of clonidine sedation in long-term-ventilated neonates and infants.* Pediatr Crit Care Med, 2015. **16**(1): p. 92-3.

43. Ista, E., et al., *Assessment of sedation levels in pediatric intensive care patients can be improved by using the COMFORT "behavior" scale.* Pediatr Crit Care Med, 2005. **6**(1): p. 58-63.

44. von Baeyer, C.L. and L.J. Spagrud, *Systematic review of observational (behavioral) measures of pain for children and adolescents aged 3 to 18 years.* Pain, 2007. **127**(1-2): p. 140-50.

45. Ista, E., et al., *Psychometric evaluation of the Sophia Observation withdrawal symptoms scale in critically ill children.* Pediatr Crit Care Med, 2013. **14**(8): p. 761-9.

46. Bayley, N., *Bayley Scales of Infant Development, 2nd edition.* 1993, San Antonio, TX: The Psychological Corporation.

For peer review only

47. de Wildt, S.N., et al., *Pharmacodynamics of midazolam in pediatric intensive care patients.* Ther Drug Monit, 2005. **27**(1): p. 98-102.

48. Nederlands Kenniscentrum voor Farmacotherapie bij Kinderen, *Kinderformularium*. 2014.

49. Klingenberg, B., *A new and improved confidence interval for the Mantel-Haenszel risk difference.* Stat Med, 2014. **33**(17): p. 2968-83.

50. Ueckert, S., et al., *Improved utilization of ADAS-cog assessment data through item response theory based pharmacometric modeling.* Pharm Res, 2014. **31**(8): p. 2152-65.

51. European Commission, *Ethical Considerations for Clinical Trials on Medicinal Products Conducted with the Paediatric Population*. 2008.

52. European Union, *Ethical considerations for clinical trials on medicinal products conducted with the paediatric population.* Eur J Health Law, 2008. **15**(2): p. 223-50.

53. ICH Expert Working Group, *Guideline for Good Clinical Practice*. 1996.

54. European Commission, *Directive 2001/20/EC of the European Parliament and of the Council of 4 April 2001 on the approximation of the laws, regulations and administrative provisions of the Member States relating to the implementation of good clinical practice in the conduct of clinical trials on medicinal products for human use.* 2001.

55. European Commision, *Detailed guidance on the application format and documentation to be submitted in an application for an Ethics Committee opinion on the clinical trial on medicinal products for human use. Revision 1.* 2006.

56. European Commision, *Directive 95/46/EC of the European Parliament and of the Council of 24 October 1995 on the protection of individuals with regard to the processing of personal data and on the free movement of such data*. 1995.

57. Council for International Organizations of Medical Sciences (CIOMS)-WHO, *International Ethical Guidelines for Biomedical Research Involving Human Subjects.* 2002.

58. Council of Europe, *Additional Protocol to the Convention on Human Rights and Biomedicine, concerning Biomedical Research.* 2005.

For peer review only

59. UNESCO, *International Declaration on Human Genetic Data*. 2003.

60. Kennedy-Martin, T., et al., *A literature review on the representativeness of randomized controlled trial samples and implications for the external validity of trial results.* Trials, 2015. **16**: p. 495.

61. Heidmets, L.T., et al., *Blood loss related to participation in pharmacokinetic study in preterm neonates.* Neonatology, 2011. **100**(2): p. 111-5.

62. Bergendahl, H.T., S. Eksborg, and P.A. Lonnqvist, *Low-dose intravenous clonidine in children: plasma concentrations and haemodynamic response.* Acta Anaesthesiol Scand, 1997. **41**(3): p. 381-4.

63. Hanning, S.M., et al., *Quality and clinical supply considerations of Paediatric Investigation Plans for IV preparations-A case study with the FP7 CloSed project.* Int J Pharm, 2016.

64. Lasagna, L., *Problems in publication of clinical trial methodology.* Clin Pharmacol Ther, 1979. **25**(5 Pt 2): p. 751-3.

For peer review only

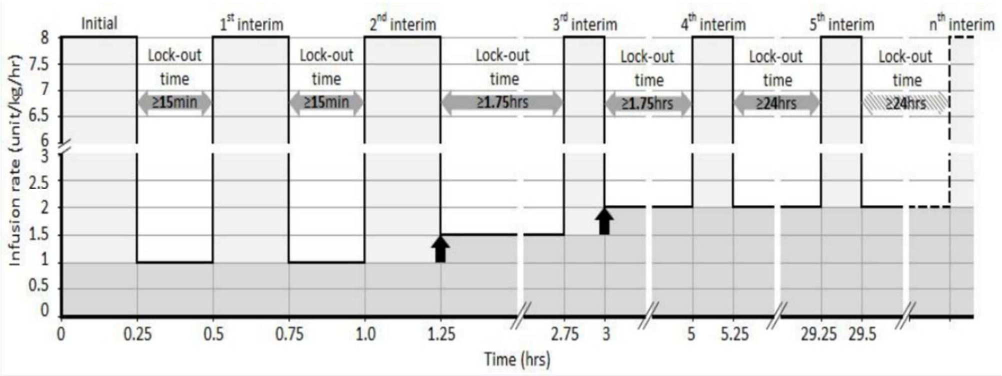

Figure 1. Minimum time line of infusion rate increases for all loading doses of IMP administered for subjects (half doses in neonates).

322x120mm (300 x 300 DPI)

For peer review only

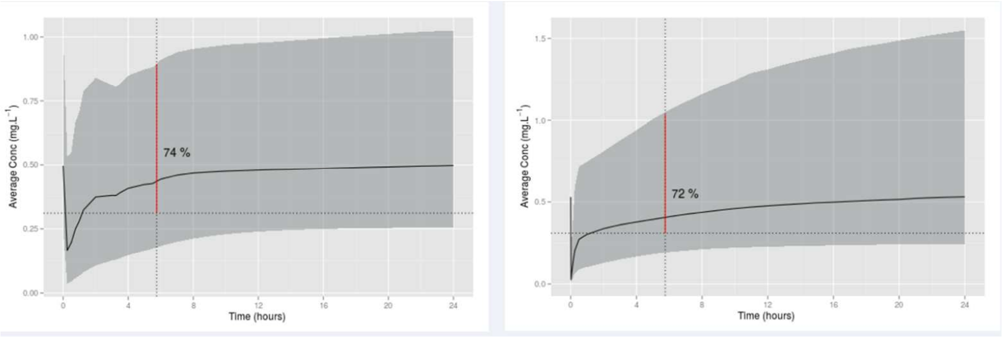

Figure 2. The proportion of average midazolam plasma concentration above the target value (0.311 mg/l) after the time of 5th bolus according to the proposed dose scheme(left) and the maximum Dutch recommended dosing regimen(right).

330x110mm (300 x 300 DPI)

For peer review only

SPIRIT

STANDARD PROTOCOL ITEMS: RECOMMENDATIONS FOR INTERVENTIONAL TRIALS

SPIRIT 2013 Checklist: Recommended items to address in a clinical trial protocol and related documents*

Section/item	Item No	Description	Addressed on page number
**Administrative information**			
Title	1	Descriptive title identifying the study design, population, interventions, and, if applicable, trial acronym	1____________
Trial registration	2a	Trial identifier and registry name. If not yet registered, name of intended registry	2____________
	2b	All items from the World Health Organization Trial Registration Data Set	-_____________
Protocol version	3	Date and version identifier	36___________
Funding	4	Sources and types of financial, material, and other support	38___________
Roles and responsibilities	5a	Names, affiliations, and roles of protocol contributors	1-2, 36-37____
	5b	Name and contact information for the trial sponsor	38___________
	5c	Role of study sponsor and funders, if any, in study design; collection, management, analysis, and interpretation of data; writing of the report; and the decision to submit the report for publication, Including whether they will have ultimate authority over any of these activities	38___________
	5d	Composition, roles, and responsibilities of the coordinating centre, steering committee, endpoint adjudication committee, data management team, and other individuals or groups overseeing the trial, if applicable (see Item 21a for data monitoring committee)	28___________

For peer review only - http://bmjopen.bmj.com/site/about/guidelines.xhtml

**Introduction**			
Background and rationale	6a	Description of research question and justification for undertaking the trial, including summary of relevant studies (published and unpublished) examining benefits and harms for each intervention	5-9__________
	6b	Explanation for choice of comparators	9_____________
Objectives	7	Specific objectives or hypotheses	9_____________
Trial design	8	Description of trial design including type of trial (eg, parallel group, crossover, factorial, single group), allocation ratio, and framework (eg, superiority, equivalence, noninferiority, exploratory)	10-11________
**Methods: Participants, interventions, and outcomes**			
Study setting	9	Description of study settings (eg, community clinic, academic hospital) and list of countries where data will be collected. Reference to where list of study sites can be obtained	10___________
Eligibility criteria	10	Inclusion and exclusion criteria for participants. If applicable, eligibility criteria for study centres and individuals who will perform the interventions (eg, surgeons, psychotherapists)	10-11________
Interventions	11a	Interventions for each group with sufficient detail to allow replication, including how and when they will be administered	11-13________
	11b	Criteria for discontinuing or modifying allocated interventions for a given trial participant (eg, drug dose change in response to harms, participant request, or improving/worsening disease)	N/A
	11c	Strategies to improve adherence to intervention protocols, and any procedures for monitoring adherence (eg, drug tablet return, laboratory tests)	N/A_________
	11d	Relevant concomitant care and interventions that are permitted or prohibited during the trial	-_____________
Outcomes	12	Primary, secondary, and other outcomes, including the specific measurement variable (eg, systolic blood pressure), analysis metric (eg, change from baseline, final value, time to event), method of aggregation (eg, median, proportion), and time point for each outcome. Explanation of the clinical relevance of chosen efficacy and harm outcomes is strongly recommended	15-17________
Participant timeline	13	Time schedule of enrolment, interventions (including any run-ins and washouts), assessments, and visits for participants. A schematic diagram is highly recommended (see Figure)	12-14________

For peer review only - http://bmjopen.bmj.com/site/about/guidelines.xhtml

Sample size	14	Estimated number of participants needed to achieve study objectives and how it was determined, including clinical and statistical assumptions supporting any sample size calculations	18-19________
Recruitment	15	Strategies for achieving adequate participant enrolment to reach target sample size	10-12_________
**Methods: Assignment of interventions (for controlled trials)**			
Allocation:			
Sequence generation	16a	Method of generating the allocation sequence (eg, computer-generated random numbers), and list of any factors for stratification. To reduce predictability of a random sequence, details of any planned restriction (eg, blocking) should be provided in a separate document that is unavailable to those who enrol participants or assign interventions	23___________
Allocation concealment mechanism	16b	Mechanism of implementing the allocation sequence (eg, central telephone; sequentially numbered, opaque, sealed envelopes), describing any steps to conceal the sequence until interventions are assigned	23___________
Implementation	16c	Who will generate the allocation sequence, who will enrol participants, and who will assign participants to interventions	23___________
Blinding (masking)	17a	Who will be blinded after assignment to interventions (eg, trial participants, care providers, outcome assessors, data analysts), and how	23___________
	17b	If blinded, circumstances under which unblinding is permissible, and procedure for revealing a participant's allocated intervention during the trial	23-24_________
**Methods: Data collection, management, and analysis**			
Data collection methods	18a	Plans for assessment and collection of outcome, baseline, and other trial data, including any related processes to promote data quality (eg, duplicate measurements, training of assessors) and a description of study instruments (eg, questionnaires, laboratory tests) along with their reliability and validity, if known. Reference to where data collection forms can be found, if not in the protocol	15-23___________
	18b	Plans to promote participant retention and complete follow-up, including list of any outcome data to be collected for participants who discontinue or deviate from intervention protocols	20___________

For peer review only - http://bmjopen.bmj.com/site/about/guidelines.xhtml

Data management	19	Plans for data entry, coding, security, and storage, including any related processes to promote data quality (eg, double data entry; range checks for data values). Reference to where details of data management procedures can be found, if not in the protocol	-____________
Statistical methods	20a	Statistical methods for analysing primary and secondary outcomes. Reference to where other details of the statistical analysis plan can be found, if not in the protocol	20-23________
	20b	Methods for any additional analyses (eg, subgroup and adjusted analyses)	20-23________
	20c	Definition of analysis population relating to protocol non-adherence (eg, as randomised analysis), and any statistical methods to handle missing data (eg, multiple imputation)	20___________
**Methods: Monitoring**			
Data monitoring	21a	Composition of data monitoring committee (DMC); summary of its role and reporting structure; statement of whether it is independent from the sponsor and competing interests; and reference to where further details about its charter can be found, if not in the protocol. Alternatively, an explanation of why a DMC is not needed	28___________
	21b	Description of any interim analyses and stopping guidelines, including who will have access to these interim results and make the final decision to terminate the trial	-____________
Harms	22	Plans for collecting, assessing, reporting, and managing solicited and spontaneously reported adverse events and other unintended effects of trial interventions or trial conduct	17___________
Auditing	23	Frequency and procedures for auditing trial conduct, if any, and whether the process will be independent from investigators and the sponsor	-____________
**Ethics and dissemination**			
Research ethics approval	24	Plans for seeking research ethics committee/institutional review board (REC/IRB) approval	28___________
Protocol amendments	25	Plans for communicating important protocol modifications (eg, changes to eligibility criteria, outcomes, analyses) to relevant parties (eg, investigators, REC/IRBs, trial participants, trial registries, journals, regulators)	-____________

For peer review only - http://bmjopen.bmj.com/site/about/guidelines.xhtml

Consent or assent	26a	Who will obtain informed consent or assent from potential trial participants or authorised surrogates, and how (see Item 32)	27-28________
	26b	Additional consent provisions for collection and use of participant data and biological specimens in ancillary studies, if applicable	27-28________
Confidentiality	27	How personal information about potential and enrolled participants will be collected, shared, and maintained in order to protect confidentiality before, during, and after the trial	-_____________
Declaration of interests	28	Financial and other competing interests for principal investigators for the overall trial and each study site	38____________
Access to data	29	Statement of who will have access to the final trial dataset, and disclosure of contractual agreements that limit such access for investigators	-_____________
Ancillary and post-trial care	30	Provisions, if any, for ancillary and post-trial care, and for compensation to those who suffer harm from trial participation	-_____________
Dissemination policy	31a	Plans for investigators and sponsor to communicate trial results to participants, healthcare professionals, the public, and other relevant groups (eg, via publication, reporting in results databases, or other data sharing arrangements), including any publication restrictions	28____________
	31b	Authorship eligibility guidelines and any intended use of professional writers	N/a___________
	31c	Plans, if any, for granting public access to the full protocol, participant-level dataset, and statistical code	-_____________
**Appendices**			
Informed consent materials	32	Model consent form and other related documentation given to participants and authorised surrogates	-_____________
Biological specimens	33	Plans for collection, laboratory evaluation, and storage of biological specimens for genetic or molecular analysis in the current trial and for future use in ancillary studies, if applicable	-_____________

*It is strongly recommended that this checklist be read in conjunction with the SPIRIT 2013 Explanation & Elaboration for important clarification on the items. Amendments to the protocol should be tracked and dated. The SPIRIT checklist is copyrighted by the SPIRIT Group under the Creative Commons "Attribution-NonCommercial-NoDerivs 3.0 Unported" license.

For peer review only - http://bmjopen.bmj.com/site/about/guidelines.xhtml
