# Supplementary material for: The CLOSED trial; CLOnidine compared with midazolam for SEDation of paediatric patients in the intensive care unit: study protocol for a multicentre randomised controlled trial
Source: BMJ Open. 2017 Jun 21;7(6):e016031. doi: 10.1136/bmjopen-2017-016031 (PMC5726127; doi:10.1136/bmjopen-2017-016031)
Supplement: Reviewer comments [file bmjopen-2017-016031.reviewer_comments.pdf]

PEER REVIEW HISTORY

BMJ Open publishes all reviews undertaken for accepted manuscripts. Reviewers are asked to complete a checklist review form (**http://bmjopen.bmj.com/site/about/resources/checklist.pdf**) and are provided with free text boxes to elaborate on their assessment. These free text comments are reproduced below.

ARTICLE DETAILS

**TITLE (PROVISIONAL)**	The CLOSED- trial; CLOnidine compared to midazolam for SEDation of paediatric patients in the intensive care unit: study protocol for a multicentre randomised controlled trial
**AUTHORS**	Neubert, Antje; Baarslag, Manuel; van Dijk, Monique; van Rosmalen, Joost; Standing, Joseph; Sheng, Yucheng; Rascher, Wolfgang; Roberts, Deborah; Winslade, Jackie; Rawcliffe, Louise; Hanning, Sara; Metsvaht, Tuuli; Giannuzzi, Viviana; Larsson, Peter; Pokorná, Pavla; Simonetti, Alessandra; Tibboel, Dick

VERSION 1 - REVIEW

**REVIEWER**	Sukanya Mitra<br>Government Medical College & Hospital, Chandigarh, India
**REVIEW RETURNED**	02-Feb-2017

**GENERAL COMMENTS**	Well designed and clinically relevant.

**REVIEWER**	Yahya Shehabi<br>Monash Uni<br>Australia
**REVIEW RETURNED**	28-Feb-2017

**GENERAL COMMENTS**	Thank you for asking to review the protocol and statistical analysis plan for this very ambitious and well planned study.<br>The manuscript is well written with sufficient details to inform the details of the design, research methods, population chosen, interventions and outcomes. Investigators also highlighted the significant challenge in sedation trials in general and in particular in the paediatric population.<br>Significant preliminary preparation has been conducted and the trial is already recruiting in at least one centre. In this regard I didn't see why there are only 4 more centres planned to join the study?<br>I also have the following comments which should be addressed in study limitations:<br>1. The choice of a non-inferiority objective with midazolam is interesting. Midazolam although still very popular in PCU sedation, is not truly gold standard and there are many issues and problems associated with its use. So I can't see the point of testing for non-inferiority with an intervention already considered sub-optimal.<br>2. There are distinct groups of patients on the inclusion criteria, post-surgery, medical and transfer from other hospitals. This may create heterogeneity in at multiple levels, first, time to receiving study medications, second, severity of illness and third baseline contamination. Therefore, in the absence of data to suggest prospective numbers to be recruited in each group, it is important to

	have equivalent number of patients in the 3 groups. This may be done by stratification at randomisation at least between surgical and medical and preferably (if high numbers are anticipated) the out of hospital transfers.<br>3. I believe the window for recruitment of 72 hours is very long and I would perhaps consider a 24 hours window for ALL patients.<br>4. The feasibility of the study may be an issue for 2 reasons; first, the exclusion criteria are numerous and exclude many patients unnecessarily. Second, no indication given in the protocol of expected recruitment rate in different centers. Given inclusion/exclusion and consent refusal this is a significant issue.<br>5. The external validity and generalisability of the results will be limited conserving the extensive exclusion list given.

VERSION 1 – AUTHOR RESPONSE

Reviewer: 1
Reviewer Name: Sukanya Mitra
Institution and Country: Government Medical College & Hospital, Chandigarh, India Competing Interests: None declared

Well designed and clinically relevant.

Reviewer: 2
Reviewer Name: Yahya Shehabi
Institution and Country: Monash University, Australia Competing Interests: None declared

Thank you for asking to review the protocol and statistical analysis plan for this very ambitious and well planned study.

The manuscript is well written with sufficient details to inform the details of the design, research methods, population chosen, interventions and outcomes. Investigators also highlighted the significant challenge in sedation trials in general and in particular in the paediatric population.

Significant preliminary preparation has been conducted and the trial is already recruiting in at least one centre. In this regard I didn't see why there are only 4 more centres planned to join the study?
Dear Dr. Shehabi, thank you for this comment. The anticipated inclusion rates showed that these 5 recruiting centres would be enough to reach our sample size.

I also have the following comments which should be addressed in study limitations:

1. The choice of a non-inferiority objective with midazolam is interesting. Midazolam although still very popular in PCU sedation, is not truly gold standard and there are many issues and problems associated with its use. So I can't see the point of testing for non-inferiority with an intervention already considered sub-optimal.
Thank you, we have added a paragraph on study limitations and we addressed this point.This is the full content of this paragraph:

Despite the careful design of this trial, some limitations exist. First, we have chosen a non-inferiority design for this trial because based on clinical experience and limited available data, we estimate the difference in sedation success rate very low. This would mean that showing superiority of clonidine requires a large sample size and thus compromising feasibility. This design, however, means that we cannot demonstrate possible superiority of clonidine. However, if equal efficacy is shown under safe

circumstances, clonidine can be licensed for sedation in the PICU.
Second, the recruitment window has been set on 72 hours. This could lead to confounding as having a large time window could increase the risk of tolerance and withdrawal symptoms. A smaller recruitment window may reduce these risks but parents need some time to consider participation and not every study site has 24/7 research nurse availability.
Third, this trial has an extensive list of exclusion criteria. This could compromise the external validity of the results and could complicate implementation of the results in new guidelines. This is a known limitation of almost any randomized controlled trial[60]. However, these exclusion criteria are based on expected elements affecting both primary outcome and patient safety. Our primary endpoint is based on a validated behavioural scale. Therefore, any disease status that can cause many fluctuations in behaviour (such as neurological injury) needed to be excluded from this trial for the results to be valid. The same holds for safety endpoints such as cardiovascular stability. The decision to allow for inotropes/vasopressors in the second amendment is a big step towards generalisability of the results.

2. There are distinct groups of patients on the inclusion criteria, post-surgery, medical and transfer from other hospitals. This may create heterogeneity in at multiple levels, first, time to receiving study medications, second, severity of illness and third baseline contamination. Therefore, in the absence of data to suggest prospective numbers to be recruited in each group, it is important to have equivalent number of patients in the 3 groups. This may be done by stratification at randomisation at least between surgical and medical and preferably (if high numbers are anticipated) the out of hospital transfers.
Thank you for this suggestion. We agree on the statement that stratification of these categories can be useful if the categories are strongly associated with the primary endpoints. This can be doubted, however, as patients transferred from other hospitals may be randomized almost as quickly as postsurgical patients (at least in the centres in urbanized regions). It may also take time for postsurgical patients to be in need of sedation as they may be influenced by the anaesthesia. Severity-of-illness data are being collected.
Randomisation between these groups may also be problematic due to the varying case mix in the centres, and would compromise the study's feasibility.

3. I believe the window for recruitment of 72 hours is very long and I would perhaps consider a 24 hours window for ALL patients.
Thank you for this suggestion. We have considered this duration, but from prior research experience we know that informed consent processed may take longer and that 24/7 research nurse availability is necessary to achieve this short window. This is unfortunately not the case in most centres. We have addressed this in the new 'Study limitations' section.

4. The feasibility of the study may be an issue for 2 reasons; first, the exclusion criteria are numerous and exclude many patients unnecessarily. Second, no indication given in the protocol of expected recruitment rate in different centers. Given inclusion/exclusion and consent refusal this is a significant issue.
Thank you for this valuable comment. Yes, the feasibility is an issue, mainly because of the numerous exclusion criteria. We now have added a 'Reflection' section discussing this issue. Also,we added the expected recruitment rate of the trial in total. Specific details on the rate per centre have been left out for brevity purposes.
The full content of the 'Reflection' section is:
After 9 months of recruiting, we included far fewer children than anticipated. Therefore, two amendments have now been added to potentially increase the number of eligible patients. Unfortunately, recruitment did not improve. . Even though we had been warned by the early discontinuation of the SLEEPS trial[33], we faced other challenges. For example, many postoperative neonates do not need any additional sedation to IV paracetamol and continuous IV morphine. Also,

parents are reluctant to participate Either they feel their child is not stable enough, or their child is finally stable and therefore changes in treatment for study purposes are not welcomed.
Also, the anticipated recruitment rate was based on the number of admitted ventilated patients in previous years. However, this turned out to be a significant overestimation, a phenomenon also known as Lasagna's Law[64]. These challenges have a big impact on the feasibility of the trial and force us to consider alternative options. We will open (at least) two new recruitment sites, Bari (Italy) and Tallinn (Estonia). Furthermore, we need to rethink the primary objective of this study and may change it to a PK-PD study for which 50 patients in each arm are sufficient instead of the original 150 patients per arm.
The lessons learned from both the SLEEPS trial and this trial are important for further investigations in paediatric critical care and careful preparation is warranted for any trial performed in this population. This preparation should at least include adequate piloting over a longer period. In our experience, we monitored eligible patients over one month in one centre and expected no significant recruitment problems. This monitoring was performed during winter time, when many patients with respiratory viral infections in need of mechanical ventilation were admitted. This caused our expectations of eligible patients to be high, as if we had performed this during summer time, we may have been warned earlier.
Other aspects to enhance the number of included patients are adequate staff training and motivation, collaboration with investigators having experience in paediatric critical care trials and keep the exclusion criteria of a trial to a minimum.

5. The external validity and generalisability of the results will be limited conserving the extensive exclusion list given.
We agree fully on this and it is a limitation of many randomized controlled trials. We looked carefully into amending the exclusion criteria but only with limited results. We have addressed this in the 'study limitations' section.
